# From Random Numbers to Random Objects

**DOI:** 10.3390/e24070928

**Published:** 2022-07-04

**Authors:** Behrouz Zolfaghari, Khodakhast Bibak, Takeshi Koshiba

**Affiliations:** 1 Cyber Science Lab, School of Computer Science, University of Guelph, Guelph, ON N1G 2W1, Canada; behrouz@cybersciencelab.org; 2 Department of Computer Science and Software Engineering, Miami University, Oxford, OH 45056, USA; 3 Department of Mathematics, Faculty of Education and Integrated Arts and Sciences, Waseda University, Tokyo 169-8050, Japan; tkoshiba@waseda.jp

**Keywords:** integer compositions, Linear Feedback Shift Registers (LFSRs), parallel LFSRs, random number generation, random object generation, S-restricted random number generator, 65C10, 94A60, 97P60

## Abstract

Many security-related scenarios including cryptography depend on the random generation of passwords, permutations, Latin squares, CAPTCHAs and other types of non-numerical entities. Random generation of each entity type is a different problem with different solutions. This study is an attempt at a unified solution for all of the mentioned problems. This paper is the first of its kind to pose, formulate, analyze and solve the problem of *random object generation* as the general problem of generating random non-numerical entities. We examine solving the problem via connecting it to the well-studied *random number generation* problem. To this end, we highlight the challenges and propose solutions for each of them. We explain our method using a case study; random Latin square generation.

## 1. Introduction and Basic Concepts

Random password generation [1], random CAPTCHA generation (Completely Automated Public Turing test to tell Computers and Humans Apart) [2,3], random permutation generation [4,5] and random Latin square generation [6,7] are critical cryptographic problems. There are many other similar problems that play critical roles in different branches of science and technology. Each of these problems is about random generation of *non-numeric entity*, to all of which we refer using the general name *object* in this paper. Although the mentioned problems may appear conceptually similar, solutions proposed for each of them may not be applicable to others. In this paper, we unify these problems and formalize the general problem of *random object generation* as the problem of generating random instances of any *non-numeric* entity type. Afterwards, we examine solving the newly-posed problem via connecting it to the well-known problem of *random number generation*. The primary idea behind our proposed approach is to assign numeric codes to objects, and generate random object codes using Random Number Generators (RNGs) (Please see more details in Section 2.3). However, there are challenges that need to be resolved. We analyze and resolve each of these challenges (Section 2.2).

In this study, we first formalize the problem of *random object generation*. Then, we introduce the notion of *S*-restricted RNGs as RNGs capable of generating random numbers derived from an arbitrary set *S*. We show how the use of *S*-restricted RNGs along with proper encoding schemes can lead to a solution to the problem of *random object generation*. We present our proposed *random object generation* method based on these two components. In the next step, we will propose a method based on integer compositions for automatic design of parallel LFSRs. Furthermore, we present an architecture based on parallel LFSRs (as parallel RNGs) for designing *S*-restricted RNGs. Lastly, we present a case study for illustrating the proposed method. We design a circuit for generating random Latin squares of order 4 with the help of a novel encoding scheme.

Before formalizing the ROG problem and discussing our approach towards its solution, we need to introduce some concepts in the next subsection.

### 1.1. Basic Concepts

In the following, we define integer composition, parallel LFSRs, *S*-restricted RNGs and Latin squares, which will be used later while describing our solution to the problem of *random object generation*.

#### 1.1.1. Integer Compositions

A composition *C* of a positive integer *n* is a sequence of positive integers called parts (summands) that add up to *n*. Different aspects and applications of integer compositions have been studied by researchers [8]. In this paper, we represent compositions by tuples, and denote the set of compositions of a positive integer *n* by C(n). As an example, C(3)={(1,1,1),(1,2),(2,1),(3)}. It can easily be shown that $\left|C\left(n\right)\right|=2^{n-1}$ for every n∈N. For a composition $C=\left(C_{1},C_{2},\ldots ,C_{l-1},C_{l}\right)$ and i∈{1,2,…,l}, we define $\tilde{C}=\left(C_{l},C_{l-1},\ldots ,C_{2},C_{1}\right)$, $\lambda \left(C,i\right)=C_{i}$, $first\left(C\right)=\lambda \left(C,1\right)=C_{1}$, $last\left(C\right)=\lambda \left(C,l\right)=C_{l}$, length(C)=l, $f^{-}\left(C\right)=\left(C_{2},\ldots ,C_{l-1},C_{l}\right)$ and $l^{-}\left(C\right)=\left(C_{1},C_{2},\ldots ,C_{l-1}\right)$. Moreover, for a positive integer *x*, we represent $\left(x,C_{1},C_{2},\ldots ,C_{l-1},C_{l}\right)$ and $\left(C_{1},C_{2},\ldots ,C_{l-1},C_{l},x\right)$ by x;C and C;x, respectively. It is immediate that $f^{-}\left(C\right)=l^{-}\left(C\right)=\varphi =\left(\right)$ if length(C)=1, and  x;C=C;x=(x) if C=φ.

An *S*-restricted composition of *n* is a composition in which all parts are chosen from a given set S⊂{1,2,…,n}. Several properties of *S*-restricted compositions as well as related problems have been investigated in various research works [9]. We use $C^{\left(S\right)}\left(n\right)$ to represent the set of *S*-restricted compositions of *n*. For example, $C^{\left(\{1,2\}\right)}\left(3\right)=\left\{\left(1,1,1\right),\left(1,2\right),\left(2,1\right)\right\}$. Different types and aspects of *S*-restricted compositions have been investigated by researchers [10]. However, to the best of our knowledge, there is no closed form solution for calculating the number of *S*-restricted compositions.

A palindromic composition (a palindrome) of *n* is a composition of *n* that is read in the same way from the left and the right. The notation $C_{P}\left(n\right)$ is used in this paper to represent the set of palindromic compositions of *n*. For example, $C_{P}\left(3\right)=\left\{\left(1,1,1\right),\left(3\right)\right\}$. It can easily be shown that $|C_{P}\left(n\right)|=2^{\left\lfloor \frac{n}{2}\right\rfloor }$. An *S*-restricted palindromic composition (*S*-restricted palindrome) of *n* is a palindrome in which parts are chosen from a given set S⊂{1,2,…,n}. For example, $C_{P}^{\left\{1,2\right\}}\left(3\right)=\left\{\left(1,1,1\right)\right\}$.

#### 1.1.2. Parallel LFSRs

An LFSR is constructed of a shift register for keeping the state of the LFSR, along with a feedback loop, which controls the state transition. We denote an LFSR with *n* flip-flops and the generating polynomial *G* by $P_{n}\left(G,M\right)$, where *M* is the input string. There are two common representations for $P_{n}\left(G,M\right)$; Fibonacci representation and Galois representation. Galois and Fibonacci representations are mathematically equivalent in the sense that every sequence generated by a Fibonacci LFSR can be generated by a Galois LFSR, and vice versa [11,12]. Since the delay in the corresponding logic circuit is not dependent on the size of the LFSR, we choose to use Galois LFSR defined by Equation (1):

(1) $$F_{i}\left(k+1\right)=\left\{\begin{array}{cc} F_{n}\left(k\right)+M_{k}, & i=1,\\ F_{i-1}\left(k\right)+G_{i-1}F_{n}\left(k\right), & i\in \{2,3,\ldots ,n\}. \end{array}\right.$$

**Figure 1:**
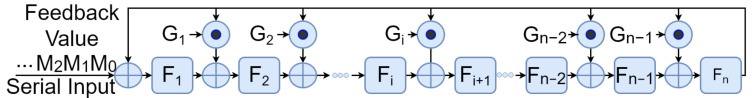
A programmable LFSR of size *n*.

In Equation (1), *n* is referred to as the size of the LFSR, and the vector $S^{k}\left(P_{n}\left(G,M\right)\right)=\left[F_{1}\left(k\right),F_{2}\left(k\right),\ldots ,F_{n}\left(k\right)\right]$ is called the $k^{th}$ state of the LFSR. Especially, $S^{0}\left(P_{n}\left(G,M\right)\right)=\left[F_{1}\left(0\right),F_{2}\left(0\right),\ldots ,F_{n}\left(0\right)\right]$ is referred to as the initial state of the LFSR. An implementation of an LFSR of size *n* is called a programmable implementation if it is capable of working with any arbitrary generating polynomial of degree *n*. A programmable LFSR implemented on the basis of Equation (1) is shown in Figure 1.

**Figure 2:**
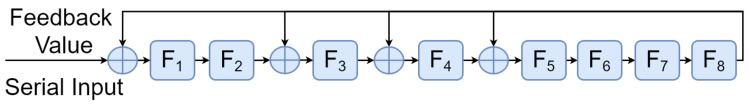
Galois and Fibonacci representations of an LFSR with generating polynomial $G=x^{8}+x^{4}+x^{3}+x^{2}+1$.

In the programmable LFSR shown in Figure 1, ⊕ and ⊙ represent GF(2) addition and multiplication, respectively, which can be implemented at the hardware layer using XOR and AND gates. Given a fixed generating polynomial, the ⊙ operations will obviously be no longer needed. Moreover, in an implementation with a fixed generating polynomial, the output of each ⊙ operation will be equal to 0 if its *G* input is 0. This eliminates the need for the corresponding ⊕ operation. As an example, an LFSR with generating polynomial $G=x^{8}+x^{4}+x^{3}+x^{2}+1$ is shown in Figure 2. In this paper, we use P to represent programmable LFSRs and L for LFSRs that work with a fixed generating polynomial.

An LFSR with *n* flip-flops and a primitive polynomial *G* guarantees to generate $2^{n}-1$ different numbers $\left\{1,2,\ldots ,2^{n}-1\right\}$ in each $2^{n}-1$ consecutive clock cycles by an order $r_{1},r_{2},\ldots ,r_{2^{n}-1}$, which depends on *G*. This capability makes it possible to use LFSRs in the design of pseudo-random number generators.

The definition of parallel LFSR (as suggested by the related literature) is a little tricky. A parallel LFSR with generating polynomial *G* and a sampling rate equal to *j* generates the sequence $r_{1},r_{j+1},r_{2j+1},\ldots$ where $r_{1},r_{2},\ldots ,r_{2^{n}-1}$ is the sequence generated by a serial LFSR with the same generating polynomial, and $r_{1}$ is equal to the seed. As stated by the definition, a parallel LFSR skips j−1 consecutive random numbers and outputs the $j^{th}$ one in each invocation or clock cycle.

For a positive integer *j*, an *n*-bit *j*-parallel LFSR $L_{n}^{j}\left(G,M\right)$ is defined by Equation (2):

(2) $$\forall k\geq 0:S^{k}\left(L_{n}^{j}\left(G,M\right)\right)=S^{kj}\left(L_{n}\left(G,M\right)\right).$$

In Equation (2), *j* is referred to as the sampling rate or the degree of parallelism. Parallel LFSRs are designed to achieve higher performance [13].

**Figure 3:**
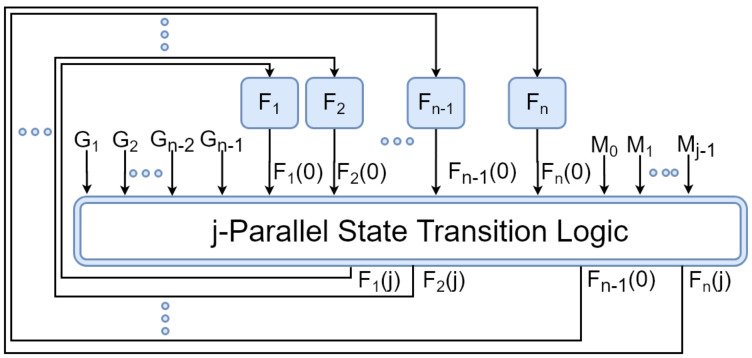
The block diagram of a programmable *n*-Bit *j*-parallel LFSR.

The LFSR $L_{n}^{1}\left(G,M\right)=L_{n}\left(G,M\right)$ is sometimes called a serial LFSR in order to distinguish it from parallel LFSRs. Similar to the case of serial LFSRs, parallel LFSRs can be implemented in a programmable way. We represent a programmable *n*-bit *j*-parallel LFSR by $P_{n}^{j}\left(G,M\right))$. Programmable LFSRS have been of particular interest for designers during the last few decades [14]. Figure 3 shows the block diagram of $P_{n}^{j}\left(G,M\right))$.

**Figure 4:**
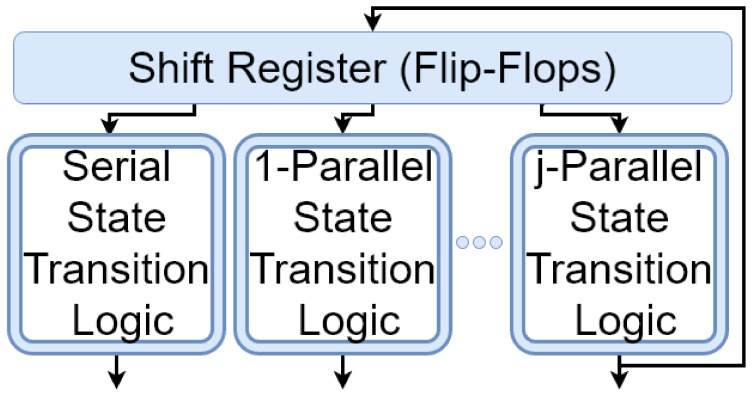
A *j*-parallel RNG based on parallel LFSRs.

In Figure 3, the state transition logic calculates $S^{1}\left(P_{n}^{j}\left(G,M\right)\right)=S^{j}\left(P_{n}\left(G,M\right)\right)$ using $S^{0}\left(P_{8}\left(G,M\right)\right)$, *G* and *M* in the first clock cycle. Afterwards, the state calculated in each cycle is considered as the initial state for the next cycle. In the rest of this paper, the term *"LFSR"* refers to non-programmable serial Galois-type LFSR, unless we clearly specify another type of LFSR. An LFSR with *n* flip-flops and a primitive polynomial *G* guarantees to generate $2^{n}-1$ different numbers $\left\{1,2,\ldots ,2^{n}-1\right\}$ in each $2^{n}-1$ consecutive clock cycles by an order $r_{1},r_{2},\ldots ,r_{2^{n}-1}$, which depends on *G*. This helps LFSRs be used as random number generators. Figure 4 shows how LFSRs and parallel LFSRs can be used to build a parallel RNG.

#### 1.1.3. *S*-Restricted RNGs

Assume the set *S* of positive integers, not necessarily consisting of consecutive integers. Furthermore, assume a random sequence of positive integers $I:{\left(i_{l}\right)}_{l=1}^{t}$ in a way that $\{i_{l}|i_{l}\in I\}=S$. Any RNG capable of generating I is referred to as an *S*-restricted RNG. Simply put, *S*-restricted RNG, which takes an arbitrary set *S* of integers as the input, and randomly generates the elements of *S* without generating any other random number. The notion of *S*-restricted RNG is introduced for the first time in this paper. We use it as part of our solution to the problem of *random object generation*.

#### 1.1.4. Latin Squares

A Latin square of order *q* contains 1,2,…,q in each row and each column in a way that no number is repeated in a row or a column. Latin squares are of many applications in cryptography [15,16,17] and related areas [18]. Table 1 shows a Latin square of order 10.

### 1.2. Organization

The rest of this paper is organized as follows: Section 2 formalizes the problem of *random object generation*, discusses the challenges raised by the problem and presents our proposed solution based on *S*-restricted RNGs and encoding schemes. Section 3 reviews related research works. This section compares the most relevant works with our work in this paper. Section 4 presents a novel method based on integer compositions for designing parallel LFSRs. Section 5 proposes an architecture for designing *S*-restricted RNGs using parallel LFSRs. Section 6 presents the case study. The first subsection in this section presents a novel encoding scheme for Latin squares. The second subsection designs a circuit for generating random Latin squares of order 4. Lastly, Section 7 concludes the paper and suggests further research.

## 2. Preliminaries

In this section, we preset some preliminary discussions. In Section 2.1, we state the problem of *random object generation*. Section 2.2 discusses the challenges raised by the formulated problem. Section 2.3 introduces our approach to solving the problem. Lastly, Section 2.4 explains the novelties and achievements of this paper.

### 2.1. Problem Statement

For n∈N, let $O=\{o_{1},o_{2},\ldots ,o_{n}\}$ and $S=\left\{s_{1},s_{2},\ldots ,s_{n}\right\}\subset Z^{+}$ represent a set of objects and a set of non-negative integers, respectively. Furthermore, let M:O↦S be an injective and surjective map. In the presence of M, we refer to each element of *S* as a *valid object code*, and any other integer is considered as an *invalid object code*. The problem of *random object generation* is defined as the problem of generating a random sequence of positive integers $I:{\left(i_{l}\right)}_{l=1}^{t}$ in a way that $\{i_{l}|i_{l}\in I\}=S$. Solving this problem requires an RNG that generates the sequence I. For n∈N, let $O=\{o_{1},o_{2},\ldots ,o_{n}\}$ and $S=\left\{s_{1},s_{2},\ldots ,s_{n}\right\}\subset Z^{+}$ representing a set of objects and a set of non-negative integers, respectively. Furthermore, let M:O↦S be an injective and surjective one-to-one map, playing the role of an encoding scheme. In the presence of M, we refer to each element of *S* as a *valid object code*, and any other integer is considered as an *invalid object code*. The problem of *random object generation* is defined as the problem of generating a random sequence of positive integers $I:{\left(i_{l}\right)}_{l=1}^{t}$ in a way that $\{i_{l}|i_{l}\in I\}=S$. Solving this problem requires an RNG that generates the sequence I.

### 2.2. Challenges

A naive solution to the problem of *random object generation* is to assign numerical codes to objects, let an RNG generate random numbers and interpret the generated random numbers as object codes. However, there are some challenges. These challenges are discussed below.

1. The first challenge here is to find or to build an RNG for which the set of possible outputs is exactly equal to the set of codes assigned to the objects. For example, for random permutations, we require encoding schemes that assign codes from S={1,2,…,q!} to permutations of *q* objects [19]. Each of the mentioned codes can be represented by l=⌈logq!⌉ binary digits. However, a *l*-bit RNG usually generates all $2^{l}$ elements of $\left\{0,1,\ldots ,2^{l}-1\right\}$, while $2^{l}-q!=2^{\left\lceil \log q!\right\rceil }-2^{\log q!}$ of them are *invalid*. We address this challenge by introducing *S*-restricted RNGs, which generate random numbers drawn from a given set *S*.
2. The second challenge is that the encoding scheme will most likely be different from one problem to another. For example, an encoding scheme proposed for passwords may not be applicable to CAPTCHAs as the statistical properties of valid passwords are totally different from those of valid CAPTCHAs.
3. Third, the *S*-restricted RNG will be dependent on the encoding scheme and consequently on the target set of objects. This component will vary from Latin squares of order *n* to the same squares of order m≠n. We address this challenge as well as the above one via proposing the use of reconfigurable *S*-restricted RNGs. In our case study, this challenge is resolved in two ways. First, our proposed architecture for designing *S*-restricted RNG is capable of adopting any kind of parallel RNG. Second, we use programmable parallel LFSRs instead of fixed-polynomial parallel LFSRs to improve the reconfigurability of the design. Existing methods for designing parallel LFSRs work only with a fixed generating polynomial [20,21]. In addition to inadequate reconfigurability, fixed-polynomial LFSRs make the system more vulnerable against some well-known security attacks [22].

### 2.3. Problem Solving Approach

**Figure 5:**
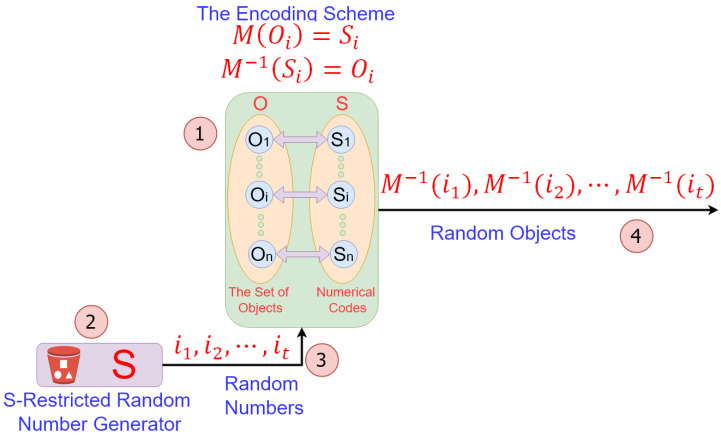
A solution to the problem of *random object generation*. Using *S*-Restricted RNGs: (1) An encoding scheme is created that assigns the set *S* of numeric codes to the set *O* of objects using a one-to-one (reversible) mapping. (2) An *S*-restricted random number generator is designed that is capable of generating elements of *S* in a random way. (3) The *S*-restricted random number generator generates random numbers. (4) The generated random numbers are converted to random objects using the reverse of the encoding map.

Figure 5 shows our solution to the problem of *random object generation*.

The solution illustrated in Figure 5 depends on an encoding scheme and an *S*-restricted RNG. The encoding scheme is inevitably dependent on the specific set of target objects. We compensate this dependence via the use of flexible *S*-restricted RNGs. This way, our method can be used for solving any kind of random non-numerical object generation problem with the help of a proper encoding scheme and an *s*-restricted RNG that generates exactly the set of numeric codes assigned to the objects.

In our proposal, the *S*-restricted RNG is constructed of a few parallel RNGs, which simultaneously generate multiple random numbers, along with a selection mechanism that chooses a *valid* object code among the generated random numbers. As a case study, we will show later how *S*-restricted RNGs can be used to generate Latin squares. We use parallel LFSRs to build parallel RNGs and subsequently *S*-restricted RNGs for generating Latin squares of order 4. We have chosen LFSRs due to their lightweight implementation.

**Figure 6:**
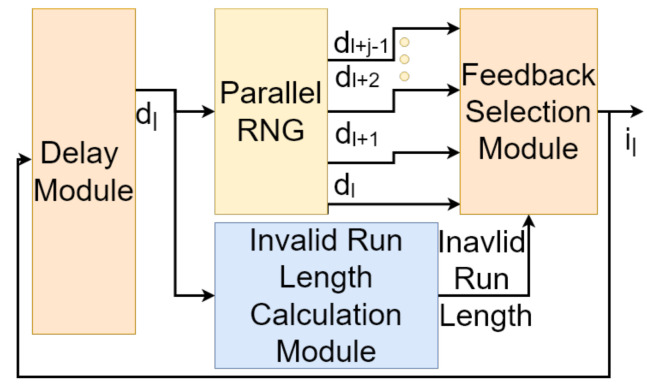
The architecture of an *S*-restricted RNG based on a parallel RNG.

Figure 6 demonstrates our proposed architecture for the design of *S*-restricted RNGs. This architecture is based on parallel RNGs. A parallel RNG is capable of creating multiple simultaneous random numbers at its output [23]. In addition to parallel RNGs, the proposed architecture uses an *Invalid Run length calculation module* as well as a *feedback selection module*. In Figure 6, the delay module is initialized to $d_{l}$. In each clock, the parallel RNG generates $\left\{d_{l},r_{l+1},d_{l+2},\ldots ,d_{l+j-1}\right\}$ where *j* is the sampling rate of the parallel RNG. $r_{i}=r$. An invalid run length equal to *k* allows $i_{l}=d_{l+k}$ in the output and loads it on the feedback loop as well.

### 2.4. Novelties and Contributions

The contributions of this paper can be listed as follows:

1. In this paper, we unify all problems related to random generation of non-numerical entities for the first time. We bring all these problems under a single umbrella via posing and formulating the general problem of *random object generation* (Section 2.1).
2. This paper is the first to propose a solution suitable for generating random instances of any kind of non-numerical entity. Our solution depends on two core components. The first component is a proper encoding scheme assigning a unique code to every individual object. The second component is an RNG capable of generating random numbers restricted to the set of assigned numeric codes (Section 2.3).
3. In this paper, we propose a novel approach based on integer compositions for automatic design of programmable parallel LFSRs (Section 4);
4. In this paper, we introduce the notion of *S*-restricted, RNGs for the first time. Moreover, we present a novel method for designing *S*-restricted RNGs using parallel LFSRs (Section 5);
5. This paper presents the first encoding scheme for Latin squares. This encoding is essentially en extended variant of Lehmer’s code previously proposed for encoding permutations of a set of objects (Section 6.1);
6. We propose the first circuit for generating random Latin squares of degree 4 (Section 6.2).

**Figure 7:**
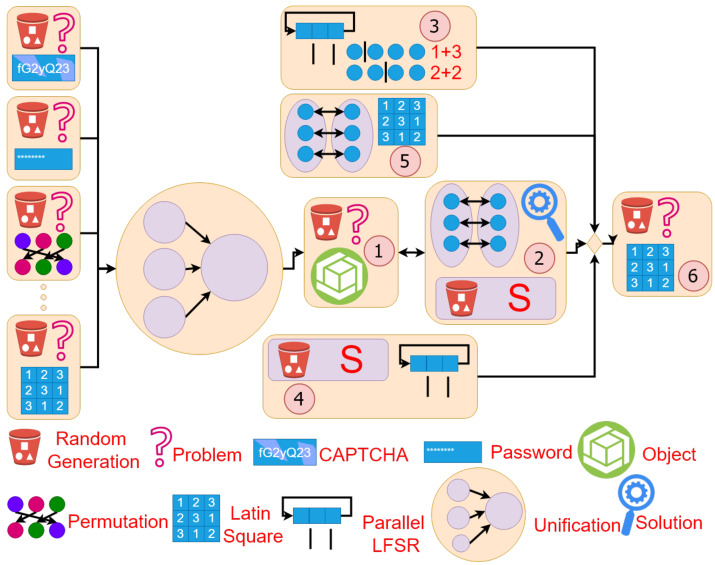
The achievements of this paper: (1) We unify problems like random CAPTCHA generation, random password generation, random permutation generation and random Latin square generation. We formulate the unified problem as the *random object generation* problem. (2) We present a solution based on proper encoding and *S*-restricted random number generators for the problem of *random object generation*. (3) We present an encoding scheme for Latin squares. (4) We propose a method based on integer compositions for designing parallel LFSRs. We propose a method based on parallel LFSRs for designing *S*-restricted random number generators. (6) As a case study, we propose a logic circuit for generating Latin squares of order 4.

Figure 7 shows the achievements of this paper and how they are connected to each other.

## 3. Background and Related Works

In this section, we take a quick look at the background of research on random number generation as well as the random generation of non-numerical entities. We compare the most relevant research works with our work in this paper.

### 3.1. Random Numbers

Random number generation is an old problem. Random bit generation [24], random sequence generation [25], and random vector generation [26] can be mentioned as variants of this problem. There are two main types of random numbers, namely true-random numbers and pseudo-random numbers.

True random numbers are generated using an unpredictable physical object, phenomenon or process referred to as the source of randomness. Among these sources, one may refer to the following:

- Noises [27,28];
- Waves [29,30];
- Hardware Sources [31].

Pseudo-random numbers are completely computer-generated. They are generated using computer algorithms or devices. Pseudo-random numbers are used in a variety of applications ranging from sensor networks [32] to cryptography [33]. Different approaches have been used for designing pseudo-random number generators [34,35]. Moreover, several enabling technologies including artificial intelligence [36,37], fuzzy logic [38] and chaos theory [39] are used for this purpose.

### 3.2. LFSRs and Parallel LFSRs

Among existing random umber generators, LFSRs are most relevant as we are using them in our case study. The research community has considered LFSRs as promising choices for random number generation and cryptographic purposes because of their low area and power consumption as well as their high throughput [40,41]. They have been widely used for both pseudo-random [42] and true-random number [43,44] generation. Different variants of LFSRs have been used for this purpose [22,45,46]. LFSRs are particularly used for random key generation in stream ciphers [47]. In addition to serial LFSRs, parallel LFSRs have been of interest to the research community in recent years [48]. They have been widely used in random number generation [49] and many other applications [50,51].

### 3.3. Random Non-Numerical Entities

Generating random non-numeric entities dates back to the last few decades [52,53,54]. Random network coding [55], random decision trees [56] or random device IDs [57] are required in different domains. Just as an example, random deadlines [58], random power levels [59] and random linear network codes [60] are required to be generated in IoT applications for traffic management, attack resistance and bandwidth management purposes, respectively. Particularly, random permutations [61], random passwords [1], and random CAPTCHAs [2] play significant roles in cryptography.

Different Approaches can be used to generate random non-numeric entities [62,63]. A variety of enabling technologies including chaos theory [64], information theory [65] and artificial intelligence [66] are utilized in this area.

#### Random Latin Squares

Latin squares appear as part of our case study in this paper. This is why we would like to discuss them separately. Random Latin squares have been widely used in IoT environments for channel access arbitration [67], encryption [15], secret sharing [18], etc. Generating random Latin squares is a critical problem in the realm of combinatorial cryptography [6,7,68,69]. Currently, there is no systematic solution for this problem.

### 3.4. Most Relevant Works

According to the above discussions, many existing research reports are somehow relevant to this study. However, the most relevant research works are those focusing on restricted random number generation or generating random non-numerical entities using RNGs. These works are studied in the following:

#### 3.4.1. Restricted RNGs

By a *restricted RNG*, we mean an RNG that generates a certain subset of $\left\{0,1,2,\ldots ,2^{m}-1\right\}$. The literature comes with some RNGs of this type.

As an example, one may refer to *constrained random number generators*, which have been of interest to researchers in recent years [70]. A constrained random number generator is defined as follows [71]. Let *x* be an element of the Cartesian product $\chi^{n}$ of a given set χ. Constrained RNG uses a sequence of random numbers subject to a distribution *v* on $\chi^{n}$ defined by Equation (3):

(3) $$v\left(x\right)=\left\{\begin{array}{cc} \frac{\mu \left(x\right)}{\mu \left(\{x:Ax=c\}\right)}, & Ax=C;\\ 0, & Otherwise. \end{array}\right.$$

In Equation (3), μ is a probability distribution on $\chi^{n}$, *A* is a function $A\;:\;\chi^{n}\to \left\{Ax\;:\;x\in \chi^{n}\right\}$, and $C\in \{Ax\;:\;x\in \chi^{n}\}$. Constrained RNGs are used in channel coding [72].

A constrained RNG is similar to an *S*-restricted RNG in that both of them filter the output of a traditional RNG. However, the difference is that a constrained RNG uses the value of a function to filter the generated random numbers, while an *S*-restricted RNG checks them against a given set *S* of valid numbers.

Another relevant research has been reported in [23], which presents a VLSI (Very Large Scale Integration) design for a parallel RNG, and uses it to generate random numbers drawn from an interval of integers. This work is different from ours in three aspects. First, the parallel RNG designed in [23] generates multiple streams of random numbers, while our proposed *S*-restricted RNG architecture depends on parallel RNGs that generate multiple random numbers from the same stream as their output each time. Second, their designed RNG generates random numbers is a contiguous interval of integers, while our *S*-restricted RNG can generate elements of any arbitrary set *S*. Third, in our work, parallel RNGs are based on parallel LFSRs and can be automatically designed with arbitrary generating polynomials.

#### 3.4.2. RNGs and Random Non-Numerical Entities

Some researchers have proposed methods based on random number generators for generating random decision trees [56], random permutations, and random device IDs [73]. However, none of these methods are capable of being applied on all kinds of non-numerical entities.

### 3.5. Motivations

There are a wide range of problems, each of which can be considered as a spacial instance of the problem posed in this paper: *random object generation*. However, they have not been formulated as a single general problem. The reason is the lack of a general-purpose solution applicable to all of these problems. This is the gap we are addressing in this paper. We formalize the general problem and provide a general solution for it.

## 4. Automatic Design of Parallel LFSRs Using Integer Compositions

Existing methods for designing parallel LFSRs work only with a fixed generating polynomial [20,21]. However, in security-related applications, we are usually interested in randomizing the generating polynomial. Thus, we need an automatic method for designing programmable parallel LFSRs.

In this section, we present a framework for automatic design of parallel LFSRs using the mathematical properties of integer compositions. This framework consists of two parts. In the first part, we present a method for automatic derivation of Reed–Muller expressions describing programmable parallel LFSRs using compositions and palindromes. Afterwards, we modify the framework to describe (non-programmable A.K.A fixed-polynomial) parallel LFSRs by Reed–Muller expressions using *S*-restricted palindromes. In the second part of the framework, we present procedures for automatic generation of compositions, palindromes and *S*-restricted palindromes. For j∈{0,1,…,n−1}, let $b_{j}\left(i\right)$ represent the ${\left(n-j\right)}^{th}$ most significant digit of $B_{n}\left(i\right)=b_{n-1}\left(i\right)b_{n-2}\left(i\right)\ldots b_{j}\left(i\right)\ldots b_{1}\left(i\right)b_{0}\left(i\right)$ being the binary representation of $i\in Z^{+}$ in *n* digits. The Reed–Muller canonical form represents a Boolean function $f(x_{n-1},x_{n-2},\ldots ,x_{1},x_{0})$ in the form of Equation (4),

(4) $$f\left(x_{n-1},x_{n-2},\ldots ,x_{1},x_{0}\right)=\sum_{i=0}^{2^{n}-1}\left(a_{i}\prod_{b_{j}\left(i\right)=1}x_{j}\right).$$

In Equation (4), the addition and multiplication operations are defined over GF(2). A Boolean function described in the Reed–Muller canonical form can be uniquely converted to other canonical forms such as CNF (Conjunctive Normal Form) and DNF (Disjunctive Normal Form). An advantage of Reed–Muller canonical form is that it does not need any NOT gates to be implemented. This canonical form is commonly used in the description and design of logic circuits [74].

In the second part of our framework for designing parallel LFSRs, we present procedures for automatic generation of compositions, palindromes and *S*-restricted palindromes.

### 4.1. Expression Derivation

In this subsection, we take an inductive approach to derive a Boolean expression for describing a programmable parallel LFSR. We begin with Equation (1) and expand it. Induction helps us describe consecutive expansions of this equation using palindromic integer composition.

Before beginning to derive the expressions, let us prove Lemma 1, which presents a procedure for creating C(i+1) (the set of compositions of i+1) given C(i) (the set of compositions of *i*).

**Lemma** **1.** *Procedure CreateNextComp in Algorithm 1 returns $C_{i+1}=C\left(i+1\right)$ provided that $C_{i}=C\left(i\right)$.*

**Algorithm 1** create C(i+1) given C(i)
**Requires:** 
*$C_{i}=C\left(i\right)$.*
**Ensures:** 
*$C_{i+1}=C\left(i+1\right)$.*
1:
**procedure** 
CreateNextComp
*($C_{i}$)*2:*    $C_{i+1}=\emptyset$*3:*    *
**for all** 
*$C\in C_{i}$*
**do**
4:*        $C_{i+1}\leftarrow C_{i+1}\cup \left\{\left(first(C)+1\right);f^{-}\left(C\right)\right\}$*
5:        $C_{i+1}\leftarrow C_{i+1}\cup \left\{1;C\right\}$6:*    *
**end** 
**for**
7:    **return***$C_{i+1}$*

8:
**end** 
**procedure**

**Proof.**  Since $C_{i}=C\left(i\right)$, it is obvious that $\forall C\in C_{i}:\left(first(C)+1\right);f^{-}\left(C\right)\in C\left(i+1\right)\wedge 1;C\in C\left(i+1\right)$ Thus, $C_{i+1}\subset C\left(i+1\right)$. On the other hand, $\forall C^{\prime }\in C\left(i+1\right):(first\left(C^{\prime }\right)=1\wedge f^{-}\left(C^{\prime }\right)$$\in C\left(i\right))\vee \left(first(C^{\prime })-1\right);f^{-}\left(C^{\prime }\right)\in C\left(i\right)$. Thus, $C\left(i+1\right)\subset C_{i+1}$, and the lemma is proved.    □

Equation (5) is immediate from Lemma 1:

(5) $$\forall i>0:C\left(i+1\right)=\left\{\left(first(c)+1\right);f^{-}\left(C\right)|C\in C\left(i\right)\right\}\cup \left\{1;C|C\in C\left(i\right)\right\}$$

Now, let us continue by proving Theorem 1, which gives a Reed–Muller description of $P_{n}^{j}\left(G,M\right)$ using integer compositions.

**Theorem** **1.** *A Reed–Muller expression of $P_{n}^{j}\left(G,M\right)$ (shown in Figure 3) is given by Equation (6), wherein $C^{\prime }=f^{-}\left(C\right)$, $G_{t}=0$ for t≤0 and $F_{t}=M_{-t}$ for t≤0.*

(6) $$\begin{array}{c} \begin{array}{cc} \; & F_{i}\left(j\right)=F_{i-j}\left(0\right)+\\ \; & \sum_{r=0}^{j-1}\sum_{C\in C(j-r)}\left(G_{i-first(C)}\left(\prod_{u=1}^{length(C^{\prime })}G_{n-\lambda \left(C^{\prime },u\right)}\right)F_{n-r}\left(0\right)\right), \end{array} \end{array}$$

*where i∈{1,2,…,n}.*

**Proof.**  We take an induction-based approach to prove Theorem 1. For j=1, considering C(1)={(1)}, first((1))=1 and $length(f^{-}\left(\left(1\right)\right))=0$, Equation (6) is converted to Equation (1). Thus, Equation (6) holds for j=1. On the other hand, if this equation holds for j=q, we can use it to derive Equation (7) via replacing *i* by i−1.

(7) $$\begin{array}{c} \begin{array}{cc} \; & F_{i-1}\left(q\right)=F_{i-(q+1)}\left(0\right)+\\ \; & \sum_{r=0}^{q-1}\sum_{C\in C(q-r)}\left(G_{i-(first(C)+1)}\left(\prod_{u=1}^{length(C^{\prime })}G_{n-\lambda \left(C^{\prime },u\right)}\right)F_{n-r}\left(0\right)\right), \end{array} \end{array}$$

where i∈{2,3,…,n+1}.

In the derivation of Equation (7), we have replaced i−1−q by i−(q+1), and i−1−first(C) by i−(first(c)+1). Furthermore, Equation (8) can be obtained by substituting *n* for *i* in Equation (6) (rewritten for *q* instead of *j*) and multiplying the equation by $G_{i-1}$:

(8) $$\begin{array}{c} \begin{array}{cc} \; & G_{i-1}F_{n}\left(q\right)=G_{i-1}F_{n-q}\left(0\right)+\\ \; & \sum_{r=0}^{q-1}\sum_{C\in C(q-r)}\left(G_{i-1}\left(G_{n-first(C)}\prod_{u=1}^{\mathrm{length}(C^{\prime })}G_{n-\lambda \left(C^{\prime },u\right)}\right)F_{n-r}\left(0\right)\right). \end{array} \end{array}$$

Considering Equations (1) and (5), we can add Equations (7) and (8) together to obtain Equation (9) via some simple algebraic operations:

(9) $$\begin{array}{c} \begin{array}{cc} \; & F_{i}\left(q+1\right)=F_{i-(q+1)}\left(0\right)+\\ \; & \sum_{r=0}^{q}\sum_{C\in C(q+1-r)}\left(G_{i-first(C)}\left(\prod_{u=1}^{length(C^{\prime })}G_{n-\lambda \left(C^{\prime },u\right)}\right)F_{n-r}\left(0\right)\right), \end{array} \end{array}$$

where i∈{1,2,…,n}.

Equation (9) states that, if Equation (6) holds for j=q, it will hold for j=q+1 as well, and the theorem is proved.

   □

It is obvious that $P_{n}^{j}\left(G,M\right)$ can be implemented using logical AND and XOR gates on the basis of the Reed–Muller representation given in Equation (6). Corollary 1 presents an equivalent Reed–Muller description of $P_{n}^{j}\left(G,M\right)$ using palindromes. This can be considered as a simplification to the representation of Theorem 1 because  $|C_{P}\left(x\right)|<|C\left(x\right)|$ for every x>2 and $|C_{P}\left(x\right)|=|C\left(x\right)|$ for every x∈{1,2}.

**Corollary** **1.** *A Reed–Muller expression of $P_{n}^{j}\left(G,M\right)$ (shown in Figure 3) is given by Equation (10), wherein $C^{\prime }=f^{-}\left(C\right)$.*

(10) $$\begin{array}{c} \begin{array}{cc} \; & F_{i}\left(j\right)=F_{i-j}\left(0\right)+\\ \; & \sum_{r=0}^{j-1}\sum_{C\in C_{P}\left(j-r\right)}\left(G_{i-first(C)}\left(\prod_{u=1}^{length(C^{\prime })}G_{n-\lambda \left(C^{\prime },u\right)}\right)F_{n-r}\left(0\right)\right), \end{array} \end{array}$$

*where i∈{1,2,…,n}.*

**Proof.**  In order to prove Corollary 1, we note that

$$\forall C\in C\left(j-r\right):C\notin C_{P}\left(j-r\right)\Rightarrow first\left(C\right);C^{\prime }=first\left(\tilde{C}\right);{\tilde{C}}^{\prime }\Rightarrow$$

$$\begin{array}{c} G_{i-first(C)}\left(\prod_{u=1}^{length(C^{\prime })}G_{n-\lambda \left(C^{\prime },u\right)}\right)+\\ G_{i-first(\tilde{C})}\left(\prod_{u=1}^{length({\tilde{C}}^{\prime })}G_{n-\lambda \left({\tilde{C}}^{\prime },u\right)}\right)=0. \end{array}$$

Thus,

$$\begin{array}{c} \begin{array}{cc} \; & \sum_{C\in C(j-r)}\left(G_{i-first(C)}\left(\prod_{u=1}^{length(C^{\prime })}G_{n-\lambda \left(C^{\prime },u\right)}\right)F_{n-r}\left(0\right)\right)=\\ \; & \sum_{C\in C_{P}\left(j-r\right)}\left(G_{i-first(C)}\left(\prod_{u=1}^{length(C^{\prime })}G_{n-\lambda \left(C^{\prime },u\right)}\right)F_{n-r}\left(0\right)\right). \end{array} \end{array}$$

   □

Given a fixed generating polynomial, $P_{n}^{j}\left(G,M\right)$ can be converted to $L_{n}^{j}\left(G,M\right)$. To show how this can be done, let us prove Corollary 2, which gives a Reed–Muller representation of $L_{n}^{j}\left(G,M\right)$ using *S*-restricted palindromes.

**Corollary** **2.** *A Reed–Muller expression of $L_{n}^{j}\left(G,M\right)$ is given by*

(11) $$\begin{array}{c} \begin{array}{cc} \; & F_{i}\left(j\right)=F_{i-j}\left(0\right)+\\ \; & \sum_{r=0}^{j-1}\sum_{C\in C_{P}^{S(j-r)}\left(j-r\right)}\left(G_{i-first(C)}\left(\prod_{u=1}^{length(C^{\prime })}G_{n-\lambda \left(C^{\prime },u\right)}\right)F_{n-r}\left(0\right)\right), \end{array} \end{array}$$

*where i∈{1,2,…,n} and $C^{\prime }=f^{-}\left(C\right)$.*

**Proof.**  In Equation (10), it is obvious that $\exists i\in C_{P}\left(j-r\right):G_{n-\lambda \left(C^{\prime },u\right)}=0\Rightarrow \prod_{u=1}^{length(C^{\prime })}$$G_{n-\lambda \left(C^{\prime },u\right)}=0$. Thus, given a fixed generating polynomial *G*, the set $C_{P}\left(j-r\right)$ can be replaced with $X\left(j-r,G\right)=\{C|C\in C_{P}\left(j-r\right)\wedge \forall u\in \left\{1,1,\ldots ,length\left(C^{\prime }\right)\right\}:G_{n-\lambda \left(C^{\prime },u\right)}=1\}$. On the other hand, for each $C\in C_{P}\left(j-r\right)$, the set of summands in *C* is equal to that of $C^{\prime }$, except for (j−r). Thus, $X\left(j-r,G\right)=C_{P}^{S(j-r,G)}\left(j-r\right)$, where $S\left(j-r,G\right)=\left\{\left(j-r\right)\right\}\cup \left\{x|G_{n-x}=1\right\}.$    □

### 4.2. Generation Procedures

In this subsection, we complete our automatic parallel LFSR design framework by presenting procedures for generating compositions, palindromes and *S*-restricted palindromes of a positive integer *n*.

Let us begin with systematic generation of C(n). It can be done as shown by procedure *CreateComp* in Algorithm 1 via the use of procedure *CreateComp* in Algorithm 1, which was proved to be correct in Section 4.1.
**Algorithm 2** Create C(n)**Requires:** *n* is a positive integer.**Ensures:** $C_{n}=C\left(n\right)$.1:** procedure**CreateComp(*n*)
2:     **if** n==1 **then**3:         $C_{n}\leftarrow \left\{\left(1\right)\right\}$4:     **else**5:         $C_{n-1}\leftarrow \mathrm{CreateComp}\left(n-1\right)$6:         $C_{n}\leftarrow \mathrm{CreateNextComp}\left(C_{n-1}\right)$7:     **end if**8:     **return**  $C_{n}$9:**end procedure**


Lemma 2 introduces a procedure for systematic creation of $C_{P}\left(n\right)$ for a positive integer *n*.

**Lemma** **2.** *Procedure CreatePal in Algorithm 3 returns $C_{P}\left(n\right)$ for a positive integer n.*

**Algorithm 3** Create $C_{P}\left(n\right)$**Requires:** 
*n* is a positive integer.
**Ensures:** 
*$C_{{}_{p}n}=C\left(n\right)$.*

1: **procedure** 
CreatePal
*(n)*2:*    *
**if** 
*n==1||n==2*
**then**

3:*        $C_{{}_{p}n}\leftarrow \mathrm{CreateComp}\left(n\right)$*
4:    
**else**
5:*        $C_{{}_{p}n}\leftarrow \emptyset$*6: *        $C_{{}_{p}n-2}\leftarrow \mathrm{CreatePal}\left(n-2\right)$*7:         
**for all**
$C\in C_{{}_{p}n-2}$ 
**do**
8: *           $T=\left(first(C)+1\right);f^{-}\left(C\right)$*9: *           $C_{{}_{p}n}\leftarrow C_{{}_{p}n}\cup \left\{l^{-}\left(T\right);\left(last(T)+1\right)\right\}$*10: *           $C_{{}_{p}n}\leftarrow C_{{}_{p}n}\cup \left\{1;C;1\right\}$*11:         
**end** 
**for**
12:     
**end** 
**if**
13:     **return*** $C_{{}_{p}n}$*14:
**end** 
**procedure**

**Proof.**  This lemma can be proved in a very similar way to the case of Lemma 1.    □

Lemma 3 introduces a procedure for generating $C_{P}^{S}\left(n\right)$ for a positive integer *n* and a set *S* of positive integers.

**Lemma** **3.** *For a positive integer n and a set S⊂ of positive integers, procedure CreateSRestPal in Algorithm 4 returns $C_{P}^{S}\left(n\right)$ if $C_{P}^{S}\left(n\right)\neq \emptyset$ and {()} (a set consisting of only an empty composition) if $C_{P}^{S}\left(n\right)=\emptyset$.*

**Algorithm 4** Create S-restricted palindromic compositions**Requires:** 
*n* a positive integer, S a set of positive integers.**     Ensures:** 
*$C_{P}^{S}=C_{P}^{S}\left(n\right)$ if $C_{P}^{S}\left(n\right)\neq \emptyset$, $C_{P}^{S}=\left\{\left(\right)\right\}$ if $C_{P}^{S}\left(n\right)=\emptyset$.*
1:
**procedure** 
CreateSRestPal
*(n,S)*
2: *    G←0*3:     $S_{1}\leftarrow S$4:     
**if** 
*$S_{1}==\emptyset$*
**then**
5:         $C_{P}^{S}\leftarrow \left\{\left(\right)\right\}$        ▹ A set consisting of an empty composition6:         **return**7:     
**end** 
**if**
8:    
**if** 
$n\in S_{1}$ 
**then**
9:         $C_{P}^{S}\leftarrow C_{P}^{S}\cup \left\{\left(n\right)\right\}$10:        G←111:    
**end** 
**if**
12:    $C_{P}^{S}\leftarrow \emptyset$13:    
**for all** 
$s\in S_{1}\setminus \left\{n\right\}$ 
**do**
14:         
**for all** 
$c\in CreateSRestPal(n-2s,S_{1}\setminus \left\{n\right\})$
 
**do**
15:            $c^{\prime }\leftarrow \left(s\right);c;\left(s\right)$        ▹ Concatenate s to the left and the right of *c*16:            $C_{P}^{S}\leftarrow C_{P}^{S}\cup \left\{c^{\prime }\right\}$17:            G←118:         
**end** 
**for**
19:     
**end** 
**for**
20:     
**if** 
G==0 
**then**
21:         $C_{P}^{S}\leftarrow \left\{\left(\right)\right\}$22:    
**end** 
**if**
23:      **return**
24:
**end** 
**procedure**

**Proof.**  In order to prove this lemma, we prove the following statements:

1. $C_{P}^{S}\subset C_{P}^{S}\left(n\right)$ if $C_{P}^{S}\left(n\right)\neq \emptyset$. There are only two statements in Procedure *CreateSRestPa* that add compositions to $C_{P}^{S}$: Statement 9 and Statement 16. Statement 9 adds (n), which is definitely an element of $C_{P}^{S}\left(n\right)$ considering n∈S. Moreover, Statement 16 adds $c^{\prime }=\left(s\right);c;\left(s\right)$, which is an element of $C_{P}^{S}\left(n\right)$ since $c\in C_{P}^{S}\left(n-2s\right)$, and s∈S. Thus, whatever Procedure *CreateSRestPa* adds to *C* is an element of $C_{P}^{S}\left(n\right)$.
2. $C_{P}^{S}\left(n\right)\subset C_{P}^{S}$ if $C_{P}^{S}\left(n\right)\neq \emptyset$. $P_{s}\left(n,S\right)$ may consist of two kinds of compositions.
  - $c_{1}=\left(n\right)$: This composition is added by Statement 9 if n∈S.
  - $\left\{c_{2}|first\left(c_{2}\right)=last\left(c_{2}\right)=s\in S\setminus \left\{n\right\},f^{-}\left(l^{-}\left(c_{2}\right)\right)\in C_{P}^{S\setminus \{n\}}\left(n-2s\right)\right\}$: All of these compositions are added by Statement 16.
  Thus, every element of $C_{P}^{S}\left(n\right)$ is guaranteed to be generated by Procedure *CreateSRestPa*.
  converted to ().
3. $C_{P}^{S}=\left\{\left(\right)\right\}$ if and only if $C_{P}^{S}\left(n\right)=\emptyset$.
  The above two statements show that $C_{P}^{S}=C_{P}^{S}\left(n\right)$. It is immediate that, if $C_{P}^{S}\left(n\right)=\emptyset$, Procedure *CreateSRestPa* will generate no composition. Since 1 is assigned to *G* only after adding compositions to $C_{P}^{S}$ (by Statements 9 and 16), Statement 21 sets $C_{P}^{S}=\left\{\left(\right)\right\}$ if $C_{P}^{S}\left(n\right)=\emptyset$. On the other hand, $C_{P}^{S}$ will not be equal to {()} if $C_{P}^{S}\left(n\right)\neq \emptyset$ because G=1 is executed after generating each composition.

□

## 5. Designing the S-Restricted Random Number Generator

In this section, we propose an architecture for designing *S*-restricted RNGs. The architecture is based on parallel RNGs that generate multiple simultaneous random numbers. While any kind of parallel RNGs can be used in the proposed architecture, we use parallel RNGs constructed of parallel LFSRs to explain the design procedure. The rationale underlying this choice is that LFSRs (and parallel LFSRs) are simple devices with lightweight implementations using a few flip-flops along with a few logical gates, the layout of which is defined by a generating polynomial.

An LFSR $L_{n}\left(G\right)$ of degree *n* with generating polynomial *G* can be used to implement an RNG $R_{n}\left(G\right)$ that periodically generates a sequence of $2^{n}-1$ mutually different values $C_{y}\left(R\right)=r_{0},r_{1},\ldots ,r_{2^{n}-3},r_{2^{n}-2}$ provided that $S^{0}\left(L_{n}\left(G\right)\right)\neq 000\ldots 00$[75]. In this case, each state of $L_{n}\left(G\right)$ is considered as a random number generated by $R_{n}\left(G\right)$. We define $\Delta \left(R_{n}\left(G\right)\right)=\left\{r_{0},r_{1},\ldots ,r_{2^{n}-3},r_{2^{n}-2}\right\}$. Moreover, we define the period length of $R_{n}\left(G\right)$ as the length of the sequence $C_{y}\left(R\right)$, and represent it $|C_{y}\left(R_{n}\left(G\right)\right)|=|\Delta \left(R_{n}\left(G\right)\right)|$.

For a positive integer number *n* and a set $S\subset \{1,2,\ldots ,2^{n-1}\}$, we define an (LFSR-based) *S*-restricted RNG $R_{n}^{S}\left(G\right)$ as an RNG, for which $\Delta (R_{n}^{S}\left(G\right))=S$. We refer to *S* as the set of valid states for $R_{n}^{S}\left(G\right)$. Moreover, we refer to $I\left(R_{n}^{S}\left(G\right)\right))=\left\{1,2,\ldots ,2^{n-1}\right\}\setminus S$ as the set of invalid states of $R_{n}^{S}\left(G\right))$.

**Figure 8:**
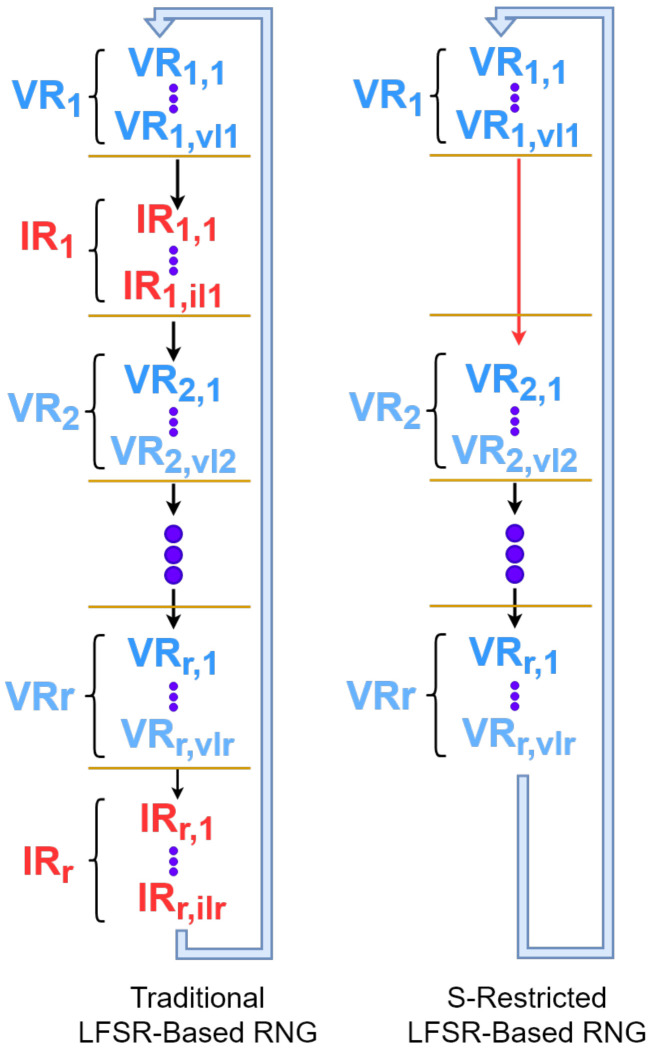
A comparison between traditional and *S*-restricted LFSR-based RNGs.

A traditional LFSR-based RNG $R_{n}\left(G\right)$ is obviously incapable of serving as an *S*-restricted RNG as it will generate some invalid outputs (outputs which are not valid numeric codes assigned to the objects), and $C_{y}\left(R_{n}\left(G\right)\right)$ will consist of alternate runs of valid and invalid numbers (valid runs and invalid runs) unless $S=\{1,2,\ldots ,2^{n}-1\}$. Figure 8 compares $R_{n}\left(G\right)$ with $R_{n}^{S}\left(G\right))$.

In Figure 8, valid runs ($VR_{1},VR_{2},\ldots ,VR_{r}$) and invalid runs ($IR_{1},IR_{2},\ldots ,IR_{r}$) are highlighted by blue and red colors, respectively. In this figure, $vl_{i}$ and $il_{i}$ represent the length of the $i^{th}$ valid run and that of the $i^{th}$ invalid run, respectively. This figure shows how invalid runs are bypassed by $R_{n}^{S}\left(G\right))$.

**Figure 9:**
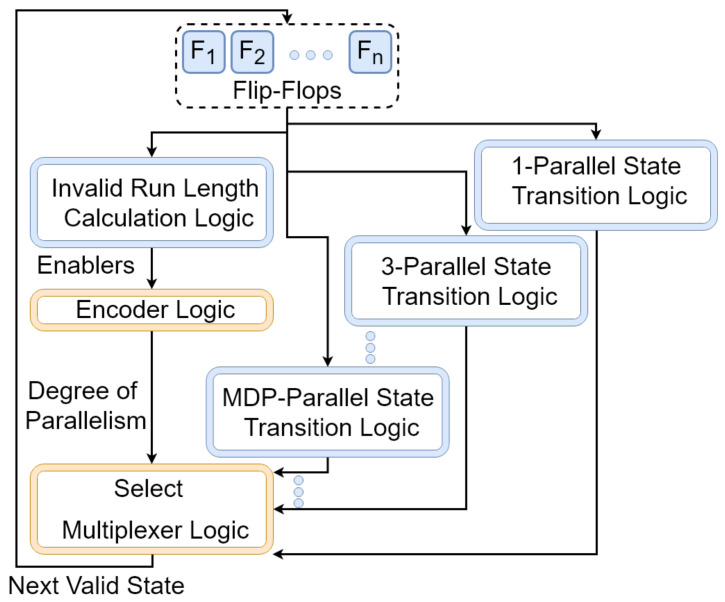
The proposed architecture for *S*-restricted LFSR-based RNG.

A parallel LFSR $L_{n}^{j}\left(G\right)$ with a sampling rate of *j* can bypass j−1 consecutive states generated by $L_{n}^{j}\left(G\right)$ in each clock cycle. Thus, a number of parallel LFSRs with different degrees of parallelism can be used to bypass invalid runs with different lengths. In this section, we use this idea for the design of *S*-restricted RNGs. Figure 9 shows our proposed architecture for the design of $R_{n}^{S}\left(G\right))$.

As shown in Figure 9, the proposed architecture consists of a serial (1-parallel) LFSR ($L_{n}\left(G\right)$) and a set of parallel LFSRs ($L_{n}^{2}\left(G\right)$, $L_{n}^{3}\left(G\right)$,…,$L_{n}^{MIRL}\left(G\right)$), which share *n* flip-flops along with an *Invalid Run Length Calculation Logic* (ILCL) and a multiplexing logic, which control the feedback loop. In this figure, MDP (Maximum Degree of Parallelism) is calculated as MDP=MIRL+1, and MIRL (Maximum Invalid Run Length) is calculated as $MIRL=Max\{il_{1},il_{2},\ldots ,il_{r}\}$. The architecture includes a *j*-parallel state transition logic if and only if $\exists v\in \left\{1,2,\ldots ,r\right\}:il_{v}=j$.

In the architecture of Figure 9, each state transition logic circuit is fed by the outputs of the flip-flops $F_{1},F_{2},\ldots ,F_{n}$ (the current state of the circuit). In each clock cycle, the ILCL generates (at most) MDP enabling signals (enablers), which we denote by $e_{1},e_{2},\ldots ,e_{MDP}$. The signal $e_{1}$ will carry the logic value 1 if the current state of the circuit is a valid state. For i∈{2,3,…,MDP}, the enabler $e_{i}$ will be active if the current state is the beginning of an invalid run of length i−1. The enablers are encoded to the proper degree of parallelism using an encoding logic to provide proper select signals for the multiplexing logic. Finally, we have the multiplexing logic Multiplexes among the outputs of the state transition logic modules to feed the next valid state back into the flip-flops.

$IR_{1,1},IR_{2,1},\ldots ,IR_{r,1}$ can be used to design logical signals $e_{2},e_{3},\ldots ,e_{MDP}$, where $e_{t}$ (if equal to 1) indicates that the RNG should bypass t−1 invalid states. An encoder converts these signals to proper select inputs for the multiplexer.

## 6. Case Study: Random Latin Squares of Order 4

In this section, we first present a method for assigning numerical codes to Latin squares of an arbitrary order *q*. Afterwards, we design an *S*-restricted RNG that generates random valid codes for Latin codes of order 4.

### 6.1. Encoding Latin Squares

A Latin square λ of order *q* can be modeled by $\langle \lambda_{1},\lambda_{2},\ldots ,\lambda_{q}\rangle$, where $\forall i,j\in \left\{1,2,\ldots ,q\right\}:\lambda \left(i\right)=\lambda_{i}\in \Gamma \left(\left\{1,2,\ldots ,q\right\}\right)\wedge \lambda_{i}\in D\left(\lambda_{j}\right)$. Thus, the methods that are used for encoding permutations [19] can be modified to encode Latin squares. In this paper, we modify Lehmer’s encoding scheme [19] to assign numerical codes to Latin squares of degree *q*. For every Ξ⊂{1,2,…,q}, and every ξ∈Ξ, let us define Φ(ξ,Ξ)=|{x|x∈Ξ∧x<ξ}|. Lehmer’s encoding scheme assigns a numerical code 0≤H(γ)≤q!−1 to each γ∈Γ({1,2,…,q}) on the basis of Equation (12),

(12) $$H\left(\gamma \right)=\sum_{i=1}^{q}\left(q-i\right)!H\left(\gamma (i),\{1,2,\ldots ,q\}\setminus \{\gamma (j)|0<j<i\}\right).$$

As an example, consider H(〈3,1,2〉)=2!∗2+1!∗0+0!∗0=4. Now let us define the total order ⩽ on Γ({1,2,…,q}) as $\forall \gamma_{1},\gamma_{2}\in \Gamma \left(\left\{1,2,\ldots ,q\right\}\right):\gamma_{1}⩽\gamma_{2}\Leftrightarrow H\left(\gamma_{1}\right)\leq H\left(\gamma_{2}\right)$. Using the total order ⩽ on Γ({1,2,…,q}), we define ⪯ on $\Lambda_{q}$ as $\forall \lambda_{1},\lambda_{2}\in \Lambda_{q}:\lambda_{1}⪯\lambda_{2}\Leftrightarrow \exists i\in \left\{1,1,\ldots ,q\right\}:\left(\forall \;1\leq j\leq i:\lambda_{1}\left(j\right)=\lambda_{2}\left(j\right)\wedge \lambda_{2}\left(i+1\right)⩽\lambda_{1}\left(i+1\right)\right)$. In our proposed encoding scheme, the numerical code of each $\lambda \in \Lambda_{q}$ is calculated as shown in Equation (13),

(13) $$F\left(\lambda \right)=|\left\{x|x\in \Lambda_{q}\wedge x⪯\lambda \right\}|+1.$$

The codes assigned to Latin squares of order 4 can be seen in Appendix A.

### 6.2. *S*-Restricted RNG for $S=\{F\left(\lambda \right)|\lambda \in \Lambda_{4}\}$

The first step towards the design of an *S*-restricted RNG is to list the set *S* of valid states, and to decide the size of the LFSRs (the number of flip-flops) accordingly. It is well known that $|\Lambda_{q}|=576$. Therefore, using the method explained above, the elements of $\Lambda_{4}$ can be encoded by the elements of S={1,2,…,576}. Moreover, the size of each LFSR should be ⌈log576⌉=10.

The second step is to decide the generating polynomial for the serial and parallel LFSRs. There are 60 different primitive polynomials of degree 10 over GF(2). In this case study, we have chosen $G=x^{10}+x^{3}+1$ because it has the minimum number of non-zero coefficients, and this reduces the number of gates required to implement the state transition logic modules. One can obviously choose polynomials with more non-zero coefficients in order to improve resistance against algebraic attacks.

Now we need to analyze the period of $L_{10}\left(G\right)$ to identify the beginning state and the length of each invalid run to decide the set of sampling rates and design the corresponding state transition logic modules. We have analyzed a whole period of $L_{10}\left(G\right)$ beginning from the initial state 0000000001, and identified 221 invalid runs. These runs are shown in the supplementary file. Runs of lengths 1 through 8 as well as 9 and 10 have been identified in the period. The beginning states of the runs are shown in Table 2 along with the corresponding run lengths. In Table 2, the columns labeled “L” show the run lengths, and the ones labeled “Begin” show the beginning states of the runs.

**Figure 10:**
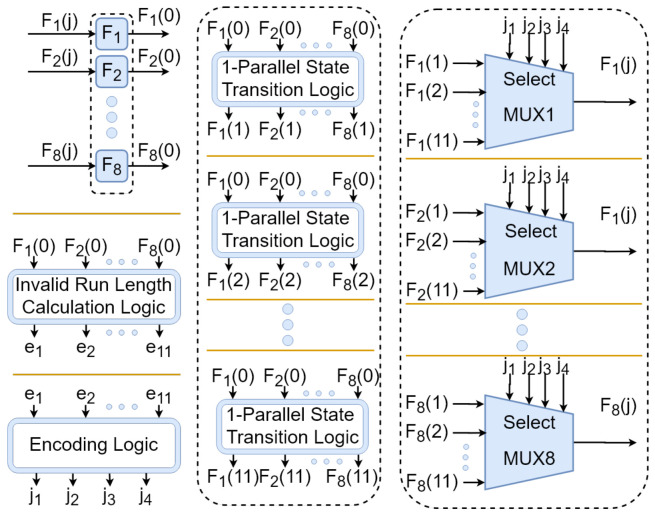
The components of *S*-restricted RNG for generating random Latin squares of order 4.

Now we can design the components of the *S*-restricted RNG in the form of black boxes. These components are shown in Figure 10.

In the next step, the state transition logic should be designed. This can be done using the method presented in Section 4. Table 3 and Table 4 show the logic expressions describing the output of the state transition logic modules.

Now, let us design the ILCL. It can be designed according to the information given in Table 2. As an example, to show how the ILCL is designed, let us consider the row labeled “L = 6” in Table 2. The beginning states in this row can be represented as 10-digit binary numbers by 1010011111, 1011011111, 1101011111 and 1001011111, respectively. This corresponds to the logic expression given by Equation (14) (In the rest of this section, we simply use `$F_{i}$’ instead of `$F_{i}\left(0\right)$’ for i∈{1,2,…,10}).

(14) $$\begin{array}{c} \begin{array}{cc} \; & e_{7}=F_{1}F_{2}^{\prime }F_{3}F_{4}^{\prime }F_{5}^{\prime }F_{6}F_{7}F_{8}F_{9}F_{10}†F_{1}F_{2}^{\prime }F_{3}F_{4}F_{5}^{\prime }F_{6}F_{7}F_{8}F_{9}F_{10}\;†\\ \; & F_{1}F_{2}F_{3}^{\prime }F_{4}F_{5}^{\prime }F_{6}F_{7}F_{8}F_{9}F_{10}†F_{1}F_{2}^{\prime }F_{3}^{\prime }F_{4}F_{5}^{\prime }F_{6}F_{7}F_{8}F_{9}F_{10}. \end{array} \end{array}$$

In Equation (14), † represents logical OR (to separate it from logical XOR or GF(2) addition). The logical expression in Equation (14) can be simplified as shown by Equation (15) using standard logic function simplification methods:

(15) $$e_{7}=F_{1}F_{2}^{\prime }F_{3}F_{5}^{\prime }F_{6}F_{7}F_{8}F_{9}F_{10}†F_{1}F_{3}^{\prime }F_{4}F_{5}^{\prime }F_{6}F_{7}F_{8}F_{9}F_{10}.$$

Non-simplified logic expressions of the enablers are listed in the Supplementary File. Simplified enabler expressions are shown in Table 5.

The invalid runs and the enablers of the circuit designed in this section can be seen in Appendixes Appendix B and Appendix C, respectively.

## 7. Conclusions and Further Works

In this paper, we unified the problems of random password generation, random captcha generation and random permutation generation as well as several similar problems into a generalized problem, which we call *random object generation*. The advantage of this generalization is that every solution proposed for the generalized problem will work for all the aforementioned problems. Moreover, we proposed a solution to the *random object generation* problem via introducing the notion of *S*-restricted RNG, which generates random numbers restricted to be drawn from a given set *S*. Our solution requires *S* to be the set of numeric codes assigned to objects of a specific type. We demonstrated how *S*-restricted RNGs can be constructed using parallel RNGs. We illustrated the construction procedure using parallel LFSRs. Moreover, we examined random generation of Latin squares as a case study. The most critical limitation of our work is the dependence of the coding scheme, and consequently the *S*-restricted RNG, on the set of target objects. This issue can be addressed via further research on encoding schemes for different kinds of non-numerical entities. Moreover, research can be continued by applying other types of parallel RNGs or Non-Linear Feedback Shift Registers (NFSRs) to the construction of *S*-restricted RNGs. Moreover, interested researchers can analyze the impact of the object encoding scheme as well as the LFSR generating polynomials on the performance and complexity of the *S*-restricted RNGs proposed in this paper.

## Figures and Tables

**Table 1:** A sample Latin square of order 10.

1	8	9	10	2	4	6	3	5	7
7	2	8	9	10	3	5	4	6	1
6	1	3	8	9	10	4	5	7	2
5	7	2	4	8	9	10	6	1	3
10	6	1	3	5	8	9	7	2	4
9	10	7	2	4	6	8	1	3	5
8	9	10	1	3	5	7	2	4	6
2	3	4	5	6	7	1	8	9	10
3	4	5	6	7	1	2	10	8	9
4	5	6	7	1	2	3	9	10	8

**Table 2:** Run beginners.

L	Begin										
1	580 608 834 656 596 706 752 686 694 848 602 726 654 632 640 578 720 598 650 738 760 588 836 610 728 630 688 666 742 584 612 832 582 626 732 638 674 744 622 646 854 642 736 700 684 842 658 740 862 680 618 730 758 604 696 702 644 592 594 724 710 600 668 676 704 664 692 648 616 614 850 660 708 590 718 860 698 750 712 620 838 624 716 844 852 714 754 764 856 670 858 662 652 840 628 606 682 746 762 766 672 586 722 756 634 734 690 748 846 636 678										
2	585 749 653 641 577 589 665 757 637 685 645 725 705 621 669 593 733 597 761 717 649 721 845 697 849 693 737 601 661 833 857 765 837 677 753 673 613 841 605 689 625 581 617 709 729 609 701 861 853 681 657 629 741 633 745 713										
3	747 739 859 643 635 651 851 731 587 843 619 627 835 667 715 603 723 755 699 691 763 595 707 683 659 611 579 675										
4	599 615 679 583 663 631 647 695 743 727 711 759 839 855										
L	Begin	L	Begin	L	Begin	L	Begin	L	Begin	L	Begin
5	623 591 655 751 687 719 847	6	671 735 863 607	7	703	8	767	9	-	10	639

**Table 3:** Logic descriptions of the state transition logic modules in the *S*-restricted RNG for generating random Latin squares of order 4 (Part 1).

*j*	Equation
2	$\left\{\begin{array}{c} F_{1}\left(2\right)=F_{8}\left(0\right),F_{2}\left(2\right)=F_{9}\left(0\right),F_{3}\left(2\right)=F_{10}\left(0\right)\\ F_{4}\left(2\right)=F_{1}\left(0\right)+F_{8}\left(0\right),F_{5}\left(2\right)=F_{2}\left(0\right)+F_{9}\left(0\right)\\ F_{6}\left(2\right)=F_{3}\left(0\right),F_{7}\left(2\right)=F_{4}\left(0\right),F_{8}\left(2\right)=F_{5}\left(0\right)\\ F_{9}\left(2\right)=F_{6}\left(0\right),F_{10}\left(2\right)=F_{7}\left(0\right), \end{array}\right.$
3	$\left\{\begin{array}{c} F_{1}\left(3\right)=F_{7}\left(0\right),F_{2}\left(3\right)=F_{8}\left(0\right),F_{3}\left(3\right)=F_{9}\left(0\right)\\ F_{4}\left(3\right)=F_{10}\left(0\right)+F_{7}\left(0\right),F_{5}\left(3\right)=F_{1}\left(0\right)+F_{8}\left(0\right)\\ F_{6}\left(3\right)=F_{2}\left(0\right)+F_{9}\left(0\right),F_{7}\left(3\right)=F_{3}\left(0\right)\\ F_{8}\left(3\right)=F_{4}\left(0\right),F_{9}\left(3\right)=F_{5}\left(0\right),F_{10}\left(3\right)=F_{6}\left(0\right) \end{array}\right.$
4	$\left\{\begin{array}{c} F_{1}\left(4\right)=F_{6}\left(0\right),F_{2}\left(4\right)=F_{7}\left(0\right),F_{3}\left(4\right)=F_{8}\left(0\right)\\ F_{4}\left(4\right)=F_{9}\left(0\right)+F_{6}\left(0\right),F_{5}\left(4\right)=F_{10}\left(0\right)+F_{7}\left(0\right)\\ F_{6}\left(4\right)=F_{1}\left(0\right)+F_{8}\left(0\right),F_{7}\left(4\right)=F_{2}\left(0\right)+F_{9}\left(0\right)\\ F_{8}\left(4\right)=F_{3}\left(0\right),F_{9}\left(4\right)=F_{4}\left(0\right),F_{10}\left(4\right)=F_{5}\left(0\right) \end{array}\right.$
5	$\left\{\begin{array}{c} F_{1}\left(5\right)=F_{5}\left(0\right),F_{2}\left(5\right)=F_{6}\left(0\right),F_{3}\left(5\right)=F_{7}\left(0\right)\\ F_{4}\left(5\right)=F_{8}\left(0\right)+F_{5}\left(0\right),F_{5}\left(5\right)=F_{9}\left(0\right)+F_{6}\left(0\right)\\ F_{6}\left(5\right)=F_{10}\left(0\right)+F_{7}\left(0\right),F_{7}\left(5\right)=F_{1}\left(0\right)+F_{8}\left(0\right)\\ F_{8}\left(5\right)=F_{2}\left(0\right)+F_{9}\left(0\right),F_{9}\left(5\right)=F_{3}\left(0\right),F_{10}\left(5\right)=F_{4}\left(0\right) \end{array}\right.$
6	$\left\{\begin{array}{c} F_{1}\left(6\right)=F_{4}\left(0\right),F_{2}\left(6\right)=F_{5}\left(0\right),F_{3}\left(6\right)=F_{6}\left(0\right)\\ F_{4}\left(6\right)=F_{7}\left(0\right)+F_{4}\left(0\right),F_{5}\left(6\right)=F_{8}\left(0\right)+F_{5}\left(0\right)\\ F_{6}\left(6\right)=F_{9}\left(0\right)+F_{6}\left(0\right),F_{7}\left(6\right)=F_{10}\left(0\right)+F_{7}\left(0\right)\\ F_{8}\left(6\right)=F_{1}\left(0\right)+F_{8}\left(0\right),F_{9}\left(6\right)=F_{2}\left(0\right)+F_{9}\left(0\right)\\ F_{10}\left(6\right)=F_{3}\left(0\right) \end{array}\right.$
7	$\left\{\begin{array}{c} F_{1}\left(7\right)=F_{3}\left(0\right),F_{2}\left(7\right)=F_{4}\left(0\right),F_{3}\left(7\right)=F_{5}\left(0\right)\\ F_{4}\left(7\right)=F_{6}\left(0\right)+F_{3}\left(0\right),F_{5}\left(7\right)=F_{7}\left(0\right)+F_{4}\left(0\right)\\ F_{6}\left(7\right)=F_{8}\left(0\right)+F_{5}\left(0\right),F_{7}\left(7\right)=F_{9}\left(0\right)+F_{6}\left(0\right)\\ F_{8}\left(7\right)=F_{10}\left(0\right)+F_{7}\left(0\right),F_{9}\left(7\right)=F_{1}\left(0\right)+F_{8}\left(0\right)\\ F_{10}\left(7\right)=F_{2}\left(0\right)+F_{9}\left(0\right) \end{array}\right.$

**Table 4:** Logic descriptions of the state transition logic modules in the *S*-restricted RNG for generating random Latin squares of order 4 (Part 2).

*j*	Equation
8	$\left\{\begin{array}{c} F_{1}\left(8\right)=F_{2}\left(0\right)+F_{9}\left(0\right),F_{2}\left(8\right)=F_{3}\left(0\right),F_{3}\left(8\right)=F_{4}\left(0\right),\\ F_{4}\left(8\right)=F_{5}\left(0\right)+F_{2}\left(0\right)+F_{9}\left(0\right),\\ F_{5}\left(8\right)=F_{6}\left(0\right)+F_{3}\left(0\right),F_{6}\left(8\right)=F_{7}\left(0\right)+F_{4}\left(0\right),\\ F_{7}\left(8\right)=F_{8}\left(0\right)+F_{5}\left(0\right),F_{8}\left(8\right)=F_{9}\left(0\right)+F_{6}\left(0\right),\\ F_{9}\left(8\right)=F_{10}\left(0\right)+F_{7}\left(0\right),F_{10}\left(8\right)=F_{1}\left(0\right)+F_{8}\left(0\right) \end{array}\right.$
9	$\left\{\begin{array}{c} F_{1}\left(9\right)=F_{1}\left(0\right)+F_{8}\left(0\right),\\ F_{2}\left(9\right)=F_{2}\left(0\right)+F_{9}\left(0\right),F_{3}\left(9\right)=F_{3}\left(0\right),\\ F_{4}\left(9\right)=F_{4}\left(0\right)+F_{1}\left(0\right)+F_{8}\left(0\right),\\ F_{5}\left(9\right)=F_{5}\left(0\right)+F_{2}\left(0\right)+F_{9}\left(0\right),\\ F_{6}\left(9\right)=F_{6}\left(0\right)+F_{3}\left(0\right),F_{7}\left(9\right)=F_{7}\left(0\right)+F_{4}\left(0\right),\\ F_{8}\left(9\right)=F_{8}\left(0\right)+F_{5}\left(0\right),\\ F_{9}\left(9\right)=F_{9}\left(0\right)+F_{6}\left(0\right)F_{10}\left(9\right)=F_{10}\left(0\right)+F_{7}\left(0\right) \end{array}\right.$
10	$\left\{\begin{array}{c} F_{1}\left(9\right)=F_{1}\left(0\right)+F_{8}\left(0\right),F_{2}\left(9\right)=F_{2}\left(0\right)+F_{9}\left(0\right),\\ F_{3}\left(9\right)=F_{3}\left(0\right),F_{4}\left(9\right)=F_{4}\left(0\right)+F_{1}\left(0\right)+F_{8}\left(0\right),\\ F_{5}\left(9\right)=F_{5}\left(0\right)+F_{2}\left(0\right)+F_{9}\left(0\right),\\ F_{6}\left(9\right)=F_{6}\left(0\right)+F_{3}\left(0\right),F_{7}\left(9\right)=F_{7}\left(0\right)+F_{4}\left(0\right),\\ F_{8}\left(9\right)=F_{8}\left(0\right)+F_{5}\left(0\right),\\ F_{9}\left(9\right)=F_{9}\left(0\right)+F_{6}\left(0\right)F_{10}\left(9\right)=F_{10}\left(0\right)+F_{7}\left(0\right) \end{array}\right.$
11	$\left\{\begin{array}{c} F_{1}\left(11\right)=F_{9}\left(0\right)+F_{6}\left(0\right),F_{2}\left(11\right)=F_{10}\left(0\right)+F_{7}\left(0\right),\\ F_{3}\left(11\right)=F_{1}\left(0\right)+F_{8}\left(0\right),\\ F_{4}\left(11\right)=F_{2}\left(0\right)+F_{9}\left(0\right)+F_{9}\left(0\right)+F_{6}\left(0\right),\\ F_{5}\left(11\right)=F_{3}\left(0\right)+F_{10}\left(0\right)+F_{7}\left(0\right),\\ F_{6}\left(11\right)=F_{4}\left(0\right)+F_{1}\left(0\right)+F_{8}\left(0\right),\\ F_{7}\left(11\right)=F_{5}\left(0\right)+F_{2}\left(0\right)+F_{9}\left(0\right)F_{8}\left(11\right)=F_{6}\left(0\right)+F_{3}\left(0\right),\\ F_{9}\left(11\right)=F_{7}\left(0\right)+F_{4}\left(0\right),F_{10}\left(11\right)=F_{8}\left(0\right)+F_{5}\left(0\right) \end{array}\right.$

**Table 5:** Simplified logic expressions for enablers in the *S*-restricted RNG for generating random Latin squares of order 4.

*j*	Simplified Logic Expression
2	$e_{2}=F_{1}F_{2}^{\prime }F_{3}F_{10}^{\prime }†F_{1}F_{2}^{\prime }F_{4}F_{5}F_{10}^{\prime }†F_{1}F_{2}F_{3}^{\prime }F_{4}F_{5}^{\prime }F_{10}^{\prime }†F_{1}F_{3}^{\prime }F_{4}F_{5}^{\prime }F_{6}F_{10}^{\prime }†F_{1}F_{3}^{\prime }F_{4}F_{5}^{\prime }F_{7}F_{10}^{\prime }†F_{1}F_{3}^{\prime }F_{4}F_{5}^{\prime }F_{8}F_{10}^{\prime }†F_{1}F_{3}^{\prime }F_{4}F_{5}^{\prime }F_{9}F_{10}^{\prime }$
3	$e_{3}=F_{1}F_{2}^{\prime }F_{3}F_{9}^{\prime }F_{10}†F_{1}F_{2}^{\prime }F_{4}F_{9}^{\prime }F_{10}†F_{1}F_{3}^{\prime }F_{4}F_{5}^{\prime }F_{9}^{\prime }F_{10}$
4	$e_{4}=F_{1}F_{2}^{\prime }F_{3}F_{8}^{\prime }F_{9}F_{10}†F_{1}F_{2}^{\prime }F_{4}F_{8}^{\prime }F_{9}F_{10}†F_{1}F_{3}^{\prime }F_{4}F_{5}^{\prime }F_{8}^{\prime }F_{9}F_{10}$
5	$e_{5}=F_{1}F_{2}^{\prime }F_{3}F_{7}^{\prime }F_{8}F_{9}F_{10}†F_{1}F_{2}^{\prime }F_{4}F_{7}^{\prime }F_{8}F_{9}F_{10}†F_{1}F_{3}^{\prime }F_{4}F_{5}^{\prime }F_{7}^{\prime }F_{8}F_{9}F_{10}$
6	$e_{6}=F_{1}F_{2}^{\prime }F_{3}F_{6}^{\prime }F_{7}F_{8}F_{9}F_{10}†F_{1}F_{2}^{\prime }F_{4}F_{6}^{\prime }F_{7}F_{8}F_{9}F_{10}†F_{1}F_{3}^{\prime }F_{4}F_{5}^{\prime }F_{6}^{\prime }F_{7}F_{8}F_{9}F_{10}$
7	$e_{7}=F_{1}F_{2}^{\prime }F_{3}F_{5}^{\prime }F_{6}F_{7}F_{8}F_{9}F_{10}†F_{1}F_{3}^{\prime }F_{4}F_{5}^{\prime }F_{6}F_{7}F_{8}F_{9}F_{10}$
8	$e_{8}=F_{1}F_{2}^{\prime }F_{3}F_{4}^{\prime }F_{5}F_{6}F_{7}F_{8}F_{9}F_{10}$
9	$e_{9}=F_{1}F_{2}^{\prime }F_{3}F_{4}F_{5}F_{6}F_{7}F_{8}F_{9}F_{10}$
11	$e_{11}=F_{1}F_{2}^{\prime }F_{3}^{\prime }F_{4}F_{5}F_{6}F_{7}F_{8}F_{9}F_{10}$
1	$e_{1}={\left(e_{2}†e_{3}†e_{4}†e_{5}†e_{6}†e_{7}†e_{8}†e_{9}†e_{11}\right)}^{\prime }$

## Data Availability

Not applicable.

## Abbreviations

LFSR	Linear Feedback Shift Register
Completely Automated Public	CAPTCHA generation
Turing test to tell Computers and Humans Apart	
RNG	Random Number Generator
VLSI	Very Large Scale Integration
CNF	Conjunctive Normal Form
DNF	Disjunctive Normal Form
ILCL	Invalid Run Length Calculation Logic
MDP	Maximum Degree of Parallelism
MIRL	Maximum Invalid Run Length
NFSR	Nonlinear Feedback Shift Register

## Appendix A. Codes Assigned to Latin Squares of Order 4

Codes are separated from the corresponding Latin squares using “:”. Each Latin square is represented as a permutation of permutations. Each permutation is denoted using a pair of “〈” and “〉”. Elements of each permutation are separated using “,”.

1: 〈〈1,2,3,4〉,〈2,1,4,3〉,〈3,4,1,2〉,〈4,3,2,1〉〉, 2: 〈〈1,2,3,4〉,〈2,1,4,3〉,〈3,4,2,1〉,〈4,3,1,2〉〉
3: 〈〈1,2,3,4〉,〈2,1,4,3〉,〈4,3,1,2〉,〈3,4,2,1〉〉, 4: 〈〈1,2,3,4〉,〈2,1,4,3〉,〈4,3,2,1〉,〈3,4,1,2〉〉
5: 〈〈1,2,3,4〉,〈3,1,4,2〉,〈2,4,1,3〉,〈4,3,2,1〉〉, 6: 〈〈1,2,3,4〉,〈3,1,4,2〉,〈4,3,2,1〉,〈2,4,1,3〉〉
7: 〈〈1,2,3,4〉,〈4,1,2,3〉,〈2,3,4,1〉,〈3,4,1,2〉〉, 8: 〈〈1,2,3,4〉,〈4,1,2,3〉,〈3,4,1,2〉,〈2,3,4,1〉〉
9: 〈〈1,2,3,4〉,〈2,3,4,1〉,〈4,1,2,3〉,〈3,4,1,2〉〉, 10: 〈〈1,2,3,4〉,〈2,3,4,1〉,〈3,4,1,2〉,〈4,1,2,3〉〉
11: 〈〈1,2,3,4〉,〈2,4,1,3〉,〈3,1,4,2〉,〈4,3,2,1〉〉, 12: 〈〈1,2,3,4〉,〈2,4,1,3〉,〈4,3,2,1〉,〈3,1,4,2〉〉
13: 〈〈1,2,3,4〉,〈3,4,1,2〉,〈2,1,4,3〉,〈4,3,2,1〉〉, 14: 〈〈1,2,3,4〉,〈3,4,1,2〉,〈4,1,2,3〉,〈2,3,4,1〉〉
15: 〈〈1,2,3,4〉,〈3,4,1,2〉,〈2,3,4,1〉,〈4,1,2,3〉〉, 16: 〈〈1,2,3,4〉,〈3,4,1,2〉,〈4,3,2,1〉,〈2,1,4,3〉〉
17: 〈〈1,2,3,4〉,〈3,4,2,1〉,〈2,1,4,3〉,〈4,3,1,2〉〉, 18: 〈〈1,2,3,4〉,〈3,4,2,1〉,〈4,3,1,2〉,〈2,1,4,3〉〉
19: 〈〈1,2,3,4〉,〈4,3,1,2〉,〈2,1,4,3〉,〈3,4,2,1〉〉, 20: 〈〈1,2,3,4〉,〈4,3,1,2〉,〈3,4,2,1〉,〈2,1,4,3〉〉
21: 〈〈1,2,3,4〉,〈4,3,2,1〉,〈2,1,4,3〉,〈3,4,1,2〉〉, 22: 〈〈1,2,3,4〉,〈4,3,2,1〉,〈3,1,4,2〉,〈2,4,1,3〉〉
23: 〈〈1,2,3,4〉,〈4,3,2,1〉,〈2,4,1,3〉,〈3,1,4,2〉〉, 24: 〈〈1,2,3,4〉,〈4,3,2,1〉,〈3,4,1,2〉,〈2,1,4,3〉〉
25: 〈〈1,2,4,3〉,〈2,1,3,4〉,〈3,4,1,2〉,〈4,3,2,1〉〉, 26: 〈〈1,2,4,3〉,〈2,1,3,4〉,〈3,4,2,1〉,〈4,3,1,2〉〉
27: 〈〈1,2,4,3〉,〈2,1,3,4〉,〈4,3,1,2〉,〈3,4,2,1〉〉, 28: 〈〈1,2,4,3〉,〈2,1,3,4〉,〈4,3,2,1〉,〈3,4,1,2〉〉
29: 〈〈1,2,4,3〉,〈3,1,2,4〉,〈2,4,3,1〉,〈4,3,1,2〉〉, 30: 〈〈1,2,4,3〉,〈3,1,2,4〉,〈4,3,1,2〉,〈2,4,3,1〉〉
31: 〈〈1,2,4,3〉,〈4,1,3,2〉,〈2,3,1,4〉,〈3,4,2,1〉〉, 32: 〈〈1,2,4,3〉,〈4,1,3,2〉,〈3,4,2,1〉,〈2,3,1,4〉〉
33: 〈〈1,2,4,3〉,〈2,3,1,4〉,〈4,1,3,2〉,〈3,4,2,1〉〉, 34: 〈〈1,2,4,3〉,〈2,3,1,4〉,〈3,4,2,1〉,〈4,1,3,2〉〉
35: 〈〈1,2,4,3〉,〈2,4,3,1〉,〈3,1,2,4〉,〈4,3,1,2〉〉, 36: 〈〈1,2,4,3〉,〈2,4,3,1〉,〈4,3,1,2〉,〈3,1,2,4〉〉
37: 〈〈1,2,4,3〉,〈3,4,1,2〉,〈2,1,3,4〉,〈4,3,2,1〉〉, 38: 〈〈1,2,4,3〉,〈3,4,1,2〉,〈4,3,2,1〉,〈2,1,3,4〉〉
39: 〈〈1,2,4,3〉,〈3,4,2,1〉,〈2,1,3,4〉,〈4,3,1,2〉〉, 40: 〈〈1,2,4,3〉,〈3,4,2,1〉,〈4,1,3,2〉,〈2,3,1,4〉〉
41: 〈〈1,2,4,3〉,〈3,4,2,1〉,〈2,3,1,4〉,〈4,1,3,2〉〉, 42: 〈〈1,2,4,3〉,〈3,4,2,1〉,〈4,3,1,2〉,〈2,1,3,4〉〉
43: 〈〈1,2,4,3〉,〈4,3,1,2〉,〈2,1,3,4〉,〈3,4,2,1〉〉, 44: 〈〈1,2,4,3〉,〈4,3,1,2〉,〈3,1,2,4〉,〈2,4,3,1〉〉
45: 〈〈1,2,4,3〉,〈4,3,1,2〉,〈2,4,3,1〉,〈3,1,2,4〉〉, 46: 〈〈1,2,4,3〉,〈4,3,1,2〉,〈3,4,2,1〉,〈2,1,3,4〉〉
47: 〈〈1,2,4,3〉,〈4,3,2,1〉,〈2,1,3,4〉,〈3,4,1,2〉〉, 48: 〈〈1,2,4,3〉,〈4,3,2,1〉,〈3,4,1,2〉,〈2,1,3,4〉〉
49: 〈〈2,1,3,4〉,〈1,2,4,3〉,〈3,4,1,2〉,〈4,3,2,1〉〉, 50: 〈〈2,1,3,4〉,〈1,2,4,3〉,〈3,4,2,1〉,〈4,3,1,2〉〉
51: 〈〈2,1,3,4〉,〈1,2,4,3〉,〈4,3,1,2〉,〈3,4,2,1〉〉, 52: 〈〈2,1,3,4〉,〈1,2,4,3〉,〈4,3,2,1〉,〈3,4,1,2〉〉
53: 〈〈2,1,3,4〉,〈1,3,4,2〉,〈4,2,1,3〉,〈3,4,2,1〉〉, 54: 〈〈2,1,3,4〉,〈1,3,4,2〉,〈3,4,2,1〉,〈4,2,1,3〉〉
55: 〈〈2,1,3,4〉,〈1,4,2,3〉,〈3,2,4,1〉,〈4,3,1,2〉〉, 56: 〈〈2,1,3,4〉,〈1,4,2,3〉,〈4,3,1,2〉,〈3,2,4,1〉〉
57: 〈〈2,1,3,4〉,〈3,2,4,1〉,〈1,4,2,3〉,〈4,3,1,2〉〉, 58: 〈〈2,1,3,4〉,〈3,2,4,1〉,〈4,3,1,2〉,〈1,4,2,3〉〉
59: 〈〈2,1,3,4〉,〈4,2,1,3〉,〈1,3,4,2〉,〈3,4,2,1〉〉, 60: 〈〈2,1,3,4〉,〈4,2,1,3〉,〈3,4,2,1〉,〈1,3,4,2〉〉
61: 〈〈2,1,3,4〉,〈3,4,1,2〉,〈1,2,4,3〉,〈4,3,2,1〉〉, 62: 〈〈2,1,3,4〉,〈3,4,1,2〉,〈4,3,2,1〉,〈1,2,4,3〉〉
63: 〈〈2,1,3,4〉,〈3,4,2,1〉,〈1,2,4,3〉,〈4,3,1,2〉〉, 64: 〈〈2,1,3,4〉,〈3,4,2,1〉,〈1,3,4,2〉,〈4,2,1,3〉〉
65: 〈〈2,1,3,4〉,〈3,4,2,1〉,〈4,2,1,3〉,〈1,3,4,2〉〉, 66: 〈〈2,1,3,4〉,〈3,4,2,1〉,〈4,3,1,2〉,〈1,2,4,3〉〉
67: 〈〈2,1,3,4〉,〈4,3,1,2〉,〈1,2,4,3〉,〈3,4,2,1〉〉, 68: 〈〈2,1,3,4〉,〈4,3,1,2〉,〈1,4,2,3〉,〈3,2,4,1〉〉
69: 〈〈2,1,3,4〉,〈4,3,1,2〉,〈3,2,4,1〉,〈1,4,2,3〉〉, 70: 〈〈2,1,3,4〉,〈4,3,1,2〉,〈3,4,2,1〉,〈1,2,4,3〉〉
71: 〈〈2,1,3,4〉,〈4,3,2,1〉,〈1,2,4,3〉,〈3,4,1,2〉〉, 72: 〈〈2,1,3,4〉,〈4,3,2,1〉,〈3,4,1,2〉,〈1,2,4,3〉〉
73: 〈〈2,1,4,3〉,〈1,2,3,4〉,〈3,4,1,2〉,〈4,3,2,1〉〉, 74: 〈〈2,1,4,3〉,〈1,2,3,4〉,〈3,4,2,1〉,〈4,3,1,2〉〉
75: 〈〈2,1,4,3〉,〈1,2,3,4〉,〈4,3,1,2〉,〈3,4,2,1〉〉, 76: 〈〈2,1,4,3〉,〈1,2,3,4〉,〈4,3,2,1〉,〈3,4,1,2〉〉
77: 〈〈2,1,4,3〉,〈1,3,2,4〉,〈4,2,3,1〉,〈3,4,1,2〉〉, 78: 〈〈2,1,4,3〉,〈1,3,2,4〉,〈3,4,1,2〉,〈4,2,3,1〉〉
79: 〈〈2,1,4,3〉,〈1,4,3,2〉,〈3,2,1,4〉,〈4,3,2,1〉〉, 80: 〈〈2,1,4,3〉,〈1,4,3,2〉,〈4,3,2,1〉,〈3,2,1,4〉〉
81: 〈〈2,1,4,3〉,〈3,2,1,4〉,〈1,4,3,2〉,〈4,3,2,1〉〉, 82: 〈〈2,1,4,3〉,〈3,2,1,4〉,〈4,3,2,1〉,〈1,4,3,2〉〉
83: 〈〈2,1,4,3〉,〈4,2,3,1〉,〈1,3,2,4〉,〈3,4,1,2〉〉, 84: 〈〈2,1,4,3〉,〈4,2,3,1〉,〈3,4,1,2〉,〈1,3,2,4〉〉
85: 〈〈2,1,4,3〉,〈3,4,1,2〉,〈1,2,3,4〉,〈4,3,2,1〉〉, 86: 〈〈2,1,4,3〉,〈3,4,1,2〉,〈1,3,2,4〉,〈4,2,3,1〉〉
87: 〈〈2,1,4,3〉,〈3,4,1,2〉,〈4,2,3,1〉,〈1,3,2,4〉〉, 88: 〈〈2,1,4,3〉,〈3,4,1,2〉,〈4,3,2,1〉,〈1,2,3,4〉〉
89: 〈〈2,1,4,3〉,〈3,4,2,1〉,〈1,2,3,4〉,〈4,3,1,2〉〉, 90: 〈〈2,1,4,3〉,〈3,4,2,1〉,〈4,3,1,2〉,〈1,2,3,4〉〉
91: 〈〈2,1,4,3〉,〈4,3,1,2〉,〈1,2,3,4〉,〈3,4,2,1〉〉, 92: 〈〈2,1,4,3〉,〈4,3,1,2〉,〈3,4,2,1〉,〈1,2,3,4〉〉
93: 〈〈2,1,4,3〉,〈4,3,2,1〉,〈1,2,3,4〉,〈3,4,1,2〉〉, 94: 〈〈2,1,4,3〉,〈4,3,2,1〉,〈1,4,3,2〉,〈3,2,1,4〉〉
95: 〈〈2,1,4,3〉,〈4,3,2,1〉,〈3,2,1,4〉,〈1,4,3,2〉〉, 96: 〈〈2,1,4,3〉,〈4,3,2,1〉,〈3,4,1,2〉,〈1,2,3,4〉〉
97: 〈〈1,3,2,4〉,〈2,1,4,3〉,〈4,2,3,1〉,〈3,4,1,2〉〉, 98: 〈〈1,3,2,4〉,〈2,1,4,3〉,〈3,4,1,2〉,〈4,2,3,1〉〉
99: 〈〈1,3,2,4〉,〈3,1,4,2〉,〈2,4,1,3〉,〈4,2,3,1〉〉, 100: 〈〈1,3,2,4〉,〈3,1,4,2〉,〈2,4,3,1〉,〈4,2,1,3〉〉
101: 〈〈1,3,2,4〉,〈3,1,4,2〉,〈4,2,1,3〉,〈2,4,3,1〉〉, 102: 〈〈1,3,2,4〉,〈3,1,4,2〉,〈4,2,3,1〉,〈2,4,1,3〉〉
103: 〈〈1,3,2,4〉,〈4,1,3,2〉,〈3,2,4,1〉,〈2,4,1,3〉〉, 104: 〈〈1,3,2,4〉,〈4,1,3,2〉,〈2,4,1,3〉,〈3,2,4,1〉〉
105: 〈〈1,3,2,4〉,〈3,2,4,1〉,〈4,1,3,2〉,〈2,4,1,3〉〉, 106: 〈〈1,3,2,4〉,〈3,2,4,1〉,〈2,4,1,3〉,〈4,1,3,2〉〉
107: 〈〈1,3,2,4〉,〈2,4,1,3〉,〈3,1,4,2〉,〈4,2,3,1〉〉, 108: 〈〈1,3,2,4〉,〈2,4,1,3〉,〈4,1,3,2〉,〈3,2,4,1〉〉
109: 〈〈1,3,2,4〉,〈2,4,1,3〉,〈3,2,4,1〉,〈4,1,3,2〉〉, 110: 〈〈1,3,2,4〉,〈2,4,1,3〉,〈4,2,3,1〉,〈3,1,4,2〉〉
111: 〈〈1,3,2,4〉,〈2,4,3,1〉,〈3,1,4,2〉,〈4,2,1,3〉〉, 112: 〈〈1,3,2,4〉,〈2,4,3,1〉,〈4,2,1,3〉,〈3,1,4,2〉〉
113: 〈〈1,3,2,4〉,〈4,2,1,3〉,〈3,1,4,2〉,〈2,4,3,1〉〉, 114: 〈〈1,3,2,4〉,〈4,2,1,3〉,〈2,4,3,1〉,〈3,1,4,2〉〉
115: 〈〈1,3,2,4〉,〈4,2,3,1〉,〈2,1,4,3〉,〈3,4,1,2〉〉, 116: 〈〈1,3,2,4〉,〈4,2,3,1〉,〈3,1,4,2〉,〈2,4,1,3〉〉
117: 〈〈1,3,2,4〉,〈4,2,3,1〉,〈2,4,1,3〉,〈3,1,4,2〉〉, 118: 〈〈1,3,2,4〉,〈4,2,3,1〉,〈3,4,1,2〉,〈2,1,4,3〉〉
119: 〈〈1,3,2,4〉,〈3,4,1,2〉,〈2,1,4,3〉,〈4,2,3,1〉〉, 120: 〈〈1,3,2,4〉,〈3,4,1,2〉,〈4,2,3,1〉,〈2,1,4,3〉〉
121: 〈〈1,3,4,2〉,〈2,1,3,4〉,〈4,2,1,3〉,〈3,4,2,1〉〉, 122: 〈〈1,3,4,2〉,〈2,1,3,4〉,〈3,4,2,1〉,〈4,2,1,3〉〉
123: 〈〈1,3,4,2〉,〈3,1,2,4〉,〈2,4,1,3〉,〈4,2,3,1〉〉, 124: 〈〈1,3,4,2〉,〈3,1,2,4〉,〈2,4,3,1〉,〈4,2,1,3〉〉
125: 〈〈1,3,4,2〉,〈3,1,2,4〉,〈4,2,1,3〉,〈2,4,3,1〉〉, 126: 〈〈1,3,4,2〉,〈3,1,2,4〉,〈4,2,3,1〉,〈2,4,1,3〉〉
127: 〈〈1,3,4,2〉,〈4,1,2,3〉,〈3,2,1,4〉,〈2,4,3,1〉〉, 128: 〈〈1,3,4,2〉,〈4,1,2,3〉,〈2,4,3,1〉,〈3,2,1,4〉〉
129: 〈〈1,3,4,2〉,〈3,2,1,4〉,〈4,1,2,3〉,〈2,4,3,1〉〉, 130: 〈〈1,3,4,2〉,〈3,2,1,4〉,〈2,4,3,1〉,〈4,1,2,3〉〉
131: 〈〈1,3,4,2〉,〈2,4,1,3〉,〈3,1,2,4〉,〈4,2,3,1〉〉, 132: 〈〈1,3,4,2〉,〈2,4,1,3〉,〈4,2,3,1〉,〈3,1,2,4〉〉
133: 〈〈1,3,4,2〉,〈2,4,3,1〉,〈3,1,2,4〉,〈4,2,1,3〉〉, 134: 〈〈1,3,4,2〉,〈2,4,3,1〉,〈4,1,2,3〉,〈3,2,1,4〉〉
135: 〈〈1,3,4,2〉,〈2,4,3,1〉,〈3,2,1,4〉,〈4,1,2,3〉〉, 136: 〈〈1,3,4,2〉,〈2,4,3,1〉,〈4,2,1,3〉,〈3,1,2,4〉〉
137: 〈〈1,3,4,2〉,〈4,2,1,3〉,〈2,1,3,4〉,〈3,4,2,1〉〉, 138: 〈〈1,3,4,2〉,〈4,2,1,3〉,〈3,1,2,4〉,〈2,4,3,1〉〉
139: 〈〈1,3,4,2〉,〈4,2,1,3〉,〈2,4,3,1〉,〈3,1,2,4〉〉, 140: 〈〈1,3,4,2〉,〈4,2,1,3〉,〈3,4,2,1〉,〈2,1,3,4〉〉
141: 〈〈1,3,4,2〉,〈4,2,3,1〉,〈3,1,2,4〉,〈2,4,1,3〉〉, 142: 〈〈1,3,4,2〉,〈4,2,3,1〉,〈2,4,1,3〉,〈3,1,2,4〉〉
143: 〈〈1,3,4,2〉,〈3,4,2,1〉,〈2,1,3,4〉,〈4,2,1,3〉〉, 144: 〈〈1,3,4,2〉,〈3,4,2,1〉,〈4,2,1,3〉,〈2,1,3,4〉〉
145: 〈〈3,1,2,4〉,〈1,2,4,3〉,〈2,4,3,1〉,〈4,3,1,2〉〉, 146: 〈〈3,1,2,4〉,〈1,2,4,3〉,〈4,3,1,2〉,〈2,4,3,1〉〉
147: 〈〈3,1,2,4〉,〈1,3,4,2〉,〈2,4,1,3〉,〈4,2,3,1〉〉, 148: 〈〈3,1,2,4〉,〈1,3,4,2〉,〈2,4,3,1〉,〈4,2,1,3〉〉
149: 〈〈3,1,2,4〉,〈1,3,4,2〉,〈4,2,1,3〉,〈2,4,3,1〉〉, 150: 〈〈3,1,2,4〉,〈1,3,4,2〉,〈4,2,3,1〉,〈2,4,1,3〉〉
151: 〈〈3,1,2,4〉,〈1,4,3,2〉,〈2,3,4,1〉,〈4,2,1,3〉〉, 152: 〈〈3,1,2,4〉,〈1,4,3,2〉,〈4,2,1,3〉,〈2,3,4,1〉〉
153: 〈〈3,1,2,4〉,〈2,3,4,1〉,〈1,4,3,2〉,〈4,2,1,3〉〉, 154: 〈〈3,1,2,4〉,〈2,3,4,1〉,〈4,2,1,3〉,〈1,4,3,2〉〉
155: 〈〈3,1,2,4〉,〈2,4,1,3〉,〈1,3,4,2〉,〈4,2,3,1〉〉, 156: 〈〈3,1,2,4〉,〈2,4,1,3〉,〈4,2,3,1〉,〈1,3,4,2〉〉
157: 〈〈3,1,2,4〉,〈2,4,3,1〉,〈1,2,4,3〉,〈4,3,1,2〉〉, 158: 〈〈3,1,2,4〉,〈2,4,3,1〉,〈1,3,4,2〉,〈4,2,1,3〉〉
159: 〈〈3,1,2,4〉,〈2,4,3,1〉,〈4,2,1,3〉,〈1,3,4,2〉〉, 160: 〈〈3,1,2,4〉,〈2,4,3,1〉,〈4,3,1,2〉,〈1,2,4,3〉〉
161: 〈〈3,1,2,4〉,〈4,2,1,3〉,〈1,3,4,2〉,〈2,4,3,1〉〉, 162: 〈〈3,1,2,4〉,〈4,2,1,3〉,〈1,4,3,2〉,〈2,3,4,1〉〉
163: 〈〈3,1,2,4〉,〈4,2,1,3〉,〈2,3,4,1〉,〈1,4,3,2〉〉, 164: 〈〈3,1,2,4〉,〈4,2,1,3〉,〈2,4,3,1〉,〈1,3,4,2〉〉
165: 〈〈3,1,2,4〉,〈4,2,3,1〉,〈1,3,4,2〉,〈2,4,1,3〉〉, 166: 〈〈3,1,2,4〉,〈4,2,3,1〉,〈2,4,1,3〉,〈1,3,4,2〉〉
167: 〈〈3,1,2,4〉,〈4,3,1,2〉,〈1,2,4,3〉,〈2,4,3,1〉〉, 168: 〈〈3,1,2,4〉,〈4,3,1,2〉,〈2,4,3,1〉,〈1,2,4,3〉〉
169: 〈〈3,1,4,2〉,〈1,2,3,4〉,〈2,4,1,3〉,〈4,3,2,1〉〉, 170: 〈〈3,1,4,2〉,〈1,2,3,4〉,〈4,3,2,1〉,〈2,4,1,3〉〉
171: 〈〈3,1,4,2〉,〈1,3,2,4〉,〈2,4,1,3〉,〈4,2,3,1〉〉, 172: 〈〈3,1,4,2〉,〈1,3,2,4〉,〈2,4,3,1〉,〈4,2,1,3〉〉
173: 〈〈3,1,4,2〉,〈1,3,2,4〉,〈4,2,1,3〉,〈2,4,3,1〉〉, 174: 〈〈3,1,4,2〉,〈1,3,2,4〉,〈4,2,3,1〉,〈2,4,1,3〉〉
175: 〈〈3,1,4,2〉,〈1,4,2,3〉,〈2,3,1,4〉,〈4,2,3,1〉〉, 176: 〈〈3,1,4,2〉,〈1,4,2,3〉,〈4,2,3,1〉,〈2,3,1,4〉〉
177: 〈〈3,1,4,2〉,〈2,3,1,4〉,〈1,4,2,3〉,〈4,2,3,1〉〉, 178: 〈〈3,1,4,2〉,〈2,3,1,4〉,〈4,2,3,1〉,〈1,4,2,3〉〉
179: 〈〈3,1,4,2〉,〈2,4,1,3〉,〈1,2,3,4〉,〈4,3,2,1〉〉, 180: 〈〈3,1,4,2〉,〈2,4,1,3〉,〈1,3,2,4〉,〈4,2,3,1〉〉
181: 〈〈3,1,4,2〉,〈2,4,1,3〉,〈4,2,3,1〉,〈1,3,2,4〉〉, 182: 〈〈3,1,4,2〉,〈2,4,1,3〉,〈4,3,2,1〉,〈1,2,3,4〉〉
183: 〈〈3,1,4,2〉,〈2,4,3,1〉,〈1,3,2,4〉,〈4,2,1,3〉〉, 184: 〈〈3,1,4,2〉,〈2,4,3,1〉,〈4,2,1,3〉,〈1,3,2,4〉〉
185: 〈〈3,1,4,2〉,〈4,2,1,3〉,〈1,3,2,4〉,〈2,4,3,1〉〉, 186: 〈〈3,1,4,2〉,〈4,2,1,3〉,〈2,4,3,1〉,〈1,3,2,4〉〉
187: 〈〈3,1,4,2〉,〈4,2,3,1〉,〈1,3,2,4〉,〈2,4,1,3〉〉, 188: 〈〈3,1,4,2〉,〈4,2,3,1〉,〈1,4,2,3〉,〈2,3,1,4〉〉
189: 〈〈3,1,4,2〉,〈4,2,3,1〉,〈2,3,1,4〉,〈1,4,2,3〉〉, 190: 〈〈3,1,4,2〉,〈4,2,3,1〉,〈2,4,1,3〉,〈1,3,2,4〉〉
191: 〈〈3,1,4,2〉,〈4,3,2,1〉,〈1,2,3,4〉,〈2,4,1,3〉〉, 192: 〈〈3,1,4,2〉,〈4,3,2,1〉,〈2,4,1,3〉,〈1,2,3,4〉〉
193: 〈〈1,4,2,3〉,〈2,1,3,4〉,〈3,2,4,1〉,〈4,3,1,2〉〉, 194: 〈〈1,4,2,3〉,〈2,1,3,4〉,〈4,3,1,2〉,〈3,2,4,1〉〉
195: 〈〈1,4,2,3〉,〈3,1,4,2〉,〈2,3,1,4〉,〈4,2,3,1〉〉, 196: 〈〈1,4,2,3〉,〈3,1,4,2〉,〈4,2,3,1〉,〈2,3,1,4〉〉
197: 〈〈1,4,2,3〉,〈4,1,3,2〉,〈2,3,1,4〉,〈3,2,4,1〉〉, 198: 〈〈1,4,2,3〉,〈4,1,3,2〉,〈2,3,4,1〉,〈3,2,1,4〉〉
199: 〈〈1,4,2,3〉,〈4,1,3,2〉,〈3,2,1,4〉,〈2,3,4,1〉〉, 200: 〈〈1,4,2,3〉,〈4,1,3,2〉,〈3,2,4,1〉,〈2,3,1,4〉〉
201: 〈〈1,4,2,3〉,〈2,3,1,4〉,〈3,1,4,2〉,〈4,2,3,1〉〉, 202: 〈〈1,4,2,3〉,〈2,3,1,4〉,〈4,1,3,2〉,〈3,2,4,1〉〉
203: 〈〈1,4,2,3〉,〈2,3,1,4〉,〈3,2,4,1〉,〈4,1,3,2〉〉, 204: 〈〈1,4,2,3〉,〈2,3,1,4〉,〈4,2,3,1〉,〈3,1,4,2〉〉
205: 〈〈1,4,2,3〉,〈2,3,4,1〉,〈4,1,3,2〉,〈3,2,1,4〉〉, 206: 〈〈1,4,2,3〉,〈2,3,4,1〉,〈3,2,1,4〉,〈4,1,3,2〉〉
207: 〈〈1,4,2,3〉,〈3,2,1,4〉,〈4,1,3,2〉,〈2,3,4,1〉〉, 208: 〈〈1,4,2,3〉,〈3,2,1,4〉,〈2,3,4,1〉,〈4,1,3,2〉〉
209: 〈〈1,4,2,3〉,〈3,2,4,1〉,〈2,1,3,4〉,〈4,3,1,2〉〉, 210: 〈〈1,4,2,3〉,〈3,2,4,1〉,〈4,1,3,2〉,〈2,3,1,4〉〉
211: 〈〈1,4,2,3〉,〈3,2,4,1〉,〈2,3,1,4〉,〈4,1,3,2〉〉, 212: 〈〈1,4,2,3〉,〈3,2,4,1〉,〈4,3,1,2〉,〈2,1,3,4〉〉
213: 〈〈1,4,2,3〉,〈4,2,3,1〉,〈3,1,4,2〉,〈2,3,1,4〉〉, 214: 〈〈1,4,2,3〉,〈4,2,3,1〉,〈2,3,1,4〉,〈3,1,4,2〉〉
215: 〈〈1,4,2,3〉,〈4,3,1,2〉,〈2,1,3,4〉,〈3,2,4,1〉〉, 216: 〈〈1,4,2,3〉,〈4,3,1,2〉,〈3,2,4,1〉,〈2,1,3,4〉〉
217: 〈〈1,4,3,2〉,〈2,1,4,3〉,〈3,2,1,4〉,〈4,3,2,1〉〉, 218: 〈〈1,4,3,2〉,〈2,1,4,3〉,〈4,3,2,1〉,〈3,2,1,4〉〉
219: 〈〈1,4,3,2〉,〈3,1,2,4〉,〈2,3,4,1〉,〈4,2,1,3〉〉, 220: 〈〈1,4,3,2〉,〈3,1,2,4〉,〈4,2,1,3〉,〈2,3,4,1〉〉
221: 〈〈1,4,3,2〉,〈4,1,2,3〉,〈2,3,1,4〉,〈3,2,4,1〉〉, 222: 〈〈1,4,3,2〉,〈4,1,2,3〉,〈2,3,4,1〉,〈3,2,1,4〉〉
223: 〈〈1,4,3,2〉,〈4,1,2,3〉,〈3,2,1,4〉,〈2,3,4,1〉〉, 224: 〈〈1,4,3,2〉,〈4,1,2,3〉,〈3,2,4,1〉,〈2,3,1,4〉〉
225: 〈〈1,4,3,2〉,〈2,3,1,4〉,〈4,1,2,3〉,〈3,2,4,1〉〉, 226: 〈〈1,4,3,2〉,〈2,3,1,4〉,〈3,2,4,1〉,〈4,1,2,3〉〉
227: 〈〈1,4,3,2〉,〈2,3,4,1〉,〈3,1,2,4〉,〈4,2,1,3〉〉, 228: 〈〈1,4,3,2〉,〈2,3,4,1〉,〈4,1,2,3〉,〈3,2,1,4〉〉
229: 〈〈1,4,3,2〉,〈2,3,4,1〉,〈3,2,1,4〉,〈4,1,2,3〉〉, 230: 〈〈1,4,3,2〉,〈2,3,4,1〉,〈4,2,1,3〉,〈3,1,2,4〉〉
231: 〈〈1,4,3,2〉,〈3,2,1,4〉,〈2,1,4,3〉,〈4,3,2,1〉〉, 232: 〈〈1,4,3,2〉,〈3,2,1,4〉,〈4,1,2,3〉,〈2,3,4,1〉〉
233: 〈〈1,4,3,2〉,〈3,2,1,4〉,〈2,3,4,1〉,〈4,1,2,3〉〉, 234: 〈〈1,4,3,2〉,〈3,2,1,4〉,〈4,3,2,1〉,〈2,1,4,3〉〉
235: 〈〈1,4,3,2〉,〈3,2,4,1〉,〈4,1,2,3〉,〈2,3,1,4〉〉, 236: 〈〈1,4,3,2〉,〈3,2,4,1〉,〈2,3,1,4〉,〈4,1,2,3〉〉
237: 〈〈1,4,3,2〉,〈4,2,1,3〉,〈3,1,2,4〉,〈2,3,4,1〉〉, 238: 〈〈1,4,3,2〉,〈4,2,1,3〉,〈2,3,4,1〉,〈3,1,2,4〉〉
239: 〈〈1,4,3,2〉,〈4,3,2,1〉,〈2,1,4,3〉,〈3,2,1,4〉〉, 240: 〈〈1,4,3,2〉,〈4,3,2,1〉,〈3,2,1,4〉,〈2,1,4,3〉〉
241: 〈〈4,1,2,3〉,〈1,2,3,4〉,〈2,3,4,1〉,〈3,4,1,2〉〉, 242: 〈〈4,1,2,3〉,〈1,2,3,4〉,〈3,4,1,2〉,〈2,3,4,1〉〉
243: 〈〈4,1,2,3〉,〈1,3,4,2〉,〈3,2,1,4〉,〈2,4,3,1〉〉, 244: 〈〈4,1,2,3〉,〈1,3,4,2〉,〈2,4,3,1〉,〈3,2,1,4〉〉
245: 〈〈4,1,2,3〉,〈1,4,3,2〉,〈2,3,1,4〉,〈3,2,4,1〉〉, 246: 〈〈4,1,2,3〉,〈1,4,3,2〉,〈2,3,4,1〉,〈3,2,1,4〉〉
247: 〈〈4,1,2,3〉,〈1,4,3,2〉,〈3,2,1,4〉,〈2,3,4,1〉〉, 248: 〈〈4,1,2,3〉,〈1,4,3,2〉,〈3,2,4,1〉,〈2,3,1,4〉〉
249: 〈〈4,1,2,3〉,〈2,3,1,4〉,〈1,4,3,2〉,〈3,2,4,1〉〉, 250: 〈〈4,1,2,3〉,〈2,3,1,4〉,〈3,2,4,1〉,〈1,4,3,2〉〉
251: 〈〈4,1,2,3〉,〈2,3,4,1〉,〈1,2,3,4〉,〈3,4,1,2〉〉, 252: 〈〈4,1,2,3〉,〈2,3,4,1〉,〈1,4,3,2〉,〈3,2,1,4〉〉
253: 〈〈4,1,2,3〉,〈2,3,4,1〉,〈3,2,1,4〉,〈1,4,3,2〉〉, 254: 〈〈4,1,2,3〉,〈2,3,4,1〉,〈3,4,1,2〉,〈1,2,3,4〉〉
255: 〈〈4,1,2,3〉,〈3,2,1,4〉,〈1,3,4,2〉,〈2,4,3,1〉〉, 256: 〈〈4,1,2,3〉,〈3,2,1,4〉,〈1,4,3,2〉,〈2,3,4,1〉〉
257: 〈〈4,1,2,3〉,〈3,2,1,4〉,〈2,3,4,1〉,〈1,4,3,2〉〉, 258: 〈〈4,1,2,3〉,〈3,2,1,4〉,〈2,4,3,1〉,〈1,3,4,2〉〉
259: 〈〈4,1,2,3〉,〈3,2,4,1〉,〈1,4,3,2〉,〈2,3,1,4〉〉, 260: 〈〈4,1,2,3〉,〈3,2,4,1〉,〈2,3,1,4〉,〈1,4,3,2〉〉
261: 〈〈4,1,2,3〉,〈2,4,3,1〉,〈1,3,4,2〉,〈3,2,1,4〉〉, 262: 〈〈4,1,2,3〉,〈2,4,3,1〉,〈3,2,1,4〉,〈1,3,4,2〉〉
263: 〈〈4,1,2,3〉,〈3,4,1,2〉,〈1,2,3,4〉,〈2,3,4,1〉〉, 264: 〈〈4,1,2,3〉,〈3,4,1,2〉,〈2,3,4,1〉,〈1,2,3,4〉〉
265: 〈〈4,1,3,2〉,〈1,2,4,3〉,〈2,3,1,4〉,〈3,4,2,1〉〉, 266: 〈〈4,1,3,2〉,〈1,2,4,3〉,〈3,4,2,1〉,〈2,3,1,4〉〉
267: 〈〈4,1,3,2〉,〈1,3,2,4〉,〈3,2,4,1〉,〈2,4,1,3〉〉, 268: 〈〈4,1,3,2〉,〈1,3,2,4〉,〈2,4,1,3〉,〈3,2,4,1〉〉
269: 〈〈4,1,3,2〉,〈1,4,2,3〉,〈2,3,1,4〉,〈3,2,4,1〉〉, 270: 〈〈4,1,3,2〉,〈1,4,2,3〉,〈2,3,4,1〉,〈3,2,1,4〉〉
271: 〈〈4,1,3,2〉,〈1,4,2,3〉,〈3,2,1,4〉,〈2,3,4,1〉〉, 272: 〈〈4,1,3,2〉,〈1,4,2,3〉,〈3,2,4,1〉,〈2,3,1,4〉〉
273: 〈〈4,1,3,2〉,〈2,3,1,4〉,〈1,2,4,3〉,〈3,4,2,1〉〉, 274: 〈〈4,1,3,2〉,〈2,3,1,4〉,〈1,4,2,3〉,〈3,2,4,1〉〉
275: 〈〈4,1,3,2〉,〈2,3,1,4〉,〈3,2,4,1〉,〈1,4,2,3〉〉, 276: 〈〈4,1,3,2〉,〈2,3,1,4〉,〈3,4,2,1〉,〈1,2,4,3〉〉
277: 〈〈4,1,3,2〉,〈2,3,4,1〉,〈1,4,2,3〉,〈3,2,1,4〉〉, 278: 〈〈4,1,3,2〉,〈2,3,4,1〉,〈3,2,1,4〉,〈1,4,2,3〉〉
279: 〈〈4,1,3,2〉,〈3,2,1,4〉,〈1,4,2,3〉,〈2,3,4,1〉〉, 280: 〈〈4,1,3,2〉,〈3,2,1,4〉,〈2,3,4,1〉,〈1,4,2,3〉〉
281: 〈〈4,1,3,2〉,〈3,2,4,1〉,〈1,3,2,4〉,〈2,4,1,3〉〉, 282: 〈〈4,1,3,2〉,〈3,2,4,1〉,〈1,4,2,3〉,〈2,3,1,4〉〉
283: 〈〈4,1,3,2〉,〈3,2,4,1〉,〈2,3,1,4〉,〈1,4,2,3〉〉, 284: 〈〈4,1,3,2〉,〈3,2,4,1〉,〈2,4,1,3〉,〈1,3,2,4〉〉
285: 〈〈4,1,3,2〉,〈2,4,1,3〉,〈1,3,2,4〉,〈3,2,4,1〉〉, 286: 〈〈4,1,3,2〉,〈2,4,1,3〉,〈3,2,4,1〉,〈1,3,2,4〉〉
287: 〈〈4,1,3,2〉,〈3,4,2,1〉,〈1,2,4,3〉,〈2,3,1,4〉〉, 288: 〈〈4,1,3,2〉,〈3,4,2,1〉,〈2,3,1,4〉,〈1,2,4,3〉〉
289: 〈〈2,3,1,4〉,〈1,2,4,3〉,〈4,1,3,2〉,〈3,4,2,1〉〉, 290: 〈〈2,3,1,4〉,〈1,2,4,3〉,〈3,4,2,1〉,〈4,1,3,2〉〉
291: 〈〈2,3,1,4〉,〈3,1,4,2〉,〈1,4,2,3〉,〈4,2,3,1〉〉, 292: 〈〈2,3,1,4〉,〈3,1,4,2〉,〈4,2,3,1〉,〈1,4,2,3〉〉
293: 〈〈2,3,1,4〉,〈1,4,2,3〉,〈3,1,4,2〉,〈4,2,3,1〉〉, 294: 〈〈2,3,1,4〉,〈1,4,2,3〉,〈4,1,3,2〉,〈3,2,4,1〉〉
295: 〈〈2,3,1,4〉,〈1,4,2,3〉,〈3,2,4,1〉,〈4,1,3,2〉〉, 296: 〈〈2,3,1,4〉,〈1,4,2,3〉,〈4,2,3,1〉,〈3,1,4,2〉〉
297: 〈〈2,3,1,4〉,〈1,4,3,2〉,〈4,1,2,3〉,〈3,2,4,1〉〉, 298: 〈〈2,3,1,4〉,〈1,4,3,2〉,〈3,2,4,1〉,〈4,1,2,3〉〉
299: 〈〈2,3,1,4〉,〈4,1,2,3〉,〈1,4,3,2〉,〈3,2,4,1〉〉, 300: 〈〈2,3,1,4〉,〈4,1,2,3〉,〈3,2,4,1〉,〈1,4,3,2〉〉
301: 〈〈2,3,1,4〉,〈4,1,3,2〉,〈1,2,4,3〉,〈3,4,2,1〉〉, 302: 〈〈2,3,1,4〉,〈4,1,3,2〉,〈1,4,2,3〉,〈3,2,4,1〉〉
303: 〈〈2,3,1,4〉,〈4,1,3,2〉,〈3,2,4,1〉,〈1,4,2,3〉〉, 304: 〈〈2,3,1,4〉,〈4,1,3,2〉,〈3,4,2,1〉,〈1,2,4,3〉〉
305: 〈〈2,3,1,4〉,〈3,2,4,1〉,〈1,4,2,3〉,〈4,1,3,2〉〉, 306: 〈〈2,3,1,4〉,〈3,2,4,1〉,〈1,4,3,2〉,〈4,1,2,3〉〉
307: 〈〈2,3,1,4〉,〈3,2,4,1〉,〈4,1,2,3〉,〈1,4,3,2〉〉, 308: 〈〈2,3,1,4〉,〈3,2,4,1〉,〈4,1,3,2〉,〈1,4,2,3〉〉
309: 〈〈2,3,1,4〉,〈4,2,3,1〉,〈3,1,4,2〉,〈1,4,2,3〉〉, 310: 〈〈2,3,1,4〉,〈4,2,3,1〉,〈1,4,2,3〉,〈3,1,4,2〉〉
311: 〈〈2,3,1,4〉,〈3,4,2,1〉,〈1,2,4,3〉,〈4,1,3,2〉〉, 312: 〈〈2,3,1,4〉,〈3,4,2,1〉,〈4,1,3,2〉,〈1,2,4,3〉〉
313: 〈〈2,3,4,1〉,〈1,2,3,4〉,〈4,1,2,3〉,〈3,4,1,2〉〉, 314: 〈〈2,3,4,1〉,〈1,2,3,4〉,〈3,4,1,2〉,〈4,1,2,3〉〉
315: 〈〈2,3,4,1〉,〈3,1,2,4〉,〈1,4,3,2〉,〈4,2,1,3〉〉, 316: 〈〈2,3,4,1〉,〈3,1,2,4〉,〈4,2,1,3〉,〈1,4,3,2〉〉
317: 〈〈2,3,4,1〉,〈1,4,2,3〉,〈4,1,3,2〉,〈3,2,1,4〉〉, 318: 〈〈2,3,4,1〉,〈1,4,2,3〉,〈3,2,1,4〉,〈4,1,3,2〉〉
319: 〈〈2,3,4,1〉,〈1,4,3,2〉,〈3,1,2,4〉,〈4,2,1,3〉〉, 320: 〈〈2,3,4,1〉,〈1,4,3,2〉,〈4,1,2,3〉,〈3,2,1,4〉〉
321: 〈〈2,3,4,1〉,〈1,4,3,2〉,〈3,2,1,4〉,〈4,1,2,3〉〉, 322: 〈〈2,3,4,1〉,〈1,4,3,2〉,〈4,2,1,3〉,〈3,1,2,4〉〉
323: 〈〈2,3,4,1〉,〈4,1,2,3〉,〈1,2,3,4〉,〈3,4,1,2〉〉, 324: 〈〈2,3,4,1〉,〈4,1,2,3〉,〈1,4,3,2〉,〈3,2,1,4〉〉
325: 〈〈2,3,4,1〉,〈4,1,2,3〉,〈3,2,1,4〉,〈1,4,3,2〉〉, 326: 〈〈2,3,4,1〉,〈4,1,2,3〉,〈3,4,1,2〉,〈1,2,3,4〉〉
327: 〈〈2,3,4,1〉,〈4,1,3,2〉,〈1,4,2,3〉,〈3,2,1,4〉〉, 328: 〈〈2,3,4,1〉,〈4,1,3,2〉,〈3,2,1,4〉,〈1,4,2,3〉〉
329: 〈〈2,3,4,1〉,〈3,2,1,4〉,〈1,4,2,3〉,〈4,1,3,2〉〉, 330: 〈〈2,3,4,1〉,〈3,2,1,4〉,〈1,4,3,2〉,〈4,1,2,3〉〉
331: 〈〈2,3,4,1〉,〈3,2,1,4〉,〈4,1,2,3〉,〈1,4,3,2〉〉, 332: 〈〈2,3,4,1〉,〈3,2,1,4〉,〈4,1,3,2〉,〈1,4,2,3〉〉
333: 〈〈2,3,4,1〉,〈4,2,1,3〉,〈3,1,2,4〉,〈1,4,3,2〉〉, 334: 〈〈2,3,4,1〉,〈4,2,1,3〉,〈1,4,3,2〉,〈3,1,2,4〉〉
335: 〈〈2,3,4,1〉,〈3,4,1,2〉,〈1,2,3,4〉,〈4,1,2,3〉〉, 336: 〈〈2,3,4,1〉,〈3,4,1,2〉,〈4,1,2,3〉,〈1,2,3,4〉〉
337: 〈〈3,2,1,4〉,〈2,1,4,3〉,〈1,4,3,2〉,〈4,3,2,1〉〉, 338: 〈〈3,2,1,4〉,〈2,1,4,3〉,〈4,3,2,1〉,〈1,4,3,2〉〉
339: 〈〈3,2,1,4〉,〈1,3,4,2〉,〈4,1,2,3〉,〈2,4,3,1〉〉, 340: 〈〈3,2,1,4〉,〈1,3,4,2〉,〈2,4,3,1〉,〈4,1,2,3〉〉
341: 〈〈3,2,1,4〉,〈1,4,2,3〉,〈4,1,3,2〉,〈2,3,4,1〉〉, 342: 〈〈3,2,1,4〉,〈1,4,2,3〉,〈2,3,4,1〉,〈4,1,3,2〉〉
343: 〈〈3,2,1,4〉,〈1,4,3,2〉,〈2,1,4,3〉,〈4,3,2,1〉〉, 344: 〈〈3,2,1,4〉,〈1,4,3,2〉,〈4,1,2,3〉,〈2,3,4,1〉〉
345: 〈〈3,2,1,4〉,〈1,4,3,2〉,〈2,3,4,1〉,〈4,1,2,3〉〉, 346: 〈〈3,2,1,4〉,〈1,4,3,2〉,〈4,3,2,1〉,〈2,1,4,3〉〉
347: 〈〈3,2,1,4〉,〈4,1,2,3〉,〈1,3,4,2〉,〈2,4,3,1〉〉, 348: 〈〈3,2,1,4〉,〈4,1,2,3〉,〈1,4,3,2〉,〈2,3,4,1〉〉
349: 〈〈3,2,1,4〉,〈4,1,2,3〉,〈2,3,4,1〉,〈1,4,3,2〉〉, 350: 〈〈3,2,1,4〉,〈4,1,2,3〉,〈2,4,3,1〉,〈1,3,4,2〉〉
351: 〈〈3,2,1,4〉,〈4,1,3,2〉,〈1,4,2,3〉,〈2,3,4,1〉〉, 352: 〈〈3,2,1,4〉,〈4,1,3,2〉,〈2,3,4,1〉,〈1,4,2,3〉〉
353: 〈〈3,2,1,4〉,〈2,3,4,1〉,〈1,4,2,3〉,〈4,1,3,2〉〉, 354: 〈〈3,2,1,4〉,〈2,3,4,1〉,〈1,4,3,2〉,〈4,1,2,3〉〉
355: 〈〈3,2,1,4〉,〈2,3,4,1〉,〈4,1,2,3〉,〈1,4,3,2〉〉, 356: 〈〈3,2,1,4〉,〈2,3,4,1〉,〈4,1,3,2〉,〈1,4,2,3〉〉
357: 〈〈3,2,1,4〉,〈2,4,3,1〉,〈1,3,4,2〉,〈4,1,2,3〉〉, 358: 〈〈3,2,1,4〉,〈2,4,3,1〉,〈4,1,2,3〉,〈1,3,4,2〉〉
359: 〈〈3,2,1,4〉,〈4,3,2,1〉,〈2,1,4,3〉,〈1,4,3,2〉〉, 360: 〈〈3,2,1,4〉,〈4,3,2,1〉,〈1,4,3,2〉,〈2,1,4,3〉〉
361: 〈〈3,2,4,1〉,〈2,1,3,4〉,〈1,4,2,3〉,〈4,3,1,2〉〉, 362: 〈〈3,2,4,1〉,〈2,1,3,4〉,〈4,3,1,2〉,〈1,4,2,3〉〉
363: 〈〈3,2,4,1〉,〈1,3,2,4〉,〈4,1,3,2〉,〈2,4,1,3〉〉, 364: 〈〈3,2,4,1〉,〈1,3,2,4〉,〈2,4,1,3〉,〈4,1,3,2〉〉
365: 〈〈3,2,4,1〉,〈1,4,2,3〉,〈2,1,3,4〉,〈4,3,1,2〉〉, 366: 〈〈3,2,4,1〉,〈1,4,2,3〉,〈4,1,3,2〉,〈2,3,1,4〉〉
367: 〈〈3,2,4,1〉,〈1,4,2,3〉,〈2,3,1,4〉,〈4,1,3,2〉〉, 368: 〈〈3,2,4,1〉,〈1,4,2,3〉,〈4,3,1,2〉,〈2,1,3,4〉〉
369: 〈〈3,2,4,1〉,〈1,4,3,2〉,〈4,1,2,3〉,〈2,3,1,4〉〉, 370: 〈〈3,2,4,1〉,〈1,4,3,2〉,〈2,3,1,4〉,〈4,1,2,3〉〉
371: 〈〈3,2,4,1〉,〈4,1,2,3〉,〈1,4,3,2〉,〈2,3,1,4〉〉, 372: 〈〈3,2,4,1〉,〈4,1,2,3〉,〈2,3,1,4〉,〈1,4,3,2〉〉
373: 〈〈3,2,4,1〉,〈4,1,3,2〉,〈1,3,2,4〉,〈2,4,1,3〉〉, 374: 〈〈3,2,4,1〉,〈4,1,3,2〉,〈1,4,2,3〉,〈2,3,1,4〉〉
375: 〈〈3,2,4,1〉,〈4,1,3,2〉,〈2,3,1,4〉,〈1,4,2,3〉〉, 376: 〈〈3,2,4,1〉,〈4,1,3,2〉,〈2,4,1,3〉,〈1,3,2,4〉〉
377: 〈〈3,2,4,1〉,〈2,3,1,4〉,〈1,4,2,3〉,〈4,1,3,2〉〉, 378: 〈〈3,2,4,1〉,〈2,3,1,4〉,〈1,4,3,2〉,〈4,1,2,3〉〉
379: 〈〈3,2,4,1〉,〈2,3,1,4〉,〈4,1,2,3〉,〈1,4,3,2〉〉, 380: 〈〈3,2,4,1〉,〈2,3,1,4〉,〈4,1,3,2〉,〈1,4,2,3〉〉
381: 〈〈3,2,4,1〉,〈2,4,1,3〉,〈1,3,2,4〉,〈4,1,3,2〉〉, 382: 〈〈3,2,4,1〉,〈2,4,1,3〉,〈4,1,3,2〉,〈1,3,2,4〉〉
383: 〈〈3,2,4,1〉,〈4,3,1,2〉,〈2,1,3,4〉,〈1,4,2,3〉〉, 384: 〈〈3,2,4,1〉,〈4,3,1,2〉,〈1,4,2,3〉,〈2,1,3,4〉〉
385: 〈〈2,4,1,3〉,〈1,2,3,4〉,〈3,1,4,2〉,〈4,3,2,1〉〉, 386: 〈〈2,4,1,3〉,〈1,2,3,4〉,〈4,3,2,1〉,〈3,1,4,2〉〉
387: 〈〈2,4,1,3〉,〈1,3,2,4〉,〈3,1,4,2〉,〈4,2,3,1〉〉, 388: 〈〈2,4,1,3〉,〈1,3,2,4〉,〈4,1,3,2〉,〈3,2,4,1〉〉
389: 〈〈2,4,1,3〉,〈1,3,2,4〉,〈3,2,4,1〉,〈4,1,3,2〉〉, 390: 〈〈2,4,1,3〉,〈1,3,2,4〉,〈4,2,3,1〉,〈3,1,4,2〉〉
391: 〈〈2,4,1,3〉,〈1,3,4,2〉,〈3,1,2,4〉,〈4,2,3,1〉〉, 392: 〈〈2,4,1,3〉,〈1,3,4,2〉,〈4,2,3,1〉,〈3,1,2,4〉〉
393: 〈〈2,4,1,3〉,〈3,1,2,4〉,〈1,3,4,2〉,〈4,2,3,1〉〉, 394: 〈〈2,4,1,3〉,〈3,1,2,4〉,〈4,2,3,1〉,〈1,3,4,2〉〉
395: 〈〈2,4,1,3〉,〈3,1,4,2〉,〈1,2,3,4〉,〈4,3,2,1〉〉, 396: 〈〈2,4,1,3〉,〈3,1,4,2〉,〈1,3,2,4〉,〈4,2,3,1〉〉
397: 〈〈2,4,1,3〉,〈3,1,4,2〉,〈4,2,3,1〉,〈1,3,2,4〉〉, 398: 〈〈2,4,1,3〉,〈3,1,4,2〉,〈4,3,2,1〉,〈1,2,3,4〉〉
399: 〈〈2,4,1,3〉,〈4,1,3,2〉,〈1,3,2,4〉,〈3,2,4,1〉〉, 400: 〈〈2,4,1,3〉,〈4,1,3,2〉,〈3,2,4,1〉,〈1,3,2,4〉〉
401: 〈〈2,4,1,3〉,〈3,2,4,1〉,〈1,3,2,4〉,〈4,1,3,2〉〉, 402: 〈〈2,4,1,3〉,〈3,2,4,1〉,〈4,1,3,2〉,〈1,3,2,4〉〉
403: 〈〈2,4,1,3〉,〈4,2,3,1〉,〈1,3,2,4〉,〈3,1,4,2〉〉, 404: 〈〈2,4,1,3〉,〈4,2,3,1〉,〈1,3,4,2〉,〈3,1,2,4〉〉
405: 〈〈2,4,1,3〉,〈4,2,3,1〉,〈3,1,2,4〉,〈1,3,4,2〉〉, 406: 〈〈2,4,1,3〉,〈4,2,3,1〉,〈3,1,4,2〉,〈1,3,2,4〉〉
407: 〈〈2,4,1,3〉,〈4,3,2,1〉,〈1,2,3,4〉,〈3,1,4,2〉〉, 408: 〈〈2,4,1,3〉,〈4,3,2,1〉,〈3,1,4,2〉,〈1,2,3,4〉〉
409: 〈〈2,4,3,1〉,〈1,2,4,3〉,〈3,1,2,4〉,〈4,3,1,2〉〉, 410: 〈〈2,4,3,1〉,〈1,2,4,3〉,〈4,3,1,2〉,〈3,1,2,4〉〉
411: 〈〈2,4,3,1〉,〈1,3,2,4〉,〈3,1,4,2〉,〈4,2,1,3〉〉, 412: 〈〈2,4,3,1〉,〈1,3,2,4〉,〈4,2,1,3〉,〈3,1,4,2〉〉
413: 〈〈2,4,3,1〉,〈1,3,4,2〉,〈3,1,2,4〉,〈4,2,1,3〉〉, 414: 〈〈2,4,3,1〉,〈1,3,4,2〉,〈4,1,2,3〉,〈3,2,1,4〉〉
415: 〈〈2,4,3,1〉,〈1,3,4,2〉,〈3,2,1,4〉,〈4,1,2,3〉〉, 416: 〈〈2,4,3,1〉,〈1,3,4,2〉,〈4,2,1,3〉,〈3,1,2,4〉〉
417: 〈〈2,4,3,1〉,〈3,1,2,4〉,〈1,2,4,3〉,〈4,3,1,2〉〉, 418: 〈〈2,4,3,1〉,〈3,1,2,4〉,〈1,3,4,2〉,〈4,2,1,3〉〉
419: 〈〈2,4,3,1〉,〈3,1,2,4〉,〈4,2,1,3〉,〈1,3,4,2〉〉, 420: 〈〈2,4,3,1〉,〈3,1,2,4〉,〈4,3,1,2〉,〈1,2,4,3〉〉
421: 〈〈2,4,3,1〉,〈3,1,4,2〉,〈1,3,2,4〉,〈4,2,1,3〉〉, 422: 〈〈2,4,3,1〉,〈3,1,4,2〉,〈4,2,1,3〉,〈1,3,2,4〉〉
423: 〈〈2,4,3,1〉,〈4,1,2,3〉,〈1,3,4,2〉,〈3,2,1,4〉〉, 424: 〈〈2,4,3,1〉,〈4,1,2,3〉,〈3,2,1,4〉,〈1,3,4,2〉〉
425: 〈〈2,4,3,1〉,〈3,2,1,4〉,〈1,3,4,2〉,〈4,1,2,3〉〉, 426: 〈〈2,4,3,1〉,〈3,2,1,4〉,〈4,1,2,3〉,〈1,3,4,2〉〉
427: 〈〈2,4,3,1〉,〈4,2,1,3〉,〈1,3,2,4〉,〈3,1,4,2〉〉, 428: 〈〈2,4,3,1〉,〈4,2,1,3〉,〈1,3,4,2〉,〈3,1,2,4〉〉
429: 〈〈2,4,3,1〉,〈4,2,1,3〉,〈3,1,2,4〉,〈1,3,4,2〉〉, 430: 〈〈2,4,3,1〉,〈4,2,1,3〉,〈3,1,4,2〉,〈1,3,2,4〉〉
431: 〈〈2,4,3,1〉,〈4,3,1,2〉,〈1,2,4,3〉,〈3,1,2,4〉〉, 432: 〈〈2,4,3,1〉,〈4,3,1,2〉,〈3,1,2,4〉,〈1,2,4,3〉〉
433: 〈〈4,2,1,3〉,〈2,1,3,4〉,〈1,3,4,2〉,〈3,4,2,1〉〉, 434: 〈〈4,2,1,3〉,〈2,1,3,4〉,〈3,4,2,1〉,〈1,3,4,2〉〉
435: 〈〈4,2,1,3〉,〈1,3,2,4〉,〈3,1,4,2〉,〈2,4,3,1〉〉, 436: 〈〈4,2,1,3〉,〈1,3,2,4〉,〈2,4,3,1〉,〈3,1,4,2〉〉
437: 〈〈4,2,1,3〉,〈1,3,4,2〉,〈2,1,3,4〉,〈3,4,2,1〉〉, 438: 〈〈4,2,1,3〉,〈1,3,4,2〉,〈3,1,2,4〉,〈2,4,3,1〉〉
439: 〈〈4,2,1,3〉,〈1,3,4,2〉,〈2,4,3,1〉,〈3,1,2,4〉〉, 440: 〈〈4,2,1,3〉,〈1,3,4,2〉,〈3,4,2,1〉,〈2,1,3,4〉〉
441: 〈〈4,2,1,3〉,〈3,1,2,4〉,〈1,3,4,2〉,〈2,4,3,1〉〉, 442: 〈〈4,2,1,3〉,〈3,1,2,4〉,〈1,4,3,2〉,〈2,3,4,1〉〉
443: 〈〈4,2,1,3〉,〈3,1,2,4〉,〈2,3,4,1〉,〈1,4,3,2〉〉, 444: 〈〈4,2,1,3〉,〈3,1,2,4〉,〈2,4,3,1〉,〈1,3,4,2〉〉
445: 〈〈4,2,1,3〉,〈3,1,4,2〉,〈1,3,2,4〉,〈2,4,3,1〉〉, 446: 〈〈4,2,1,3〉,〈3,1,4,2〉,〈2,4,3,1〉,〈1,3,2,4〉〉
447: 〈〈4,2,1,3〉,〈1,4,3,2〉,〈3,1,2,4〉,〈2,3,4,1〉〉, 448: 〈〈4,2,1,3〉,〈1,4,3,2〉,〈2,3,4,1〉,〈3,1,2,4〉〉
449: 〈〈4,2,1,3〉,〈2,3,4,1〉,〈3,1,2,4〉,〈1,4,3,2〉〉, 450: 〈〈4,2,1,3〉,〈2,3,4,1〉,〈1,4,3,2〉,〈3,1,2,4〉〉
451: 〈〈4,2,1,3〉,〈2,4,3,1〉,〈1,3,2,4〉,〈3,1,4,2〉〉, 452: 〈〈4,2,1,3〉,〈2,4,3,1〉,〈1,3,4,2〉,〈3,1,2,4〉〉
453: 〈〈4,2,1,3〉,〈2,4,3,1〉,〈3,1,2,4〉,〈1,3,4,2〉〉, 454: 〈〈4,2,1,3〉,〈2,4,3,1〉,〈3,1,4,2〉,〈1,3,2,4〉〉
455: 〈〈4,2,1,3〉,〈3,4,2,1〉,〈2,1,3,4〉,〈1,3,4,2〉〉, 456: 〈〈4,2,1,3〉,〈3,4,2,1〉,〈1,3,4,2〉,〈2,1,3,4〉〉
457: 〈〈4,2,3,1〉,〈2,1,4,3〉,〈1,3,2,4〉,〈3,4,1,2〉〉, 458: 〈〈4,2,3,1〉,〈2,1,4,3〉,〈3,4,1,2〉,〈1,3,2,4〉〉
459: 〈〈4,2,3,1〉,〈1,3,2,4〉,〈2,1,4,3〉,〈3,4,1,2〉〉, 460: 〈〈4,2,3,1〉,〈1,3,2,4〉,〈3,1,4,2〉,〈2,4,1,3〉〉
461: 〈〈4,2,3,1〉,〈1,3,2,4〉,〈2,4,1,3〉,〈3,1,4,2〉〉, 462: 〈〈4,2,3,1〉,〈1,3,2,4〉,〈3,4,1,2〉,〈2,1,4,3〉〉
463: 〈〈4,2,3,1〉,〈1,3,4,2〉,〈3,1,2,4〉,〈2,4,1,3〉〉, 464: 〈〈4,2,3,1〉,〈1,3,4,2〉,〈2,4,1,3〉,〈3,1,2,4〉〉
465: 〈〈4,2,3,1〉,〈3,1,2,4〉,〈1,3,4,2〉,〈2,4,1,3〉〉, 466: 〈〈4,2,3,1〉,〈3,1,2,4〉,〈2,4,1,3〉,〈1,3,4,2〉〉
467: 〈〈4,2,3,1〉,〈3,1,4,2〉,〈1,3,2,4〉,〈2,4,1,3〉〉, 468: 〈〈4,2,3,1〉,〈3,1,4,2〉,〈1,4,2,3〉,〈2,3,1,4〉〉
469: 〈〈4,2,3,1〉,〈3,1,4,2〉,〈2,3,1,4〉,〈1,4,2,3〉〉, 470: 〈〈4,2,3,1〉,〈3,1,4,2〉,〈2,4,1,3〉,〈1,3,2,4〉〉
471: 〈〈4,2,3,1〉,〈1,4,2,3〉,〈3,1,4,2〉,〈2,3,1,4〉〉, 472: 〈〈4,2,3,1〉,〈1,4,2,3〉,〈2,3,1,4〉,〈3,1,4,2〉〉
473: 〈〈4,2,3,1〉,〈2,3,1,4〉,〈3,1,4,2〉,〈1,4,2,3〉〉, 474: 〈〈4,2,3,1〉,〈2,3,1,4〉,〈1,4,2,3〉,〈3,1,4,2〉〉
475: 〈〈4,2,3,1〉,〈2,4,1,3〉,〈1,3,2,4〉,〈3,1,4,2〉〉, 476: 〈〈4,2,3,1〉,〈2,4,1,3〉,〈1,3,4,2〉,〈3,1,2,4〉〉
477: 〈〈4,2,3,1〉,〈2,4,1,3〉,〈3,1,2,4〉,〈1,3,4,2〉〉, 478: 〈〈4,2,3,1〉,〈2,4,1,3〉,〈3,1,4,2〉,〈1,3,2,4〉〉
479: 〈〈4,2,3,1〉,〈3,4,1,2〉,〈2,1,4,3〉,〈1,3,2,4〉〉, 480: 〈〈4,2,3,1〉,〈3,4,1,2〉,〈1,3,2,4〉,〈2,1,4,3〉〉
481: 〈〈3,4,1,2〉,〈1,2,3,4〉,〈2,1,4,3〉,〈4,3,2,1〉〉, 482: 〈〈3,4,1,2〉,〈1,2,3,4〉,〈4,1,2,3〉,〈2,3,4,1〉〉
483: 〈〈3,4,1,2〉,〈1,2,3,4〉,〈2,3,4,1〉,〈4,1,2,3〉〉, 484: 〈〈3,4,1,2〉,〈1,2,3,4〉,〈4,3,2,1〉,〈2,1,4,3〉〉
485: 〈〈3,4,1,2〉,〈1,2,4,3〉,〈2,1,3,4〉,〈4,3,2,1〉〉, 486: 〈〈3,4,1,2〉,〈1,2,4,3〉,〈4,3,2,1〉,〈2,1,3,4〉〉
487: 〈〈3,4,1,2〉,〈2,1,3,4〉,〈1,2,4,3〉,〈4,3,2,1〉〉, 488: 〈〈3,4,1,2〉,〈2,1,3,4〉,〈4,3,2,1〉,〈1,2,4,3〉〉
489: 〈〈3,4,1,2〉,〈2,1,4,3〉,〈1,2,3,4〉,〈4,3,2,1〉〉, 490: 〈〈3,4,1,2〉,〈2,1,4,3〉,〈1,3,2,4〉,〈4,2,3,1〉〉
491: 〈〈3,4,1,2〉,〈2,1,4,3〉,〈4,2,3,1〉,〈1,3,2,4〉〉, 492: 〈〈3,4,1,2〉,〈2,1,4,3〉,〈4,3,2,1〉,〈1,2,3,4〉〉
493: 〈〈3,4,1,2〉,〈1,3,2,4〉,〈2,1,4,3〉,〈4,2,3,1〉〉, 494: 〈〈3,4,1,2〉,〈1,3,2,4〉,〈4,2,3,1〉,〈2,1,4,3〉〉
495: 〈〈3,4,1,2〉,〈4,1,2,3〉,〈1,2,3,4〉,〈2,3,4,1〉〉, 496: 〈〈3,4,1,2〉,〈4,1,2,3〉,〈2,3,4,1〉,〈1,2,3,4〉〉
497: 〈〈3,4,1,2〉,〈2,3,4,1〉,〈1,2,3,4〉,〈4,1,2,3〉〉, 498: 〈〈3,4,1,2〉,〈2,3,4,1〉,〈4,1,2,3〉,〈1,2,3,4〉〉
499: 〈〈3,4,1,2〉,〈4,2,3,1〉,〈2,1,4,3〉,〈1,3,2,4〉〉, 500: 〈〈3,4,1,2〉,〈4,2,3,1〉,〈1,3,2,4〉,〈2,1,4,3〉〉
501: 〈〈3,4,1,2〉,〈4,3,2,1〉,〈1,2,3,4〉,〈2,1,4,3〉〉, 502: 〈〈3,4,1,2〉,〈4,3,2,1〉,〈1,2,4,3〉,〈2,1,3,4〉〉
503: 〈〈3,4,1,2〉,〈4,3,2,1〉,〈2,1,3,4〉,〈1,2,4,3〉〉, 504: 〈〈3,4,1,2〉,〈4,3,2,1〉,〈2,1,4,3〉,〈1,2,3,4〉〉
505: 〈〈3,4,2,1〉,〈1,2,3,4〉,〈2,1,4,3〉,〈4,3,1,2〉〉, 506: 〈〈3,4,2,1〉,〈1,2,3,4〉,〈4,3,1,2〉,〈2,1,4,3〉〉
507: 〈〈3,4,2,1〉,〈1,2,4,3〉,〈2,1,3,4〉,〈4,3,1,2〉〉, 508: 〈〈3,4,2,1〉,〈1,2,4,3〉,〈4,1,3,2〉,〈2,3,1,4〉〉
509: 〈〈3,4,2,1〉,〈1,2,4,3〉,〈2,3,1,4〉,〈4,1,3,2〉〉, 510: 〈〈3,4,2,1〉,〈1,2,4,3〉,〈4,3,1,2〉,〈2,1,3,4〉〉
511: 〈〈3,4,2,1〉,〈2,1,3,4〉,〈1,2,4,3〉,〈4,3,1,2〉〉, 512: 〈〈3,4,2,1〉,〈2,1,3,4〉,〈1,3,4,2〉,〈4,2,1,3〉〉
513: 〈〈3,4,2,1〉,〈2,1,3,4〉,〈4,2,1,3〉,〈1,3,4,2〉〉, 514: 〈〈3,4,2,1〉,〈2,1,3,4〉,〈4,3,1,2〉,〈1,2,4,3〉〉
515: 〈〈3,4,2,1〉,〈2,1,4,3〉,〈1,2,3,4〉,〈4,3,1,2〉〉, 516: 〈〈3,4,2,1〉,〈2,1,4,3〉,〈4,3,1,2〉,〈1,2,3,4〉〉
517: 〈〈3,4,2,1〉,〈1,3,4,2〉,〈2,1,3,4〉,〈4,2,1,3〉〉, 518: 〈〈3,4,2,1〉,〈1,3,4,2〉,〈4,2,1,3〉,〈2,1,3,4〉〉
519: 〈〈3,4,2,1〉,〈4,1,3,2〉,〈1,2,4,3〉,〈2,3,1,4〉〉, 520: 〈〈3,4,2,1〉,〈4,1,3,2〉,〈2,3,1,4〉,〈1,2,4,3〉〉
521: 〈〈3,4,2,1〉,〈2,3,1,4〉,〈1,2,4,3〉,〈4,1,3,2〉〉, 522: 〈〈3,4,2,1〉,〈2,3,1,4〉,〈4,1,3,2〉,〈1,2,4,3〉〉
523: 〈〈3,4,2,1〉,〈4,2,1,3〉,〈2,1,3,4〉,〈1,3,4,2〉〉, 524: 〈〈3,4,2,1〉,〈4,2,1,3〉,〈1,3,4,2〉,〈2,1,3,4〉〉
525: 〈〈3,4,2,1〉,〈4,3,1,2〉,〈1,2,3,4〉,〈2,1,4,3〉〉, 526: 〈〈3,4,2,1〉,〈4,3,1,2〉,〈1,2,4,3〉,〈2,1,3,4〉〉
527: 〈〈3,4,2,1〉,〈4,3,1,2〉,〈2,1,3,4〉,〈1,2,4,3〉〉, 528: 〈〈3,4,2,1〉,〈4,3,1,2〉,〈2,1,4,3〉,〈1,2,3,4〉〉
529: 〈〈4,3,1,2〉,〈1,2,3,4〉,〈2,1,4,3〉,〈3,4,2,1〉〉, 530: 〈〈4,3,1,2〉,〈1,2,3,4〉,〈3,4,2,1〉,〈2,1,4,3〉〉
531: 〈〈4,3,1,2〉,〈1,2,4,3〉,〈2,1,3,4〉,〈3,4,2,1〉〉, 532: 〈〈4,3,1,2〉,〈1,2,4,3〉,〈3,1,2,4〉,〈2,4,3,1〉〉
533: 〈〈4,3,1,2〉,〈1,2,4,3〉,〈2,4,3,1〉,〈3,1,2,4〉〉, 534: 〈〈4,3,1,2〉,〈1,2,4,3〉,〈3,4,2,1〉,〈2,1,3,4〉〉
535: 〈〈4,3,1,2〉,〈2,1,3,4〉,〈1,2,4,3〉,〈3,4,2,1〉〉, 536: 〈〈4,3,1,2〉,〈2,1,3,4〉,〈1,4,2,3〉,〈3,2,4,1〉〉
537: 〈〈4,3,1,2〉,〈2,1,3,4〉,〈3,2,4,1〉,〈1,4,2,3〉〉, 538: 〈〈4,3,1,2〉,〈2,1,3,4〉,〈3,4,2,1〉,〈1,2,4,3〉〉
539: 〈〈4,3,1,2〉,〈2,1,4,3〉,〈1,2,3,4〉,〈3,4,2,1〉〉, 540: 〈〈4,3,1,2〉,〈2,1,4,3〉,〈3,4,2,1〉,〈1,2,3,4〉〉
541: 〈〈4,3,1,2〉,〈3,1,2,4〉,〈1,2,4,3〉,〈2,4,3,1〉〉, 542: 〈〈4,3,1,2〉,〈3,1,2,4〉,〈2,4,3,1〉,〈1,2,4,3〉〉
543: 〈〈4,3,1,2〉,〈1,4,2,3〉,〈2,1,3,4〉,〈3,2,4,1〉〉, 544: 〈〈4,3,1,2〉,〈1,4,2,3〉,〈3,2,4,1〉,〈2,1,3,4〉〉
545: 〈〈4,3,1,2〉,〈3,2,4,1〉,〈2,1,3,4〉,〈1,4,2,3〉〉, 546: 〈〈4,3,1,2〉,〈3,2,4,1〉,〈1,4,2,3〉,〈2,1,3,4〉〉
547: 〈〈4,3,1,2〉,〈2,4,3,1〉,〈1,2,4,3〉,〈3,1,2,4〉〉, 548: 〈〈4,3,1,2〉,〈2,4,3,1〉,〈3,1,2,4〉,〈1,2,4,3〉〉
549: 〈〈4,3,1,2〉,〈3,4,2,1〉,〈1,2,3,4〉,〈2,1,4,3〉〉, 550: 〈〈4,3,1,2〉,〈3,4,2,1〉,〈1,2,4,3〉,〈2,1,3,4〉〉
551: 〈〈4,3,1,2〉,〈3,4,2,1〉,〈2,1,3,4〉,〈1,2,4,3〉〉, 552: 〈〈4,3,1,2〉,〈3,4,2,1〉,〈2,1,4,3〉,〈1,2,3,4〉〉
553: 〈〈4,3,2,1〉,〈1,2,3,4〉,〈2,1,4,3〉,〈3,4,1,2〉〉, 554: 〈〈4,3,2,1〉,〈1,2,3,4〉,〈3,1,4,2〉,〈2,4,1,3〉〉
555: 〈〈4,3,2,1〉,〈1,2,3,4〉,〈2,4,1,3〉,〈3,1,4,2〉〉, 556: 〈〈4,3,2,1〉,〈1,2,3,4〉,〈3,4,1,2〉,〈2,1,4,3〉〉
557: 〈〈4,3,2,1〉,〈1,2,4,3〉,〈2,1,3,4〉,〈3,4,1,2〉〉, 558: 〈〈4,3,2,1〉,〈1,2,4,3〉,〈3,4,1,2〉,〈2,1,3,4〉〉
559: 〈〈4,3,2,1〉,〈2,1,3,4〉,〈1,2,4,3〉,〈3,4,1,2〉〉, 560: 〈〈4,3,2,1〉,〈2,1,3,4〉,〈3,4,1,2〉,〈1,2,4,3〉〉
561: 〈〈4,3,2,1〉,〈2,1,4,3〉,〈1,2,3,4〉,〈3,4,1,2〉〉, 562: 〈〈4,3,2,1〉,〈2,1,4,3〉,〈1,4,3,2〉,〈3,2,1,4〉〉
563: 〈〈4,3,2,1〉,〈2,1,4,3〉,〈3,2,1,4〉,〈1,4,3,2〉〉, 564: 〈〈4,3,2,1〉,〈2,1,4,3〉,〈3,4,1,2〉,〈1,2,3,4〉〉
565: 〈〈4,3,2,1〉,〈3,1,4,2〉,〈1,2,3,4〉,〈2,4,1,3〉〉, 566: 〈〈4,3,2,1〉,〈3,1,4,2〉,〈2,4,1,3〉,〈1,2,3,4〉〉
567: 〈〈4,3,2,1〉,〈1,4,3,2〉,〈2,1,4,3〉,〈3,2,1,4〉〉, 568: 〈〈4,3,2,1〉,〈1,4,3,2〉,〈3,2,1,4〉,〈2,1,4,3〉〉
569: 〈〈4,3,2,1〉,〈3,2,1,4〉,〈2,1,4,3〉,〈1,4,3,2〉〉, 570: 〈〈4,3,2,1〉,〈3,2,1,4〉,〈1,4,3,2〉,〈2,1,4,3〉〉
571: 〈〈4,3,2,1〉,〈2,4,1,3〉,〈1,2,3,4〉,〈3,1,4,2〉〉, 572: 〈〈4,3,2,1〉,〈2,4,1,3〉,〈3,1,4,2〉,〈1,2,3,4〉〉
573: 〈〈4,3,2,1〉,〈3,4,1,2〉,〈1,2,3,4〉,〈2,1,4,3〉〉, 574: 〈〈4,3,2,1〉,〈3,4,1,2〉,〈1,2,4,3〉,〈2,1,3,4〉〉
575: 〈〈4,3,2,1〉,〈3,4,1,2〉,〈2,1,3,4〉,〈1,2,4,3〉〉, 576: 〈〈4,3,2,1〉,〈3,4,1,2〉,〈2,1,4,3〉,〈1,2,3,4〉〉

## Appendix B. Invalid Runs (In Decimal and Binary Forms)

Decimal and binary representations of each state are separated by “:”. Consecutive states of each run are separated by “,”. Consecutive runs are separated by |.

580: 1001000100 | 608: 1001100000 | 585: 1001001001, 868: 1101100100 | 834: 1101000010 | 656: 1010010000 | 596: 1001010100 | 706: 1011000010 | 752: 1011110000 | 599: 1001010111, 875: 1101101011, 1013: 1111110101, 954: 1110111010 | 686: 1010101110 | 747: 1011101011, 821: 1100110101, 986: 1111011010 | 694: 1010110110 | 749: 1011101101, 822: 1100110110 | 653: 1010001101, 774: 1100000110 | 641: 1010000001, 768: 1100000000 | 577: 1001000001, 864: 1101100000 | 589: 1001001101, 870: 1101100110 | 665: 1010011001, 780: 1100001100 | 848: 1101010000 | 602: 1001011010 | 726: 1011010110 | 757: 1011110101, 826: 1100111010 | 654: 1010001110 | 739: 1011100011, 817: 1100110001, 984: 1111011000 | 637: 1001111101, 894: 1101111110 | 671: 1010011111, 783: 1100001111, 967: 1111000111, 931: 1110100011, 913: 1110010001, 904: 1110001000 | 632: 1001111000 | 615: 1001100111, 883: 1101110011, 1017: 1111111001, 956: 1110111100 | 859: 1101011011, 1005: 1111101101, 950: 1110110110 685: 1010101101, 790: 1100010110 | 645: 1010000101, 770: 1100000010 | 640: 1010000000 | 578: 1001000010 | 720: 1011010000 | 598: 1001010110 | 725: 1011010101, 810: 1100101010 | 650: 1010001010 | 738: 1011100010 | 760: 1011111000 | 623: 1001101111, 887: 1101110111, 1019: 1111111011, 957: 1110111101, 926: 1110011110 | 679: 1010100111, 787: 1100010011, 969: 1111001001, 932: 1110100100 | 705: 1011000001, 800: 1100100000 | 588: 1001001100 | 836: 1101000100 | 610: 1001100010 | 728: 1011011000 | 621: 1001101101, 886: 1101110110 | 669: 1010011101, 782: 1100001110 | 643: 1010000011, 769: 1100000001, 960: 1111000000 | 583: 1001000111, 867: 1101100011, 1009: 1111110001, 952: 1110111000 | 635: 1001111011, 893: 1101111101, 1022: 1111111110 | 703: 1010111111, 799: 1100011111, 975: 1111001111, 935: 1110100111, 915: 1110010011, 905: 1110001001, 900: 1110000100 | 593: 1001010001, 872: 1101101000 | 630: 1001110110 | 733: 1011011101, 814: 1100101110 | 651: 1010001011, 773: 1100000101, 962: 1111000010 | 688: 1010110000 | 597: 1001010101, 874: 1101101010 | 666: 1010011010 | 742: 1011100110 | 761: 1011111001, 828: 1100111100 | 851: 1101010011, 1001: 1111101001, 948: 1110110100 | 717: 1011001101, 806: 1100100110 | 649: 1010001001, 772: 1100000100 | 584: 1001001000 | 612: 1001100100 | 832: 1101000000 | 582: 1001000110 | 721: 1011010001, 808: 1100101000 | 626: 1001110010, 732: 1011011100 | 845: 1101001101, 998: 1111100110 | 697: 1010111001, 796: 1100011100 | 849: 1101010001, 1000: 1111101000 | 638: 1001111110 | 735: 1011011111, 815: 1100101111, 983: 1111010111, 939: 1110101011, 917: 1110010101, 906: 1110001010 | 674: 1010100010 | 744: 1011101000 | 622: 1001101110 | 731: 1011011011, 813: 1100101101, 982: 1111010110 | 693: 1010110101, 794: 1100011010 | 646: 1010000110 | 737: 1011100001, 816: 1100110000 | 601: 1001011001, 876: 1101101100 | 854: 1101010110 | 661: 1010010101, 778: 1100001010 | 642: 1010000010 | 736: 1011100000 | 587: 1001001011, 869: 1101100101, 1010: 1111110010 | 700: 1010111100 | 843: 1101001011, 997: 1111100101, 946: 1110110010 | 684: 1010101100 | 842: 1101001010 | 658: 1010010010 | 740: 1011100100 | 833: 1101000001, 992: 1111100000 | 591: 1001001111, 871: 1101100111, 1011: 1111110011, 953: 1110111001, 924: 1110011100 | 857: 1101011001, 1004: 1111101100 | 862: 1101011110 | 663: 1010010111, 779: 1100001011, 965: 1111000101, 930: 1110100010 | 680: 1010101000 | 618: 1001101010 | 730: 1011011010 | 758: 1011110110 | 765: 1011111101, 830: 1100111110 | 655: 1010001111, 775: 1100000111, 963: 1111000011, 929: 1110100001, 912: 1110010000 | 604: 1001011100 | 837: 1101000101, 994: 1111100010 | 696: 1010111000 | 619: 1001101011, 885: 1101110101, 1018: 1111111010 | 702: 1010111110 | 751: 1011101111, 823: 1100110111, 987: 1111011011, 941: 1110101101, 918: 1110010110 | 677: 1010100101, 786: 1100010010 | 644: 1010000100 | 592: 1001010000 | 594: 1001010010 | 724: 1011010100 | 710: 1011000110 | 753: 1011110001, 824: 1100111000 | 627: 1001110011, 889: 1101111001, 1020: 1111111100 | 863: 1101011111, 1007: 1111101111, 951: 1110110111, 923: 1110011011, 909: 1110001101, 902: 1110000110 | 673: 1010100001, 784: 1100010000 | 600: 1001011000 | 613: 1001100101, 882: 1101110010 | 668: 1010011100 | 841: 1101001001, 996: 1111100100 | 835: 1101000011, 993: 1111100001, 944: 1110110000 | 605: 1001011101, 878: 1101101110 | 667: 1010011011, 781: 1100001101, 966: 1111000110 | 689: 1010110001, 792: 1100011000 | 625: 1001110001, 888: 1101111000 | 631: 1001110111, 891: 1101111011, 1021: 1111111101, 958: 1110111110 | 687: 1010101111, 791: 1100010111, 971: 1111001011, 933: 1110100101, 914: 1110010010 | 676: 1010100100 | 704: 1011000000 | 581: 1001000101, 866: 1101100010 | 664: 1010011000 | 617: 1001101001, 884: 1101110100 | 715: 1011001011, 805: 1100100101, 978: 1111010010 | 692: 1010110100 | 709: 1011000101, 802: 1100100010 | 648: 1010001000 | 616: 1001101000 | 614: 1001100110 | 729: 1011011001, 812: 1100101100 | 850: 1101010010 | 660: 1010010100 | 708: 1011000100 | 609: 1001100001, 880: 1101110000 | 603: 1001011011, 877: 1101101101, 1014: 1111110110 | 701: 1010111101, 798: 1100011110 | 647: 1010000111, 771: 1100000011, 961: 1111000001, 928: 1110100000 | 590: 1001001110 | 723: 1011010011, 809: 1100101001, 980: 1111010100 | 718: 1011001110 | 755: 1011110011, 825: 1100111001, 988: 1111011100 | 861: 1101011101, 1006: 1111101110 | 699: 1010111011, 797: 1100011101, 974: 1111001110 | 691: 1010110011, 793: 1100011001, 972: 1111001100 | 860: 1101011100 | 853: 1101010101, 1002: 1111101010 | 698: 1010111010 | 750: 1011101110 | 763: 1011111011, 829: 1100111101, 990: 1111011110 | 695: 1010110111, 795: 1100011011, 973: 1111001101, 934: 1110100110 | 681: 1010101001, 788: 1100010100 | 712: 1011001000 | 620: 1001101100 | 838: 1101000110 | 657: 1010010001, 776: 1100001000 | 624: 1001110000 | 595: 1001010011, 873: 1101101001, 1012: 1111110100 | 719: 1011001111, 807: 1100100111, 979: 1111010011, 937: 1110101001, 916: 1110010100 | 716: 1011001100 | 844: 1101001100 | 852: 1101010100 | 714: 1011001010 | 754: 1011110010 | 764: 1011111100 | 847: 1101001111, 999: 1111100111, 947: 1110110011, 921: 1110011001, 908: 1110001100 | 856: 1101011000 | 629: 1001110101, 890: 1101111010 | 670: 1010011110 | 743: 1011100111, 819: 1100110011, 985: 1111011001, 940: 1110101100 | 858: 1101011010 | 662: 1010010110 | 741: 1011100101, 818: 1100110010 | 652: 1010001100 | 840: 1101001000 | 628: 1001110100 | 707: 1011000011, 801: 1100100001, 976: 1111010000 | 606: 1001011110 | 727: 1011010111, 811: 1100101011, 981: 1111010101, 938: 1110101010 | 682: 1010101010 | 746: 1011101010 | 762: 1011111010 | 766: 1011111110 | 767: 1011111111, 831: 1100111111, 991: 1111011111, 943: 1110101111, 919: 1110010111, 907: 1110001011, 901: 1110000101, 898: 1110000010 | 672: 1010100000 | 586: 1001001010 | 722: 1011010010 | 756: 1011110100 | 711: 1011000111, 803: 1100100011, 977: 1111010001, 936: 1110101000 | 634: 1001111010 | 734: 1011011110 | 759: 1011110111, 827: 1100111011, 989: 1111011101, 942: 1110101110 | 683: 1010101011, 789: 1100010101, 970: 1111001010 | 690: 1010110010 | 748: 1011101100 | 846: 1101001110 | 659: 1010010011, 777: 1100001001, 964: 1111000100 | 611: 1001100011, 881: 1101110001, 1016: 1111111000 | 639: 1001111111, 895: 1101111111, 1023: 1111111111, 959: 1110111111, 927: 1110011111, 911: 1110001111, 903: 1110000111, 899: 1110000011, 897: 1110000001, 896: 1110000000 | 579: 1001000011, 865: 1101100001, 1008: 1111110000 | 607: 1001011111, 879: 1101101111, 1015: 1111110111, 955: 1110111011, 925: 1110011101, 910: 1110001110 | 675: 1010100011, 785: 1100010001, 968: 1111001000 | 636: 1001111100 | 839: 1101000111, 995: 1111100011, 945: 1110110001, 920: 1110011000 | 633: 1001111001, 892: 1101111100 | 855: 1101010111, 1003: 1111101011, 949: 1110110101, 922: 1110011010 | 678: 1010100110 | 745: 1011101001, 820: 1100110100 | 713: 1011001001, 804: 1100100100 |

## Appendix C. Enablers

For i∈{1,2,…,10}, `$F_{i}\left(0\right)$’ has been replaced by `$F_{i}$’ for simplicity.

****************************************************

1-Run Beginnings (2-Parallel State Transition):

****************************************************

$F_{1}F_{2}^{\prime }F_{3}^{\prime }F_{4}F_{5}^{\prime }F_{6}^{\prime }F_{7}^{\prime }F_{8}F_{9}^{\prime }F_{10}^{\prime }\;†\;F_{1}F_{2}^{\prime }F_{3}^{\prime }F_{4}F_{5}F_{6}^{\prime }F_{7}^{\prime }F_{8}^{\prime }F_{9}^{\prime }F_{10}^{\prime }\;†\;F_{1}F_{2}F_{3}^{\prime }F_{4}F_{5}^{\prime }F_{6}^{\prime }F_{7}^{\prime }F_{8}^{\prime }F_{9}F_{10}^{\prime }\;†\;F_{1}F_{2}^{\prime }F_{3}F_{4}^{\prime }F_{5}^{\prime }F_{6}F_{7}^{\prime }F_{8}^{\prime }F_{9}^{\prime }F_{10}^{\prime }\;†$
$F_{1}F_{2}^{\prime }F_{3}^{\prime }F_{4}F_{5}^{\prime }F_{6}F_{7}^{\prime }F_{8}F_{9}^{\prime }F_{10}^{\prime }\;†\;F_{1}F_{2}^{\prime }F_{3}F_{4}F_{5}^{\prime }F_{6}^{\prime }F_{7}^{\prime }F_{8}^{\prime }F_{9}F_{10}^{\prime }\;†\;F_{1}F_{2}^{\prime }F_{3}F_{4}F_{5}F_{6}F_{7}^{\prime }F_{8}^{\prime }F_{9}^{\prime }F_{10}^{\prime }\;†\;F_{1}F_{2}^{\prime }F_{3}F_{4}^{\prime }F_{5}F_{6}^{\prime }F_{7}F_{8}F_{9}F_{10}^{\prime }\;†$
$F_{1}F_{2}^{\prime }F_{3}F_{4}^{\prime }F_{5}F_{6}F_{7}^{\prime }F_{8}F_{9}F_{10}^{\prime }\;†\;F_{1}F_{2}F_{3}^{\prime }F_{4}F_{5}^{\prime }F_{6}F_{7}^{\prime }F_{8}^{\prime }F_{9}^{\prime }F_{10}^{\prime }\;†\;F_{1}F_{2}^{\prime }F_{3}^{\prime }F_{4}F_{5}^{\prime }F_{6}F_{7}F_{8}^{\prime }F_{9}F_{10}^{\prime }\;†\;F_{1}F_{2}^{\prime }F_{3}F_{4}F_{5}^{\prime }F_{6}F_{7}^{\prime }F_{8}F_{9}F_{10}^{\prime }\;†$
$F_{1}F_{2}^{\prime }F_{3}F_{4}^{\prime }F_{5}^{\prime }F_{6}^{\prime }F_{7}F_{8}F_{9}F_{10}^{\prime }\;†\;F_{1}F_{2}^{\prime }F_{3}^{\prime }F_{4}F_{5}F_{6}F_{7}F_{8}^{\prime }F_{9}^{\prime }F_{10}^{\prime }\;†\;F_{1}F_{2}^{\prime }F_{3}F_{4}^{\prime }F_{5}^{\prime }F_{6}^{\prime }F_{7}^{\prime }F_{8}^{\prime }F_{9}^{\prime }F_{10}^{\prime }\;†\;F_{1}F_{2}^{\prime }F_{3}^{\prime }F_{4}F_{5}^{\prime }F_{6}^{\prime }F_{7}^{\prime }F_{8}^{\prime }F_{9}F_{10}^{\prime }\;†$
$F_{1}F_{2}^{\prime }F_{3}F_{4}F_{5}^{\prime }F_{6}F_{7}^{\prime }F_{8}^{\prime }F_{9}^{\prime }F_{10}^{\prime }\;†\;F_{1}F_{2}^{\prime }F_{3}^{\prime }F_{4}F_{5}^{\prime }F_{6}F_{7}^{\prime }F_{8}F_{9}F_{10}^{\prime }\;†\;F_{1}F_{2}^{\prime }F_{3}F_{4}^{\prime }F_{5}^{\prime }F_{6}^{\prime }F_{7}F_{8}^{\prime }F_{9}F_{10}^{\prime }\;†\;F_{1}F_{2}^{\prime }F_{3}F_{4}F_{5}F_{6}^{\prime }F_{7}^{\prime }F_{8}^{\prime }F_{9}F_{10}^{\prime }\;†$
$F_{1}F_{2}^{\prime }F_{3}F_{4}F_{5}F_{6}F_{7}F_{8}^{\prime }F_{9}^{\prime }F_{10}^{\prime }\;†\;F_{1}F_{2}^{\prime }F_{3}^{\prime }F_{4}F_{5}^{\prime }F_{6}^{\prime }F_{7}F_{8}F_{9}^{\prime }F_{10}^{\prime }\;†\;F_{1}F_{2}F_{3}^{\prime }F_{4}F_{5}^{\prime }F_{6}^{\prime }F_{7}^{\prime }F_{8}F_{9}^{\prime }F_{10}^{\prime }\;†\;F_{1}F_{2}^{\prime }F_{3}^{\prime }F_{4}F_{5}F_{6}^{\prime }F_{7}^{\prime }F_{8}^{\prime }F_{9}F_{10}^{\prime }\;†$
$F_{1}F_{2}^{\prime }F_{3}F_{4}F_{5}^{\prime }F_{6}F_{7}F_{8}^{\prime }F_{9}^{\prime }F_{10}^{\prime }\;†\;F_{1}F_{2}^{\prime }F_{3}^{\prime }F_{4}F_{5}F_{6}F_{7}^{\prime }F_{8}F_{9}F_{10}^{\prime }\;†\;F_{1}F_{2}^{\prime }F_{3}F_{4}^{\prime }F_{5}F_{6}F_{7}^{\prime }F_{8}^{\prime }F_{9}^{\prime }F_{10}^{\prime }\;†\;F_{1}F_{2}^{\prime }F_{3}F_{4}^{\prime }F_{5}^{\prime }F_{6}F_{7}F_{8}^{\prime }F_{9}F_{10}^{\prime }\;†$
$F_{1}F_{2}^{\prime }F_{3}F_{4}F_{5}F_{6}^{\prime }F_{7}^{\prime }F_{8}F_{9}F_{10}^{\prime }\;†\;F_{1}F_{2}^{\prime }F_{3}^{\prime }F_{4}F_{5}^{\prime }F_{6}^{\prime }F_{7}F_{8}^{\prime }F_{9}^{\prime }F_{10}^{\prime }\;†\;F_{1}F_{2}^{\prime }F_{3}^{\prime }F_{4}F_{5}F_{6}^{\prime }F_{7}^{\prime }F_{8}F_{9}^{\prime }F_{10}^{\prime }\;†\;F_{1}F_{2}F_{3}^{\prime }F_{4}F_{5}^{\prime }F_{6}^{\prime }F_{7}^{\prime }F_{8}^{\prime }F_{9}^{\prime }F_{10}^{\prime }\;†$
$F_{1}F_{2}^{\prime }F_{3}^{\prime }F_{4}F_{5}^{\prime }F_{6}^{\prime }F_{7}^{\prime }F_{8}F_{9}F_{10}^{\prime }\;†\;F_{1}F_{2}^{\prime }F_{3}^{\prime }F_{4}F_{5}F_{6}F_{7}^{\prime }F_{8}^{\prime }F_{9}F_{10}^{\prime }\;†\;F_{1}F_{2}^{\prime }F_{3}F_{4}F_{5}^{\prime }F_{6}F_{7}F_{8}F_{9}^{\prime }F_{10}^{\prime }\;†\;F_{1}F_{2}^{\prime }F_{3}^{\prime }F_{4}F_{5}F_{6}F_{7}F_{8}F_{9}F_{10}^{\prime }\;†$
$F_{1}F_{2}^{\prime }F_{3}F_{4}^{\prime }F_{5}F_{6}^{\prime }F_{7}^{\prime }F_{8}^{\prime }F_{9}F_{10}^{\prime }\;†\;F_{1}F_{2}^{\prime }F_{3}F_{4}F_{5}F_{6}^{\prime }F_{7}F_{8}^{\prime }F_{9}^{\prime }F_{10}^{\prime }\;†\;F_{1}F_{2}^{\prime }F_{3}^{\prime }F_{4}F_{5}F_{6}^{\prime }F_{7}F_{8}F_{9}F_{10}^{\prime }\;†\;F_{1}F_{2}^{\prime }F_{3}F_{4}^{\prime }F_{5}^{\prime }F_{6}^{\prime }F_{7}^{\prime }F_{8}F_{9}F_{10}^{\prime }\;†$
$F_{1}F_{2}F_{3}^{\prime }F_{4}F_{5}^{\prime }F_{6}F_{7}^{\prime }F_{8}F_{9}F_{10}^{\prime }\;†\;F_{1}F_{2}^{\prime }F_{3}F_{4}^{\prime }F_{5}^{\prime }F_{6}^{\prime }F_{7}^{\prime }F_{8}^{\prime }F_{9}F_{10}^{\prime }\;†\;F_{1}F_{2}^{\prime }F_{3}F_{4}F_{5}F_{6}^{\prime }F_{7}^{\prime }F_{8}^{\prime }F_{9}^{\prime }F_{10}^{\prime }\;†\;F_{1}F_{2}^{\prime }F_{3}F_{4}^{\prime }F_{5}F_{6}F_{7}F_{8}F_{9}^{\prime }F_{10}^{\prime }\;†$
$F_{1}F_{2}^{\prime }F_{3}F_{4}^{\prime }F_{5}F_{6}^{\prime }F_{7}F_{8}F_{9}^{\prime }F_{10}^{\prime }\;†\;F_{1}F_{2}F_{3}^{\prime }F_{4}F_{5}^{\prime }F_{6}^{\prime }F_{7}F_{8}^{\prime }F_{9}F_{10}^{\prime }\;†\;F_{1}F_{2}^{\prime }F_{3}F_{4}^{\prime }F_{5}^{\prime }F_{6}F_{7}^{\prime }F_{8}^{\prime }F_{9}F_{10}^{\prime }\;†\;F_{1}F_{2}^{\prime }F_{3}F_{4}F_{5}F_{6}^{\prime }F_{7}^{\prime }F_{8}F_{9}^{\prime }F_{10}^{\prime }\;†$
$F_{1}F_{2}F_{3}^{\prime }F_{4}F_{5}^{\prime }F_{6}F_{7}F_{8}F_{9}F_{10}^{\prime }\;†\;F_{1}F_{2}^{\prime }F_{3}F_{4}^{\prime }F_{5}F_{6}^{\prime }F_{7}F_{8}^{\prime }F_{9}^{\prime }F_{10}^{\prime }\;†\;F_{1}F_{2}^{\prime }F_{3}^{\prime }F_{4}F_{5}F_{6}^{\prime }F_{7}F_{8}^{\prime }F_{9}F_{10}^{\prime }\;†\;F_{1}F_{2}^{\prime }F_{3}F_{4}F_{5}^{\prime }F_{6}F_{7}F_{8}^{\prime }F_{9}F_{10}^{\prime }\;†$
$F_{1}F_{2}^{\prime }F_{3}F_{4}F_{5}F_{6}F_{7}^{\prime }F_{8}F_{9}F_{10}^{\prime }\;†\;F_{1}F_{2}^{\prime }F_{3}^{\prime }F_{4}F_{5}^{\prime }F_{6}F_{7}F_{8}F_{9}^{\prime }F_{10}^{\prime }\;†\;F_{1}F_{2}^{\prime }F_{3}F_{4}^{\prime }F_{5}F_{6}F_{7}F_{8}^{\prime }F_{9}^{\prime }F_{10}^{\prime }\;†\;F_{1}F_{2}^{\prime }F_{3}F_{4}^{\prime }F_{5}F_{6}F_{7}F_{8}F_{9}F_{10}^{\prime }\;†$
$F_{1}F_{2}^{\prime }F_{3}F_{4}^{\prime }F_{5}^{\prime }F_{6}^{\prime }F_{7}^{\prime }F_{8}F_{9}^{\prime }F_{10}^{\prime }\;†\;F_{1}F_{2}^{\prime }F_{3}^{\prime }F_{4}F_{5}^{\prime }F_{6}F_{7}^{\prime }F_{8}^{\prime }F_{9}^{\prime }F_{10}^{\prime }\;†\;F_{1}F_{2}^{\prime }F_{3}^{\prime }F_{4}F_{5}^{\prime }F_{6}F_{7}^{\prime }F_{8}^{\prime }F_{9}F_{10}^{\prime }\;†\;F_{1}F_{2}^{\prime }F_{3}F_{4}F_{5}^{\prime }F_{6}F_{7}^{\prime }F_{8}F_{9}^{\prime }F_{10}^{\prime }\;†$
$F_{1}F_{2}^{\prime }F_{3}F_{4}F_{5}^{\prime }F_{6}^{\prime }F_{7}^{\prime }F_{8}F_{9}F_{10}^{\prime }\;†\;F_{1}F_{2}^{\prime }F_{3}^{\prime }F_{4}F_{5}^{\prime }F_{6}F_{7}F_{8}^{\prime }F_{9}^{\prime }F_{10}^{\prime }\;†\;F_{1}F_{2}^{\prime }F_{3}F_{4}^{\prime }F_{5}^{\prime }F_{6}F_{7}F_{8}F_{9}^{\prime }F_{10}^{\prime }\;†\;F_{1}F_{2}^{\prime }F_{3}F_{4}^{\prime }F_{5}F_{6}^{\prime }F_{7}^{\prime }F_{8}F_{9}^{\prime }F_{10}^{\prime }\;†$
$F_{1}F_{2}^{\prime }F_{3}F_{4}F_{5}^{\prime }F_{6}^{\prime }F_{7}^{\prime }F_{8}^{\prime }F_{9}^{\prime }F_{10}^{\prime }\;†\;F_{1}F_{2}^{\prime }F_{3}F_{4}^{\prime }F_{5}^{\prime }F_{6}F_{7}F_{8}^{\prime }F_{9}^{\prime }F_{10}^{\prime }\;†\;F_{1}F_{2}^{\prime }F_{3}F_{4}^{\prime }F_{5}F_{6}F_{7}^{\prime }F_{8}F_{9}^{\prime }F_{10}^{\prime }\;†\;F_{1}F_{2}^{\prime }F_{3}F_{4}^{\prime }F_{5}^{\prime }F_{6}^{\prime }F_{7}F_{8}^{\prime }F_{9}^{\prime }F_{10}^{\prime }\;†$
$F_{1}F_{2}^{\prime }F_{3}^{\prime }F_{4}F_{5}F_{6}^{\prime }F_{7}F_{8}^{\prime }F_{9}^{\prime }F_{10}^{\prime }\;†\;F_{1}F_{2}^{\prime }F_{3}^{\prime }F_{4}F_{5}F_{6}^{\prime }F_{7}^{\prime }F_{8}F_{9}F_{10}^{\prime }\;†\;F_{1}F_{2}F_{3}^{\prime }F_{4}F_{5}^{\prime }F_{6}F_{7}^{\prime }F_{8}^{\prime }F_{9}F_{10}^{\prime }\;†\;F_{1}F_{2}^{\prime }F_{3}F_{4}^{\prime }F_{5}^{\prime }F_{6}F_{7}^{\prime }F_{8}F_{9}^{\prime }F_{10}^{\prime }\;†$
$F_{1}F_{2}^{\prime }F_{3}F_{4}F_{5}^{\prime }F_{6}^{\prime }F_{7}^{\prime }F_{8}F_{9}^{\prime }F_{10}^{\prime }\;†\;F_{1}F_{2}^{\prime }F_{3}^{\prime }F_{4}F_{5}^{\prime }F_{6}^{\prime }F_{7}F_{8}F_{9}F_{10}^{\prime }\;†\;F_{1}F_{2}^{\prime }F_{3}F_{4}F_{5}^{\prime }F_{6}^{\prime }F_{7}F_{8}F_{9}F_{10}^{\prime }\;†\;F_{1}F_{2}F_{3}^{\prime }F_{4}F_{5}^{\prime }F_{6}F_{7}F_{8}F_{9}^{\prime }F_{10}^{\prime }\;†$
$F_{1}F_{2}^{\prime }F_{3}F_{4}^{\prime }F_{5}F_{6}F_{7}F_{8}^{\prime }F_{9}F_{10}^{\prime }\;†\;F_{1}F_{2}^{\prime }F_{3}F_{4}F_{5}F_{6}^{\prime }F_{7}F_{8}F_{9}F_{10}^{\prime }\;†\;F_{1}F_{2}^{\prime }F_{3}F_{4}F_{5}^{\prime }F_{6}^{\prime }F_{7}F_{8}^{\prime }F_{9}^{\prime }F_{10}^{\prime }\;†\;F_{1}F_{2}^{\prime }F_{3}^{\prime }F_{4}F_{5}F_{6}^{\prime }F_{7}F_{8}F_{9}^{\prime }F_{10}^{\prime }\;†$
$F_{1}F_{2}F_{3}^{\prime }F_{4}F_{5}^{\prime }F_{6}^{\prime }F_{7}^{\prime }F_{8}F_{9}F_{10}^{\prime }\;†\;F_{1}F_{2}^{\prime }F_{3}^{\prime }F_{4}F_{5}F_{6}F_{7}^{\prime }F_{8}^{\prime }F_{9}^{\prime }F_{10}^{\prime }\;†\;F_{1}F_{2}^{\prime }F_{3}F_{4}F_{5}^{\prime }F_{6}^{\prime }F_{7}F_{8}F_{9}^{\prime }F_{10}^{\prime }\;†\;F_{1}F_{2}F_{3}^{\prime }F_{4}F_{5}^{\prime }F_{6}^{\prime }F_{7}F_{8}F_{9}^{\prime }F_{10}^{\prime }\;†$
$F_{1}F_{2}F_{3}^{\prime }F_{4}F_{5}^{\prime }F_{6}F_{7}^{\prime }F_{8}F_{9}^{\prime }F_{10}^{\prime }\;†\;F_{1}F_{2}^{\prime }F_{3}F_{4}F_{5}^{\prime }F_{6}^{\prime }F_{7}F_{8}^{\prime }F_{9}F_{10}^{\prime }\;†\;F_{1}F_{2}^{\prime }F_{3}F_{4}F_{5}F_{6}F_{7}^{\prime }F_{8}^{\prime }F_{9}F_{10}^{\prime }\;†\;F_{1}F_{2}^{\prime }F_{3}F_{4}F_{5}F_{6}F_{7}F_{8}F_{9}^{\prime }F_{10}^{\prime }\;†$
$F_{1}F_{2}F_{3}^{\prime }F_{4}F_{5}^{\prime }F_{6}F_{7}F_{8}^{\prime }F_{9}^{\prime }F_{10}^{\prime }\;†\;F_{1}F_{2}^{\prime }F_{3}F_{4}^{\prime }F_{5}^{\prime }F_{6}F_{7}F_{8}F_{9}F_{10}^{\prime }\;†\;F_{1}F_{2}F_{3}^{\prime }F_{4}F_{5}^{\prime }F_{6}F_{7}F_{8}^{\prime }F_{9}F_{10}^{\prime }\;†\;F_{1}F_{2}^{\prime }F_{3}F_{4}^{\prime }F_{5}^{\prime }F_{6}F_{7}^{\prime }F_{8}F_{9}F_{10}^{\prime }\;†$
$F_{1}F_{2}^{\prime }F_{3}F_{4}^{\prime }F_{5}^{\prime }F_{6}^{\prime }F_{7}F_{8}F_{9}^{\prime }F_{10}^{\prime }\;†\;F_{1}F_{2}F_{3}^{\prime }F_{4}F_{5}^{\prime }F_{6}^{\prime }F_{7}F_{8}^{\prime }F_{9}^{\prime }F_{10}^{\prime }\;†\;F_{1}F_{2}^{\prime }F_{3}^{\prime }F_{4}F_{5}F_{6}F_{7}^{\prime }F_{8}F_{9}^{\prime }F_{10}^{\prime }\;†\;F_{1}F_{2}^{\prime }F_{3}^{\prime }F_{4}F_{5}^{\prime }F_{6}F_{7}F_{8}F_{9}F_{10}^{\prime }\;†$
$F_{1}F_{2}^{\prime }F_{3}F_{4}^{\prime }F_{5}F_{6}^{\prime }F_{7}F_{8}^{\prime }F_{9}F_{10}^{\prime }\;†\;F_{1}F_{2}^{\prime }F_{3}F_{4}F_{5}F_{6}^{\prime }F_{7}F_{8}^{\prime }F_{9}F_{10}^{\prime }\;†\;F_{1}F_{2}^{\prime }F_{3}F_{4}F_{5}F_{6}F_{7}F_{8}^{\prime }F_{9}F_{10}^{\prime }\;†\;F_{1}F_{2}^{\prime }F_{3}F_{4}F_{5}F_{6}F_{7}F_{8}F_{9}F_{10}^{\prime }\;†$
$F_{1}F_{2}^{\prime }F_{3}F_{4}^{\prime }F_{5}F_{6}^{\prime }F_{7}^{\prime }F_{8}^{\prime }F_{9}^{\prime }F_{10}^{\prime }\;†\;F_{1}F_{2}^{\prime }F_{3}^{\prime }F_{4}F_{5}^{\prime }F_{6}^{\prime }F_{7}F_{8}^{\prime }F_{9}F_{10}^{\prime }\;†\;F_{1}F_{2}^{\prime }F_{3}F_{4}F_{5}^{\prime }F_{6}F_{7}^{\prime }F_{8}^{\prime }F_{9}F_{10}^{\prime }\;†\;F_{1}F_{2}^{\prime }F_{3}F_{4}F_{5}F_{6}F_{7}^{\prime }F_{8}F_{9}^{\prime }F_{10}^{\prime }\;†$
$F_{1}F_{2}^{\prime }F_{3}^{\prime }F_{4}F_{5}F_{6}F_{7}F_{8}^{\prime }F_{9}F_{10}^{\prime }\;†\;F_{1}F_{2}^{\prime }F_{3}F_{4}F_{5}^{\prime }F_{6}F_{7}F_{8}F_{9}F_{10}^{\prime }\;†\;F_{1}F_{2}^{\prime }F_{3}F_{4}^{\prime }F_{5}F_{6}F_{7}^{\prime }F_{8}^{\prime }F_{9}F_{10}^{\prime }\;†\;F_{1}F_{2}^{\prime }F_{3}F_{4}F_{5}F_{6}^{\prime }F_{7}F_{8}F_{9}^{\prime }F_{10}^{\prime }\;†$
$F_{1}F_{2}F_{3}^{\prime }F_{4}F_{5}^{\prime }F_{6}^{\prime }F_{7}F_{8}F_{9}F_{10}^{\prime }\;†\;F_{1}F_{2}^{\prime }F_{3}^{\prime }F_{4}F_{5}F_{6}F_{7}F_{8}F_{9}^{\prime }F_{10}^{\prime }\;†\;F_{1}F_{2}^{\prime }F_{3}F_{4}^{\prime }F_{5}F_{6}^{\prime }F_{7}^{\prime }F_{8}F_{9}F_{10}^{\prime }$

****************************************************

2-Run Beginnings (3-Parallel State Transition):

****************************************************

$F_{1}F_{2}^{\prime }F_{3}^{\prime }F_{4}F_{5}^{\prime }F_{6}^{\prime }F_{7}F_{8}^{\prime }F_{9}^{\prime }F_{10}\;†\;F_{1}F_{2}^{\prime }F_{3}F_{4}F_{5}F_{6}^{\prime }F_{7}F_{8}F_{9}^{\prime }F_{10}\;†\;F_{1}F_{2}^{\prime }F_{3}F_{4}^{\prime }F_{5}^{\prime }F_{6}^{\prime }F_{7}F_{8}F_{9}^{\prime }F_{10}\;†\;F_{1}F_{2}^{\prime }F_{3}F_{4}^{\prime }F_{5}^{\prime }F_{6}^{\prime }F_{7}^{\prime }F_{8}^{\prime }F_{9}^{\prime }F_{10}\;†$
$F_{1}F_{2}^{\prime }F_{3}^{\prime }F_{4}F_{5}^{\prime }F_{6}^{\prime }F_{7}^{\prime }F_{8}^{\prime }F_{9}^{\prime }F_{10}\;†\;F_{1}F_{2}^{\prime }F_{3}^{\prime }F_{4}F_{5}^{\prime }F_{6}^{\prime }F_{7}F_{8}F_{9}^{\prime }F_{10}\;†\;F_{1}F_{2}^{\prime }F_{3}F_{4}^{\prime }F_{5}^{\prime }F_{6}F_{7}F_{8}^{\prime }F_{9}^{\prime }F_{10}\;†\;F_{1}F_{2}^{\prime }F_{3}F_{4}F_{5}F_{6}F_{7}^{\prime }F_{8}F_{9}^{\prime }F_{10}\;†$
$F_{1}F_{2}^{\prime }F_{3}^{\prime }F_{4}F_{5}F_{6}F_{7}F_{8}F_{9}^{\prime }F_{10}\;†\;F_{1}F_{2}^{\prime }F_{3}F_{4}^{\prime }F_{5}F_{6}^{\prime }F_{7}F_{8}F_{9}^{\prime }F_{10}\;†\;F_{1}F_{2}^{\prime }F_{3}F_{4}^{\prime }F_{5}^{\prime }F_{6}^{\prime }F_{7}^{\prime }F_{8}F_{9}^{\prime }F_{10}\;†\;F_{1}F_{2}^{\prime }F_{3}F_{4}F_{5}^{\prime }F_{6}F_{7}^{\prime }F_{8}F_{9}^{\prime }F_{10}\;†$
$F_{1}F_{2}^{\prime }F_{3}F_{4}F_{5}^{\prime }F_{6}^{\prime }F_{7}^{\prime }F_{8}^{\prime }F_{9}^{\prime }F_{10}\;†\;F_{1}F_{2}^{\prime }F_{3}^{\prime }F_{4}F_{5}F_{6}^{\prime }F_{7}F_{8}F_{9}^{\prime }F_{10}\;†\;F_{1}F_{2}^{\prime }F_{3}F_{4}^{\prime }F_{5}^{\prime }F_{6}F_{7}F_{8}F_{9}^{\prime }F_{10}\;†\;F_{1}F_{2}^{\prime }F_{3}^{\prime }F_{4}F_{5}^{\prime }F_{6}F_{7}^{\prime }F_{8}^{\prime }F_{9}^{\prime }F_{10}\;†$
$F_{1}F_{2}^{\prime }F_{3}F_{4}F_{5}^{\prime }F_{6}F_{7}F_{8}F_{9}^{\prime }F_{10}\;†\;F_{1}F_{2}^{\prime }F_{3}^{\prime }F_{4}F_{5}^{\prime }F_{6}F_{7}^{\prime }F_{8}F_{9}^{\prime }F_{10}\;†\;F_{1}F_{2}^{\prime }F_{3}F_{4}F_{5}F_{6}F_{7}F_{8}^{\prime }F_{9}^{\prime }F_{10}\;†\;F_{1}F_{2}^{\prime }F_{3}F_{4}F_{5}^{\prime }F_{6}^{\prime }F_{7}F_{8}F_{9}^{\prime }F_{10}\;†$
$F_{1}F_{2}^{\prime }F_{3}F_{4}^{\prime }F_{5}^{\prime }F_{6}^{\prime }F_{7}F_{8}^{\prime }F_{9}^{\prime }F_{10}\;†\;F_{1}F_{2}^{\prime }F_{3}F_{4}F_{5}^{\prime }F_{6}F_{7}^{\prime }F_{8}^{\prime }F_{9}^{\prime }F_{10}\;†\;F_{1}F_{2}F_{3}^{\prime }F_{4}F_{5}^{\prime }F_{6}^{\prime }F_{7}F_{8}F_{9}^{\prime }F_{10}\;†\;F_{1}F_{2}^{\prime }F_{3}F_{4}^{\prime }F_{5}F_{6}F_{7}F_{8}^{\prime }F_{9}^{\prime }F_{10}\;†$
$F_{1}F_{2}F_{3}^{\prime }F_{4}F_{5}^{\prime }F_{6}F_{7}^{\prime }F_{8}^{\prime }F_{9}^{\prime }F_{10}\;†\;F_{1}F_{2}^{\prime }F_{3}F_{4}^{\prime }F_{5}F_{6}F_{7}^{\prime }F_{8}F_{9}^{\prime }F_{10}\;†\;F_{1}F_{2}^{\prime }F_{3}F_{4}F_{5}F_{6}^{\prime }F_{7}^{\prime }F_{8}^{\prime }F_{9}^{\prime }F_{10}\;†\;F_{1}F_{2}^{\prime }F_{3}^{\prime }F_{4}F_{5}^{\prime }F_{6}F_{7}F_{8}^{\prime }F_{9}^{\prime }F_{10}\;†$
$F_{1}F_{2}^{\prime }F_{3}F_{4}^{\prime }F_{5}^{\prime }F_{6}F_{7}^{\prime }F_{8}F_{9}^{\prime }F_{10}\;†\;F_{1}F_{2}F_{3}^{\prime }F_{4}F_{5}^{\prime }F_{6}^{\prime }F_{7}^{\prime }F_{8}^{\prime }F_{9}^{\prime }F_{10}\;†\;F_{1}F_{2}F_{3}^{\prime }F_{4}F_{5}^{\prime }F_{6}F_{7}F_{8}^{\prime }F_{9}^{\prime }F_{10}\;†\;F_{1}F_{2}^{\prime }F_{3}F_{4}F_{5}F_{6}F_{7}F_{8}F_{9}^{\prime }F_{10}\;†$
$F_{1}F_{2}F_{3}^{\prime }F_{4}F_{5}^{\prime }F_{6}^{\prime }F_{7}^{\prime }F_{8}F_{9}^{\prime }F_{10}\;†\;F_{1}F_{2}^{\prime }F_{3}F_{4}^{\prime }F_{5}F_{6}^{\prime }F_{7}^{\prime }F_{8}F_{9}^{\prime }F_{10}\;†\;F_{1}F_{2}^{\prime }F_{3}F_{4}F_{5}F_{6}F_{7}^{\prime }F_{8}^{\prime }F_{9}^{\prime }F_{10}\;†\;F_{1}F_{2}^{\prime }F_{3}F_{4}^{\prime }F_{5}F_{6}^{\prime }F_{7}^{\prime }F_{8}^{\prime }F_{9}^{\prime }F_{10}\;†$
$F_{1}F_{2}^{\prime }F_{3}^{\prime }F_{4}F_{5}F_{6}^{\prime }F_{7}^{\prime }F_{8}F_{9}^{\prime }F_{10}\;†\;F_{1}F_{2}F_{3}^{\prime }F_{4}F_{5}^{\prime }F_{6}^{\prime }F_{7}F_{8}^{\prime }F_{9}^{\prime }F_{10}\;†\;F_{1}F_{2}^{\prime }F_{3}^{\prime }F_{4}F_{5}^{\prime }F_{6}F_{7}F_{8}F_{9}^{\prime }F_{10}\;†\;F_{1}F_{2}^{\prime }F_{3}F_{4}^{\prime }F_{5}F_{6}F_{7}^{\prime }F_{8}^{\prime }F_{9}^{\prime }F_{10}\;†$
$F_{1}F_{2}^{\prime }F_{3}^{\prime }F_{4}F_{5}F_{6}F_{7}^{\prime }F_{8}^{\prime }F_{9}^{\prime }F_{10}\;†\;F_{1}F_{2}^{\prime }F_{3}^{\prime }F_{4}F_{5}^{\prime }F_{6}^{\prime }F_{7}^{\prime }F_{8}F_{9}^{\prime }F_{10}\;†\;F_{1}F_{2}^{\prime }F_{3}^{\prime }F_{4}F_{5}F_{6}^{\prime }F_{7}F_{8}^{\prime }F_{9}^{\prime }F_{10}\;†\;F_{1}F_{2}^{\prime }F_{3}F_{4}F_{5}^{\prime }F_{6}^{\prime }F_{7}^{\prime }F_{8}F_{9}^{\prime }F_{10}\;†$
$F_{1}F_{2}^{\prime }F_{3}F_{4}F_{5}^{\prime }F_{6}F_{7}F_{8}^{\prime }F_{9}^{\prime }F_{10}\;†\;F_{1}F_{2}^{\prime }F_{3}^{\prime }F_{4}F_{5}F_{6}^{\prime }F_{7}^{\prime }F_{8}^{\prime }F_{9}^{\prime }F_{10}\;†\;F_{1}F_{2}^{\prime }F_{3}F_{4}^{\prime }F_{5}F_{6}F_{7}F_{8}F_{9}^{\prime }F_{10}\;†\;F_{1}F_{2}F_{3}^{\prime }F_{4}F_{5}^{\prime }F_{6}F_{7}F_{8}F_{9}^{\prime }F_{10}\;†$
$F_{1}F_{2}F_{3}^{\prime }F_{4}F_{5}^{\prime }F_{6}F_{7}^{\prime }F_{8}F_{9}^{\prime }F_{10}\;†\;F_{1}F_{2}^{\prime }F_{3}F_{4}^{\prime }F_{5}F_{6}^{\prime }F_{7}F_{8}^{\prime }F_{9}^{\prime }F_{10}\;†\;F_{1}F_{2}^{\prime }F_{3}F_{4}^{\prime }F_{5}^{\prime }F_{6}F_{7}^{\prime }F_{8}^{\prime }F_{9}^{\prime }F_{10}\;†\;F_{1}F_{2}^{\prime }F_{3}^{\prime }F_{4}F_{5}F_{6}F_{7}^{\prime }F_{8}F_{9}^{\prime }F_{10}\;†$
$F_{1}F_{2}^{\prime }F_{3}F_{4}F_{5}F_{6}^{\prime }F_{7}^{\prime }F_{8}F_{9}^{\prime }F_{10}\;†\;F_{1}F_{2}^{\prime }F_{3}^{\prime }F_{4}F_{5}F_{6}F_{7}F_{8}^{\prime }F_{9}^{\prime }F_{10}\;†\;F_{1}F_{2}^{\prime }F_{3}F_{4}F_{5}F_{6}^{\prime }F_{7}F_{8}^{\prime }F_{9}^{\prime }F_{10}\;†\;F_{1}F_{2}^{\prime }F_{3}F_{4}F_{5}^{\prime }F_{6}^{\prime }F_{7}F_{8}^{\prime }F_{9}^{\prime }F_{10}$

****************************************************

3-Run Beginnings (4-Parallel State Transition):

****************************************************

$F_{1}F_{2}^{\prime }F_{3}F_{4}F_{5}F_{6}^{\prime }F_{7}F_{8}^{\prime }F_{9}F_{10}\;†\;F_{1}F_{2}^{\prime }F_{3}F_{4}F_{5}F_{6}^{\prime }F_{7}^{\prime }F_{8}^{\prime }F_{9}F_{10}\;†\;F_{1}F_{2}F_{3}^{\prime }F_{4}F_{5}^{\prime }F_{6}F_{7}F_{8}^{\prime }F_{9}F_{10}\;†\;F_{1}F_{2}^{\prime }F_{3}F_{4}^{\prime }F_{5}^{\prime }F_{6}^{\prime }F_{7}^{\prime }F_{8}^{\prime }F_{9}F_{10}\;†$
$F_{1}F_{2}^{\prime }F_{3}^{\prime }F_{4}F_{5}F_{6}F_{7}F_{8}^{\prime }F_{9}F_{10}\;†\;F_{1}F_{2}^{\prime }F_{3}F_{4}^{\prime }F_{5}^{\prime }F_{6}^{\prime }F_{7}F_{8}^{\prime }F_{9}F_{10}\;†\;F_{1}F_{2}F_{3}^{\prime }F_{4}F_{5}^{\prime }F_{6}F_{7}^{\prime }F_{8}^{\prime }F_{9}F_{10}\;†\;F_{1}F_{2}^{\prime }F_{3}F_{4}F_{5}^{\prime }F_{6}F_{7}F_{8}^{\prime }F_{9}F_{10}\;†$
$F_{1}F_{2}^{\prime }F_{3}^{\prime }F_{4}F_{5}^{\prime }F_{6}^{\prime }F_{7}F_{8}^{\prime }F_{9}F_{10}\;†\;F_{1}F_{2}F_{3}^{\prime }F_{4}F_{5}^{\prime }F_{6}^{\prime }F_{7}F_{8}^{\prime }F_{9}F_{10}\;†\;F_{1}F_{2}^{\prime }F_{3}^{\prime }F_{4}F_{5}F_{6}^{\prime }F_{7}F_{8}^{\prime }F_{9}F_{10}\;†\;F_{1}F_{2}^{\prime }F_{3}^{\prime }F_{4}F_{5}F_{6}F_{7}^{\prime }F_{8}^{\prime }F_{9}F_{10}\;†$
$F_{1}F_{2}F_{3}^{\prime }F_{4}F_{5}^{\prime }F_{6}^{\prime }F_{7}^{\prime }F_{8}^{\prime }F_{9}F_{10}\;†\;F_{1}F_{2}^{\prime }F_{3}F_{4}^{\prime }F_{5}^{\prime }F_{6}F_{7}F_{8}^{\prime }F_{9}F_{10}\;†\;F_{1}F_{2}^{\prime }F_{3}F_{4}F_{5}^{\prime }F_{6}^{\prime }F_{7}F_{8}^{\prime }F_{9}F_{10}\;†\;F_{1}F_{2}^{\prime }F_{3}^{\prime }F_{4}F_{5}^{\prime }F_{6}F_{7}F_{8}^{\prime }F_{9}F_{10}\;†$
$F_{1}F_{2}^{\prime }F_{3}F_{4}F_{5}^{\prime }F_{6}F_{7}^{\prime }F_{8}^{\prime }F_{9}F_{10}\;†\;F_{1}F_{2}^{\prime }F_{3}F_{4}F_{5}F_{6}F_{7}^{\prime }F_{8}^{\prime }F_{9}F_{10}\;†\;F_{1}F_{2}^{\prime }F_{3}F_{4}^{\prime }F_{5}F_{6}F_{7}F_{8}^{\prime }F_{9}F_{10}\;†\;F_{1}F_{2}^{\prime }F_{3}F_{4}^{\prime }F_{5}F_{6}F_{7}^{\prime }F_{8}^{\prime }F_{9}F_{10}\;†$
$F_{1}F_{2}^{\prime }F_{3}F_{4}F_{5}F_{6}F_{7}F_{8}^{\prime }F_{9}F_{10}\;†\;F_{1}F_{2}^{\prime }F_{3}^{\prime }F_{4}F_{5}^{\prime }F_{6}F_{7}^{\prime }F_{8}^{\prime }F_{9}F_{10}\;†\;F_{1}F_{2}^{\prime }F_{3}F_{4}F_{5}^{\prime }F_{6}^{\prime }F_{7}^{\prime }F_{8}^{\prime }F_{9}F_{10}\;†\;F_{1}F_{2}^{\prime }F_{3}F_{4}^{\prime }F_{5}F_{6}^{\prime }F_{7}F_{8}^{\prime }F_{9}F_{10}\;†$
$F_{1}F_{2}^{\prime }F_{3}F_{4}^{\prime }F_{5}^{\prime }F_{6}F_{7}^{\prime }F_{8}^{\prime }F_{9}F_{10}\;†\;F_{1}F_{2}^{\prime }F_{3}^{\prime }F_{4}F_{5}F_{6}^{\prime }F_{7}^{\prime }F_{8}^{\prime }F_{9}F_{10}\;†\;F_{1}F_{2}^{\prime }F_{3}^{\prime }F_{4}F_{5}^{\prime }F_{6}^{\prime }F_{7}^{\prime }F_{8}^{\prime }F_{9}F_{10}\;†\;F_{1}F_{2}^{\prime }F_{3}F_{4}^{\prime }F_{5}F_{6}^{\prime }F_{7}^{\prime }F_{8}^{\prime }F_{9}F_{10}$

****************************************************

4-Run Beginnings (5-Parallel State Transition):

****************************************************

$F_{1}F_{2}^{\prime }F_{3}^{\prime }F_{4}F_{5}^{\prime }F_{6}F_{7}^{\prime }F_{8}F_{9}F_{10}\;†\;F_{1}F_{2}^{\prime }F_{3}^{\prime }F_{4}F_{5}F_{6}^{\prime }F_{7}^{\prime }F_{8}F_{9}F_{10}\;†\;F_{1}F_{2}^{\prime }F_{3}F_{4}^{\prime }F_{5}F_{6}^{\prime }F_{7}^{\prime }F_{8}F_{9}F_{10}\;†\;F_{1}F_{2}^{\prime }F_{3}^{\prime }F_{4}F_{5}^{\prime }F_{6}^{\prime }F_{7}^{\prime }F_{8}F_{9}F_{10}\;†$
$F_{1}F_{2}^{\prime }F_{3}F_{4}^{\prime }F_{5}^{\prime }F_{6}F_{7}^{\prime }F_{8}F_{9}F_{10}\;†\;F_{1}F_{2}^{\prime }F_{3}^{\prime }F_{4}F_{5}F_{6}F_{7}^{\prime }F_{8}F_{9}F_{10}\;†\;F_{1}F_{2}^{\prime }F_{3}F_{4}^{\prime }F_{5}^{\prime }F_{6}^{\prime }F_{7}^{\prime }F_{8}F_{9}F_{10}\;†\;F_{1}F_{2}^{\prime }F_{3}F_{4}^{\prime }F_{5}F_{6}F_{7}^{\prime }F_{8}F_{9}F_{10}\;†$
$F_{1}F_{2}^{\prime }F_{3}F_{4}F_{5}F_{6}^{\prime }F_{7}^{\prime }F_{8}F_{9}F_{10}\;†\;F_{1}F_{2}^{\prime }F_{3}F_{4}F_{5}^{\prime }F_{6}F_{7}^{\prime }F_{8}F_{9}F_{10}\;†\;F_{1}F_{2}^{\prime }F_{3}F_{4}F_{5}^{\prime }F_{6}^{\prime }F_{7}^{\prime }F_{8}F_{9}F_{10}\;†\;F_{1}F_{2}^{\prime }F_{3}F_{4}F_{5}F_{6}F_{7}^{\prime }F_{8}F_{9}F_{10}\;†$
$F_{1}F_{2}F_{3}^{\prime }F_{4}F_{5}^{\prime }F_{6}^{\prime }F_{7}^{\prime }F_{8}F_{9}F_{10}\;†\;F_{1}F_{2}F_{3}^{\prime }F_{4}F_{5}^{\prime }F_{6}F_{7}^{\prime }F_{8}F_{9}F_{10}$

****************************************************

5-Run Beginnings (6-Parallel State Transition):

****************************************************

$F_{1}F_{2}^{\prime }F_{3}^{\prime }F_{4}F_{5}F_{6}^{\prime }F_{7}F_{8}F_{9}F_{10}\;†\;F_{1}F_{2}^{\prime }F_{3}^{\prime }F_{4}F_{5}^{\prime }F_{6}^{\prime }F_{7}F_{8}F_{9}F_{10}\;†\;F_{1}F_{2}^{\prime }F_{3}F_{4}^{\prime }F_{5}^{\prime }F_{6}^{\prime }F_{7}F_{8}F_{9}F_{10}\;†\;F_{1}F_{2}^{\prime }F_{3}F_{4}F_{5}F_{6}^{\prime }F_{7}F_{8}F_{9}F_{10}\;†$
$F_{1}F_{2}^{\prime }F_{3}F_{4}^{\prime }F_{5}F_{6}^{\prime }F_{7}F_{8}F_{9}F_{10}\;†\;F_{1}F_{2}^{\prime }F_{3}F_{4}F_{5}^{\prime }F_{6}^{\prime }F_{7}F_{8}F_{9}F_{10}\;†\;F_{1}F_{2}F_{3}^{\prime }F_{4}F_{5}^{\prime }F_{6}^{\prime }F_{7}F_{8}F_{9}F_{10}$

****************************************************

6-Run Beginnings (7-Parallel State Transition):

****************************************************

$F_{1}F_{2}^{\prime }F_{3}F_{4}^{\prime }F_{5}^{\prime }F_{6}F_{7}F_{8}F_{9}F_{10}\;†\;F_{1}F_{2}^{\prime }F_{3}F_{4}F_{5}^{\prime }F_{6}F_{7}F_{8}F_{9}F_{10}\;†\;F_{1}F_{2}F_{3}^{\prime }F_{4}F_{5}^{\prime }F_{6}F_{7}F_{8}F_{9}F_{10}\;†F_{1}F_{2}^{\prime }F_{3}^{\prime }F_{4}F_{5}^{\prime }F_{6}F_{7}F_{8}F_{9}F_{10}$

****************************************************

7-Run Beginnings (8-Parallel State Transition):

****************************************************

$F_{1}F_{2}^{\prime }F_{3}F_{4}^{\prime }F_{5}F_{6}F_{7}F_{8}F_{9}F_{10}$

****************************************************

8-Run Beginnings (9-Parallel State Transition):

****************************************************

$F_{1}F_{2}^{\prime }F_{3}F_{4}F_{5}F_{6}F_{7}F_{8}F_{9}F_{10}$

****************************************************

9-Run Beginnings (10-Parallel State Transition):

**************************************************** -

****************************************************

10-Run Beginnings (11-Parallel State Transition):

****************************************************

$F_{1}F_{2}^{\prime }F_{3}^{\prime }F_{4}F_{5}F_{6}F_{7}F_{8}F_{9}F_{10}$
